# The Role of Intraligand Charge Transfer Processes in Iridium(III) Complexes with Morpholine-Decorated 4′-Phenyl-2,2′:6′,2″-terpyridine

**DOI:** 10.3390/molecules29133074

**Published:** 2024-06-27

**Authors:** Joanna Palion-Gazda, Aleksandra Kwiecień, Katarzyna Choroba, Mateusz Penkala, Anna Kryczka, Barbara Machura

**Affiliations:** Institute of Chemistry, University of Silesia, 9 Szkolna Str., 40-006 Katowice, Poland; aleksandra.a.kwiecien@us.edu.pl (A.K.); katarzyna.choroba@us.edu.pl (K.C.); mateusz.penkala@us.edu.pl (M.P.); akryczka1@us.edu.pl (A.K.)

**Keywords:** iridium(III) complexes, 4′-subsituted 2,2′:6′,2″-terpyridines, absorption and emission characteristics, intraligand and metal-to-ligand charge-transfer processes

## Abstract

To investigate the impact of the electron-donating morpholinyl (morph) group on the ground- and excited-state properties of two different types of Ir(III) complexes, [IrCl_3_(R-C_6_H_4_-terpy-κ^3^N)] and [Ir(R-C_6_H_4_-terpy-κ^3^N)_2_](PF_6_)_3_, the compounds [IrCl_3_(morph-C_6_H_4_-terpy-κ^3^N)] (**1A**), 4[Ir(morph-C_6_H_4_-terpy-κ^3^N)_2_](PF_6_)_3_ (**2A**), [IrCl_3_(Ph-terpy-κ^3^N)] (**1B**) and [Ir(Ph-terpy-κ^3^N)_2_](PF_6_)_3_ (**2B**) were obtained. Their photophysical properties were comprehensively investigated with the aid of static and time-resolved spectroscopic methods accompanied by theoretical DFT/TD-DFT calculations. In the case of bis-terpyridyl iridium(III) complexes, the attachment of the morpholinyl group induced dramatic changes in the absorption and emission characteristics, manifested by the appearance of a new, very strong visible absorption tailing up to 600 nm, and a significant bathochromic shift in the emission of **2A** relative to the model chromophore. The emission features of **2A** and **2B** were found to originate from the triplet excited states of different natures: intraligand charge transfer (^3^ILCT) for **2A** and intraligand with a small admixture of metal-to-ligand charge transfer (^3^IL–^3^MLCT) for **2B**. The optical properties of the mono-terpyridyl iridium(III) complexes were less significantly impacted by the morpholinyl substituent. Based on UV–Vis absorption spectra, emission wavelengths and lifetimes in different environments, transient absorption studies, and theoretical calculations, it was demonstrated that the visible absorption and emission features of **1A** are governed by singlet and triplet excited states of a mixed MLLCT-ILCT nature, with a dominant contribution of the first component, that is, metal-ligand-to-ligand charge transfer (MLLCT). The involvement of ILCT transitions was reflected by an enhancement of the molar extinction coefficients of the absorption bands of **1A** in the range of 350–550 nm, and a small red shift in its emission relative to the model chromophore.

## 1. Introduction

4′-subsituted 2,2′:6′,2″-terpyridines (R-terpys) are among the most important building blocks in coordination chemistry [1,2,3,4,5,6]. When combined with numerous transition metal ions, they provide opportunities to obtain mono- and polynuclear coordination compounds suitable for applications in optoelectronics [7,8,9,10,11,12], photocatalysis [1,13,14,15,16,17,18], chemotherapy, and photodynamic therapy [19,20,21,22,23,24,25,26,27]. Thanks to the highly convenient Krönhke methodology [28], the functional properties of R-terpys and their transition metal complexes can be widely tuned, allowing for further progress in the improvement of phosphorescent materials and potent chemotherapeutic drugs. 

Recently, significant effort has been dedicated to understanding the impact of substituents attached via aryl linkers to the central pyridine ring of the terpy backbone. The crucial role of the remote substituent of R-C_6_H_4_-terpys in controlling the ground- and excited-state properties of [ReCl(CO)_3_(R-C_6_H_4_-terpy-κ^2^N)] was confirmed by Fernández-Terán [14,29] and our research groups [30,31,32,33,34,35,36]. The optical properties of [ReCl(CO)_3_(R-C_6_H_4_-terpy-κ^2^N)] were found to systematically vary with increasing electron-donating abilities of the appended groups (–CN < –CF_3_ < –Br < –H < –OMe), as manifested by a hypsochromic shift of the metal-ligand-to-ligand charge transfer (^1^MLLCT) absorption and ^3^MLLCT emission bands, increased ^3^MLLCT lifetimes, and ΔG_S-T_ from –CN to –OMe. The introduction of the stronger electron-donating group –NMe_2_ resulted in a switch of the excited state from ^3^MLLCT (with –CN, –CF_3_, –Br, and –OMe groups) to intraligand charge transfer ^3^ILCT (–NMe_2_), which was reflected in a dramatically enhanced visible light absorption band, prolonged excited-state lifetime in solution, red shift of the emission upon cooling, and improved photocatalytic properties of [ReCl(CO)_3_(Me_2_N-C_6_H_4_-terpy-κ^2^N)] in hydrogen evaluation experiments [14]. As demonstrated by our research group [36], the photoinduced processes in [ReCl(CO)_3_(R-C_6_H_4_-terpy-κ^2^N)] with remote electron-donating N-methyl-piperazinyl and (2-cyanoethyl)methylamine groups were additionally affected by the polarity environment. Increased solvent polarity favored the population of the ^3^ILCT excited state, leading to much more extended triplet excited-state lifetimes in polar solvents. Conversely to those of [ReCl(CO)_3_(R-C_6_H_4_-terpy-κ^2^N)], the optical properties of the Re(I) carbonyl complexes with meridonally co-ordinated 4′-subsituted 2,2′:6′,2″-terpyridines [ReCl(CO)_2_(Me_2_N-C_6_H_4_-terpy-κ^3^N)] were governed by MLLCT excited states, independent of the electron-donating abilities of the pendant group of R-C_6_H_4_-terpy. The more electron-rich {Re(CO)_2_}^+^ moiety in [ReX(CO)_2_(R-C_6_H_4_-terpy-κ^3^N)] systems was found to hinder access to ILCT excited states [14]. 

**Scheme 1:**
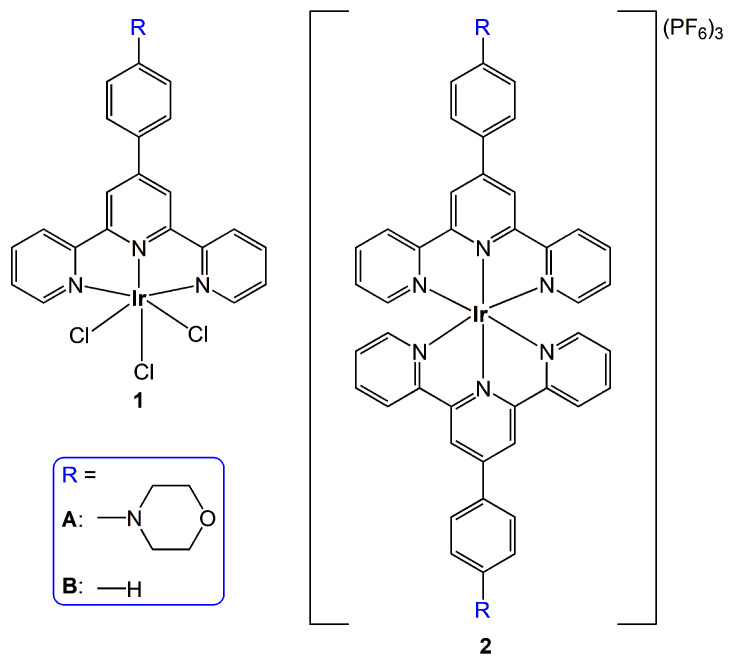
Chemical structures of investigated Ir(III) compounds.

In the current work, we investigated the effect of the electron-donating morpholinyl (morph) group on ground- and excited-state properties of two different types of Ir(III) complexes: [IrCl_3_(R-C_6_H_4_-terpy-κ^3^N)] and [Ir(R-C_6_H_4_-terpy-κ^3^N)_2_](PF_6_)_3_ (Scheme 1).

The photophysical properties of [IrCl_3_(morph-C_6_H_4_-terpy-κ^3^N)] (**1A**) and [Ir(morph-C_6_H_4_-terpy-κ^3^N)_2_](PF_6_)_3_ (**2A**) were comprehensively explored using static and time-resolved spectroscopic methods, accompanied by theoretical DFT/TD-DFT calculations, and analyzed in comparison to the photobehavior of the corresponding parent chromophores [IrCl_3_(Ph-terpy-κ^3^N)] (**1B**) and [Ir(Ph-terpy-κ^3^N)_2_](PF_6_)_3_ (**2B**), which was partially discussed in [37,38]. We demonstrated that the morpholinyl remote group has a significantly larger impact on the photophysical behavior of the bis-terpyridyl Ir(III) complex.

## 2. Results and Discussion

**Figure 1:**
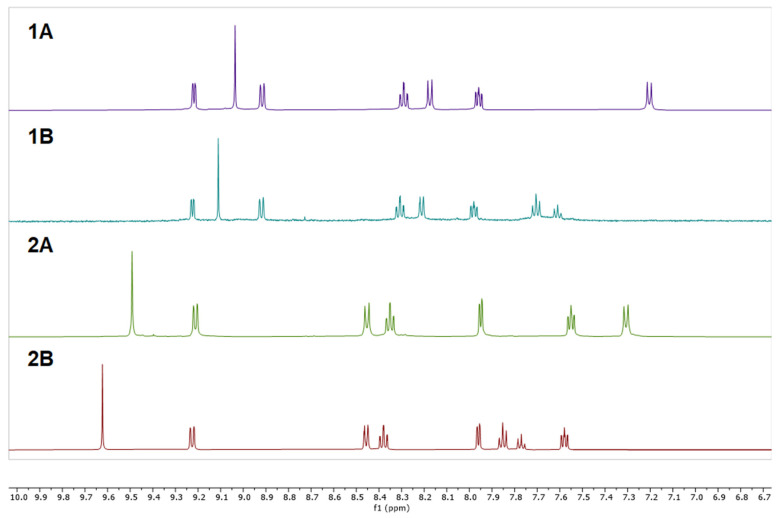
Proton signals of Ir(III) complexes.

The complexes [IrCl_3_(R-C_6_H_4_-terpy-κ^3^N)] (**1A** and **1B**) and [Ir(R-C_6_H_4_-terpy-κ^3^N)_2_](PF_6_)_3_ (**2A** and **2B**) were prepared using conventional synthetic procedures [39,40,41]. Specifically, the iridium(III) chloride salt was heated with one equivalent of the appropriate R-C_6_H_4_-terpy ligand in methoxyethanol to synthesize **1A** and **1B**. The corresponding [IrCl_3_(R-C_6_H_4_-terpy-κ^3^N)] complex was then reacted with a slight excess of R-C_6_H_4_-terpy in ethylene glycol to obtain **2A** and **2B**. The identity and composition of **1**–**2** was confirmed by elemental analysis, ^1^H and ^13^C NMR spectroscopies (Figure 1 and Figures S1–S4), and the FT-IR technique (Figure S5). 

As demonstrated in Figure 1, the proton signals of the central pyridine ring in the terpyridine backbone (represented by the singlet) were noticeably shifted upfield in the morpholinyl-substituted Ir(III) systems, indicating that the appended electron-donating morpholine group affects the electron density in the distal part of the ligand molecule. The difference in chemical shifts, from 9.11 ppm for **1B** to 9.04 ppm for **1A** and from 9.62 ppm for **2B** to 9.49 for **2A**, shows that this effect was stronger for the cationic Ir(III) complexes.

The FT-IR spectra of all the investigated Ir(III) complexes displayed characteristic bands in the region 1615–1525 cm^−1^, attributable to ν(C=N)_terpy_ and ν(C=C)_terpy_ stretches. The intense bands occurring at ~840 cm^−1^ and ~555 cm^−1^ in the FT-IR spectra of **2A** and **2B** are indicative of PF_6_^−^ ions (Figure S5) [42].

**Figure 2:**
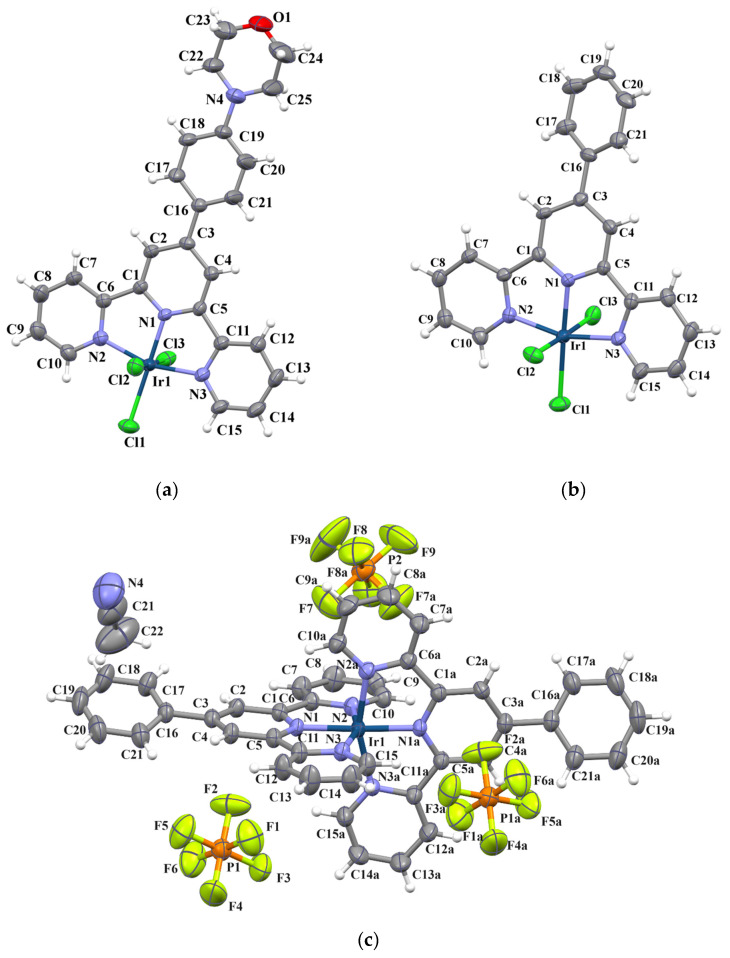
Molecular structures of **1A** (**a**), **1B** (**b**), and **2B** (**c**), with the atom numbering and thermal ellipsoids set at 50% probability for non-hydrogen atoms. (Symmetry code for **2B**: (a) = 2 − *x*, *y*, 3/2 − *z*).

Additionally, the molecular structures of **1A**, **1B**, and **2B** were unambiguously determined by X-ray analysis. The full structural data of these systems are provided in the Supplementary Materials (Tables S1–S4 and Figure S6). Perspective views of the molecular structures of **1A**, **1B**, and **2B**, with atom numbering, are depicted in Figure 2, while the most relevant bond lengths and angles are summarized in Table 1.

In all reported complexes, the Ir(III) ion was located in a distorted octahedral environment. The coordination sphere of **1A** and **1B** was defined by three chloride ions and three nitrogen atoms of the tridendate-coordinated R-C_6_H_4_-terpy (κ^3^N) ligand. Due to the geometrical constraints imposed by the planar terpy framework, both nitrogen and chlorine donor atoms adopted meridional arrangements. The crystal structure of **2B** consisted of the complex cations [Ir(Ph-terpy-κ^3^N)_2_]^3+^, PF_6_^−^ counteranions in a 1:3 molar ratio, and solvent (MeCN) molecules. The metal center of [Ir(Ph-terpy-κ^3^N)_2_]^3+^ was octahedrally surrounded by six nitrogen atoms from two Ph-terpy-κ^3^N ligands, coordinated in a *mer*-fashion. 

Typical of the terpy-κ^3^N coordination mode [43], the Ir–N bond lengths involving peripheral pyridine rings in structures **1A**, **1B**, and **2B** were longer than the Ir–N_central pyridine_ distances (Table 1). The shortening of the Ir–N_central pyridine_ bond length was rationalized by a more efficient overlap of the t_2g_ metal orbitals with the π* orbitals of the central pyridine relative to the peripheral pyridyl groups. Consequently, the chloride ligand *trans*-located to the central pyridine ring of R-C_6_H_4_-terpy in molecules **1A** and **1B** exhibited an elongated bond distance in relation to the other Ir–Cl ones (Table 1). 

In all the studied Ir(III) complexes, a considerable angular distortion from the octahedral geometry was reflected in the N–Ir–N bite angles due to the formation of five-membered metallocycles upon the chelating coordination of R-C_6_H_4_-terpy. The N–Ir–N bite angles varied from 79.84(12) to 80.99(16)° (Table 1). The terpy framework in structures **1A**, **1B**, and **2B** showed good planarity, with the dihedral angles between the mean planes of the central pyridine and terminal aromatic rings ranging from 1.26° to 7.92°. The plane of the phenyl ring of **1A** maintained near co-planarity (1.92°) with the central pyridine plane, while in the model compounds, it was inclined to the central pyridine by 20.48° in **1B**, and 23.46° in **2B**. 

**Figure 3:**
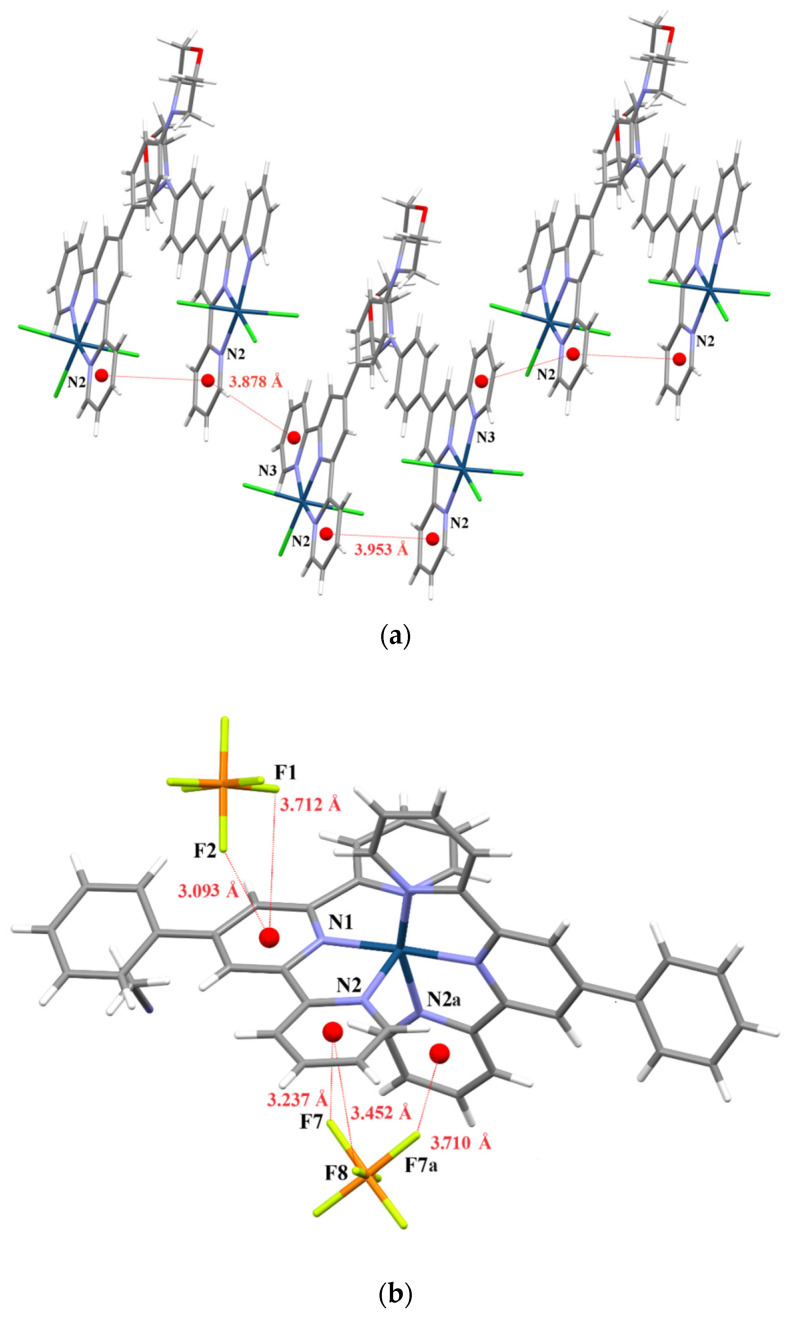
View of the supramolecular chain of [IrCl_3_(morph-C_6_H_4_-terpy-κ^3^N)]_n_ resulting from weak π•••π interactions indicated by red dashed line (**a**); C–H•••F and P–F•••π contacts between the cations [Ir(Ph-terpy-κ^3^N)_2_]^3+^ and anions PF_6_^−^ in the structure **2B** (**b**) (see also Figure S6 and Tables S2–S4).

The arrangement of molecules [IrCl_3_(R-C_6_H_4_-terpy-κ^3^N)] in the crystal structures of **1A** and **1B** was governed by π•••π stacking interactions and weak hydrogen bonds (C–H•••Cl and C–H•••O). The crystal packing analysis of **2B** revealed interactions between the cations [Ir(Ph-terpy-κ^3^N)_2_]^3+^ and anions PF_6_^−^, facilitated by weak hydrogen bonds (C–H•••F) and P–F•••π contacts (Figure 3 and Tables S2 and S4). The shortest Ir•••F distances were 5.542 Å and 5.940 Å.

## 3. Ground-State Molecular Orbitals

**Figure 4:**
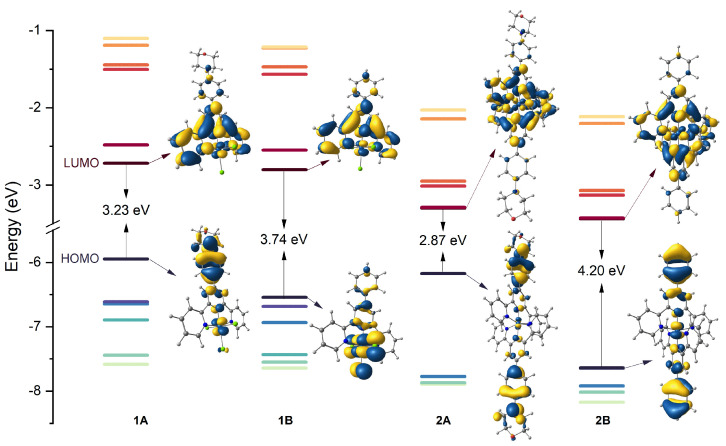
The partial molecular orbital energy level diagram with the plots of electron density distributions in the HOMO and LUMO of **1A**–**2A** and **1B**–**2B**.

To better understand the impact of the electron-donating morpholinyl group on the photophysical behavior of the [IrCl_3_(R-C_6_H_4_-terpy-κ^3^N)] (**1A**, **1B**) and [Ir(R-C_6_H_4_-terpy-κ^3^N)_2_](PF_6_)_3_ (**2A**, **2B**) systems, the energies and electron distributions of the highest occupied (HOMO) and lowest unoccupied (LUMO) molecular orbitals are briefly presented prior to the discussion of the optical properties of **1A**–**2A** and **1B**–**2B**. DFT calculations were performed using Gaussian-16 software [44] at the TD-DFT/PCM/PBE0/SDD/def2-TZVP level [45] (Figure 4, Tables S5–S7, Figure S7). 

As shown in Figure 4, the attachment of the morpholinyl group to Ph-terpy led to the destabilization of both the HOMO and LUMO orbitals, with more pronounced energy variations in the case of the HOMO level. In the pairs **1A**–**1B** and **2A**–**2B**, the HOMO energy increased by ~0.60 eV and ~1.47 eV upon going from the unsubstituted to the morpholinyl-decorated system, respectively. The destabilization of the LUMO level due to the morpholine incorporation was ~0.08 eV and ~0.14 eV for pairs **1A**–**1B** and **2A**–**2B**. Consequently, the Ir(III) complexes with the morph-C_6_H_4_-terpy ligand (**1A** and **2A**) exhibited significantly reduced HOMO–LUMO gaps relative to their reference complexes (**1B** and **2B**). The HOMO–LUMO energy gap decreased in the order of **2B** (4.20 eV) > **1B** (3.74 eV) > **1A** (3.23 eV) > **2A** (2.87 eV). 

Notably, the replacement of three electron-donating halide ions in [IrCl_3_(R-C_6_H_4_-terpy-κ^3^N)] by the π-accepting R-C_6_H_4_-terpyκ^3^N ligand in [Ir(R-C_6_H_4_-terpy-κ^3^N)_2_]^3+^resulted in the stabilization of the HOMO and LUMO levels of the bis-terpyridyl systems. In the pairs **1A**–**2A** and **1B**–**2B**, the HOMO energy decreased by ~0.22 eV and ~1.1 eV, respectively. In turn, the LUMO energy of **1A** and **1B** dropped by ~0.58 eV and ~0.64 eV compared to **2A** and **2B**, respectively.

As expected, the morpholine substituent also altered the nature of the HOMO orbital. Unlike the HOMO of **1B**, which predominantly comprised the orbitals of the Ir(III) center (61.8 %) and chloride ions (27.4 %), the HOMO of **1A** was a combination of morpholine (32.1%), phenylene (48.1%), and terpy (9.5%) orbitals. Compared to **1B**, the metal character of the HOMO of **1A** decreased from 61.8 % to 8.5 %. The highest occupied molecular orbitals of **2A** and **2B** resided almost exclusively on the R-C_6_H_4_-terpy ligand. For morpholinyl-substituted complex (**2A**), it comprised the morpholine group (35.9 %) and phenylene linker (46.5%). The metal contributions in the HOMO of **2A** and **2B** were 2.1% and 12.5%, respectively. The lowest unoccupied molecular orbital of all the studied Ir(III) complexes was predominately constituted by π* orbitals of the terpy backbone. 

## 4. Photophysical Properties–Experimental and Theoretical Insights

**Figure 5:**
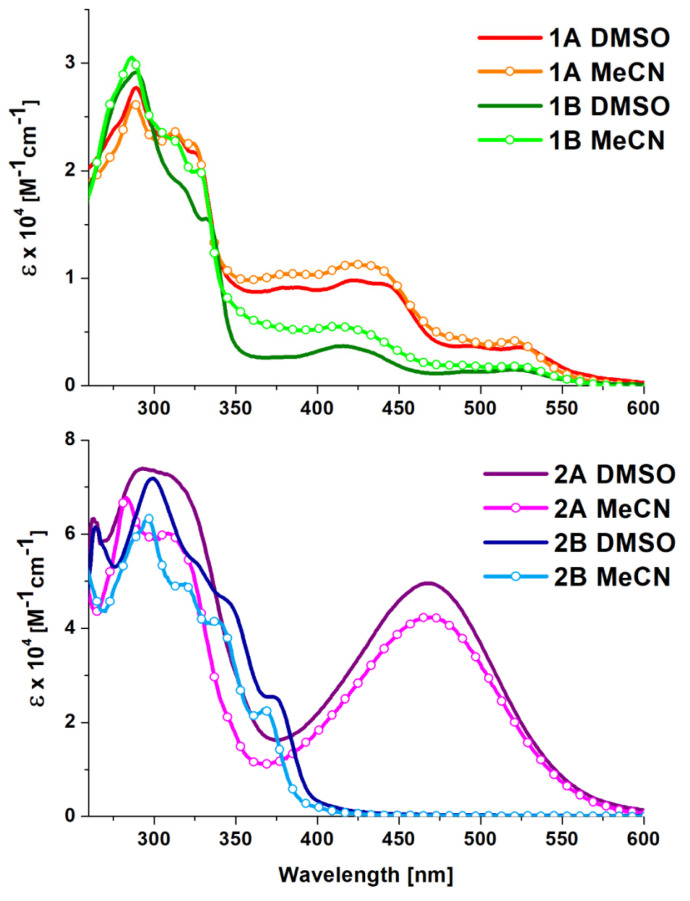
UV–Vis spectra of Ir(III) complexes recorded in MeCN and DMSO solutions.

The electronic absorption properties of [IrCl_3_(R-C_6_H_4_-terpy-κ^3^N)] and [Ir(R-C_6_H_4_-terpy-κ^3^N)_2_](PF_6_)_3_ were studied in MeCN and DMSO (Figure 5 and Figure S8). The complexes **1** and **2** showed insufficient solubility in less polar solvents. The absorption band maxima and molar extinction coefficients of **1**–**2** are summarized in Table S8.

As demonstrated in Figure 5, the model complex **1B** exhibited intense high-energy absorption bands in the range of 225–350 nm, which were attributed to π–π* transitions of the Ph-terpy ligand, and noticeably weaker absorptions with maxima above 350 nm, which were tentatively assigned to charge-transfer transitions ^1^MLLCT with a possible admixture of spin-forbidden singlet-triplet ^3^MLLCT due to the high spin-orbit coupling constant of iridium [46,47,48]. In contrast, the complex **2B** principally absorbed wavelengths below 400 nm. As previously established for related systems [37,41,49,50,51,52], these UV bands of **2B** were predominantly attributed to ^1^IL transitions within the coordinated Ph-terpy ligands. The very low magnitudes of molar extinction coefficients for the absorptions of **2B** in the visible region (Table S8 and Figure S8) are consistent with spin-forbidden singlet-triplet ^3^MLCT transitions [49,53].

Within the series [IrCl_3_(R-C_6_H_4_-terpy-κ^3^N)], the attachment of the electron-donating morpholinyl group to the phenyl ring of 2,2′:6′,2″-terpyridine evoked a minor effect on the absorption energies but led to a significant enhancement of the molar extinction coefficients of absorptions bands in the range of 350–550 nm (Table S8 and Figure 5). In contrast, dramatic changes in the absorption characteristics were observed for **2A** in relation to **2B**. The morpholinyl-substituted complex (**2A**) was deeply red and displayed a very strong absorption tailing up to 600 nm, which was absent in the UV–Vis spectrum of its parent chromophore **2B**, which was pale yellow in solution. The molar extinction coefficient of this band exceeded 4 × 10^4^ M^−1^·cm^−1^ in solution. 

**Figure 6:**
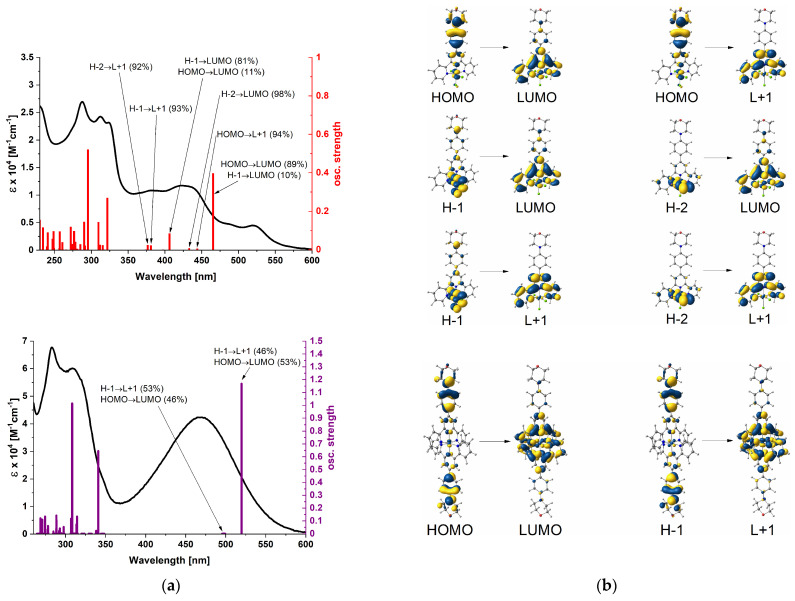
Experimental absorption spectra (black line) of **1A** and **2A** alongside vertical lines presenting singlet–singlet transitions with corresponding oscillator strengths, computed at the TD-DFT/PCM/PBE0/SDD/def2-TZVP level with the use of the PCM model at polarities corresponding to MeCN (**a**); electron density distributions plots of frontier low energy transitions (**b**). The data for parent compounds **1B** and **2B** are provided in Table S6 in the Supplementary Materials.

The character of electronic transitions underlying the absorption features of [IrCl_3_(R-C_6_H_4_-terpy-κ^3^N)] and [Ir(R-C_6_H_4_-terpy-κ^3^N)_2_](PF_6_)_3_ was also investigated theoretically at the DFT/PCM/PBE0/SDD/def2-TZVP level (Figure 6). The calculations confirmed that the changes in the absorption characteristics of the morpholinyl-substituted Ir(III) complexes (**1A** and **2A**) relative to the parent chromophores (**1B** and **2B**) were due to the involvement of ^1^ILCT transitions, occurring from the electron-donating morpholinyl group to the π-acceptor terpy unit. The low-energy absorptions (above 350 nm) of **1A** mainly originated from the excitations HOMO→LUMO, HOMO→L + 1, H–1→LUMO, H–1→L + 1, H-2→LUMO, and H–2→L + 1. Based on the percentage composition of the molecular orbitals, the transitions HOMO→LUMO and HOMO→L + 1 can be designated as ^1^ILCT/^1^IL, while the other ones correspond to the ^1^MLLCT excitations. The low-energy absorptions of **1B** only comprised ^1^MLLCT transitions (Table S6). Some discrepancies between the experimental and theoretical electronic spectra of **1A** and **1B** in the low energy part may be rationalized by the possible involvement of spin-forbidden singlet-triplet transitions, which are evidenced by the TD-DFT calculations [46]. The strong visible absorption of **2A** was dominated by the transitions HOMO→LUMO and HOMO–1→LUMO + 1 of prevailing ILCT nature. Both HOMO and HOMO-1 comprised the morpholine group and phenylene linker, while LUMO and LUMO + 1 resided predominately on the terpy backbone. 

**Figure 7:**
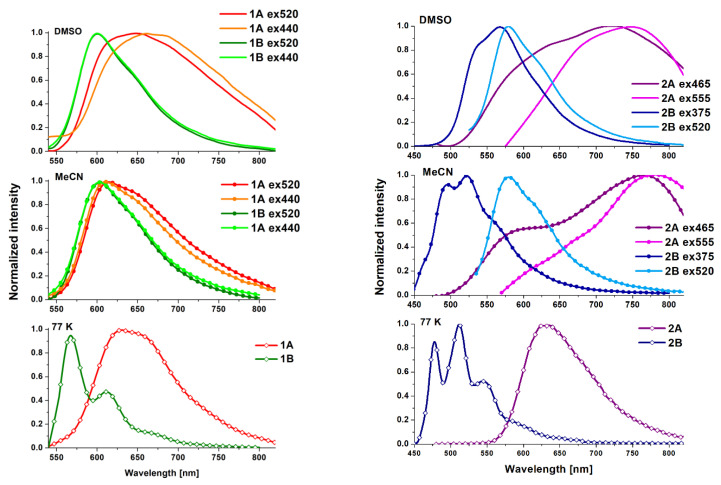
Normalized emission spectra of pairs **1A**–**1B** and **2A**–**2B** in DMSO and MeCN solutions at RT, and MeOH/EtOH (1:4 *v*:*v*) rigid matrix at 77 K.

Prior to the investigations of the excited-state properties of **1**–**2**, their stability and photostability in solution were confirmed by UV–Vis spectroscopy, as demonstrated in Figures S9 and S10. The photoluminescence properties of the Ir(III) complexes were explored in MeCN and DMSO solutions at room temperature (RT), in a solid state, and in a frozen matrix of EtOH/MeOH (4:1 *v*/*v*) at 77 K, as presented in Figure 7 and Table 2. Some additional photophysical data of **1**–**2** are provided in the Supplementary Materials (Figures S11–S18). 

For all the studied complexes, their lifetimes were in the microsecond or sub-microsecond domain (Table 2), and the photoluminescence intensities and lifetimes were sensitive to oxygen quenching, supporting the idea that the observed emission originates from a triplet excited state (Figures S11 and S12).

The complexes **1A** and **1B** in deaerated MeCN and DMSO solutions showed unstructured emission bands. Compared to the model chromophore **1B**, the phosphorescence band of **1A** was noticeably broader and appeared at a lower energy, with the emission maximum red-shifted by 50 nm for the DMSO solution and 10 nm for MeCN upon excitation at 520 nm. A bathochromic shift in the emission maximum of **1A** was accompanied by a slight decrease in the excited-state lifetime relative to **1B** (Table 2). 

In contrast to **1B**, which showed excitation-independent emission, the energy of the emission of **1A** was affected by excitation wavelengths, becoming noticeably red-shifted upon wavelength excitations in the range of 400–525 (Figure S13). A difference between **1A** and **1B** was also noticed when the frozen-state emissions were considered. Upon cooling to 77 K, the emission band of **1A** remained structureless and appeared almost in the same range as the RT emission band, while the frozen-state emission band of **1B** exhibited well-resolved vibronic progressions and appeared at a higher energy in relation to that in solution, as shown in Figure 7. All these findings indicate that the complex **1B** emits from a triplet excited state of ^3^MLLCT character with a minor contribution of ^3^π→π*_terpy_ transitions (^3^IL), while the emissive triplet excited state of the morpholinyl-substituted Ir(III) analog (**1A**) has a mixed nature ^3^MLLCT–^3^ILCT. By analogy to previous findings [14,29,30,31,32,33,34,35,36], the contribution of ^3^ILCT in the triplet excited state of **1A** manifested in a bathochromic shift of the emission in solution at RT and frozen matrix EtOH/MeOH relative to the parent chromophore. Transition metal complexes functionalized with electron-rich groups, which emit from the ^3^MLLCT excited state, are expected to show a hypsochromic shift compared to the unsubstituted model compounds [14,29]. 

For the bis-terpyridyl iridium(III) complexes, the morpholinyl group induced dramatic changes in the emission characteristics (Figure 7 and Figures S14–S17). In solution at RT, the model chromophore **2B** displayed a green emission. Its emission band exhibited a weak vibronic structure, which became well resolved upon cooling to 77 K. The frozen-state emission band of **2B** appeared almost in the same range as that in MeCN at RT upon excitations of ≤375 nm, but it was slightly blue-shifted in relation to its emission in DMSO (Figure 7 and Figure S14). The differences in the emission energies and spectral profiles of **2B** in MeCN and DMSO can be rationalized by the fact that the emission of [Ir(R-terpy-κ^3^N)_2_](PF_6_)_3_ systems in solution may occur from the triplet excited state of the cation [Ir(R-terpy-κ^3^N)_2_]^3+^ and ion-pair adduct [Ir(R-terpy-κ^3^N)_2_]^3+^·PF_6_^−^ [41,50,54,55,56,57]. While the emission of **2B** in MeCN upon excitations of ≤375 nm can be attributed to the phosphorescence of [Ir(R-terpy-κ^3^N)_2_]^3+^, the ion-pair formation quenches the phosphorescence of [Ir(R-terpy-κ^3^N)_2_]^3+^ in DMSO. The observed emission of **2B** in DMSO originated mainly from the ion-pair adduct [Ir(R-terpy-κ^3^N)_2_]^3+^·PF_6_^−^. In acetonitrile solution, the emission from the ion-pair adduct occurred using longer excitation wavelengths (Figure S14), as previously reported for the related complex [Ir(terpy-κ^3^N)_2_](PF_6_)_3_ [57]. It is worth noting that there were also two alternative explanations of the photoluminescence behavior of [Ir(R-terpy-κ^3^N)_2_]^3+^ cations in previous reports. The emission properties of such systems are attributed to the triplet ligand-centered (^3^IL) or mixed ^3^IL–^3^MLCT excited states [39]. Regarding the first assignment, the lack of the strong vibronic progression of the emission band at RT, typical of ^3^IL emission, is rationalized by the thermal distribution of conformers with different torsion angles between the terpy core and aryl pendant group, leading to a less well-resolved emission band [39]. The transient absorption findings, discussed in the next section, indicate that the emission of **2B** is not of pure of ^3^IL phosphorescence, but rather occurs from the triplet excited state of the mixed ^3^IL–^3^MLCT character.

The emission spectrum of the morpholinyl-substituted complex (**2A**) was dominated by the structureless band in the NIR region, with the maximum at 760 nm in MeCN and 750 nm in DMSO, red-shifted by ~240 and 180 nm relative to the model complex **2B**, respectively. A bathochromic shift in the emission of **2A** was accompanied by a noticeable decrease in the excited-state lifetime relative to **2B** (Table 2). The shoulder/band at higher energies of **2A** can be assigned the reabsorption or emission from the ^3^MLLCT excited state (Figure S14). The frozen-state emission of **2A** remained non-structured and occurred in a higher energy region (630 nm) compared to its room-temperature emission. The photoluminescence characteristics of **2A** are typical of the emission occurring from ^3^ILCT excited state [58,59,60,61]. It is worth noting that NIR emitters are of high significance due to their potential applications in biomedical imaging [62,63,64]. 

**Figure 8:**
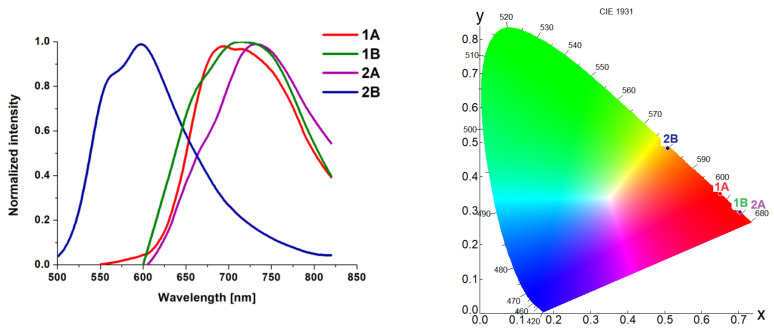
Normalized emission spectra of pairs **1A**–**1B** and **2A**–**2B** in the solid state alongside the CIE chromaticity diagram.

The photoluminescence properties of **1**–**2** were also investigated in the solid state. As demonstrated in Figure 8, the solid-state triplet emission band of **2B** occurred in a higher energy region (orange-yellow) and was characterized by a noticeably prolonged lifetime (~10 μs) compared to other studied complexes, showing emission in the red wavelength range, with lifetimes in the sub-microsecond domain (Table 2). 

**Figure 9:**
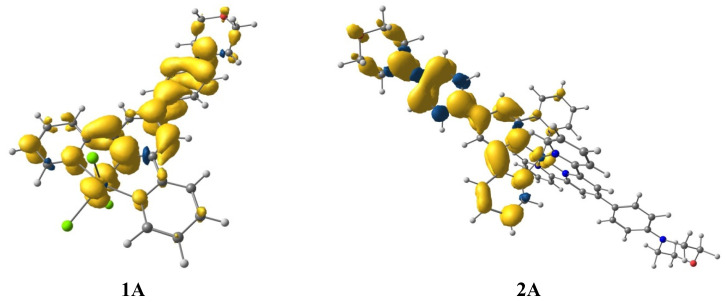
Spin density surface plots for **1A** and **2A**. For parent chromophores, spin density surface plots are provided in the Supplementary Materials.

To theoretically investigate the excited-state properties of complexes **1**–**2**, their geometries were optimized in triplet states (T_1_) and triplet energies were calculated from the energy difference between the ground singlet and triplet excited states $\Delta E_{T_{1}-S_{0}}$. The theoretically determined triplet emissions, 694 nm for **1A**, 590 nm for **1B**, 832 nm for **2A**, and 540 nm for **2B**, are in satisfactory agreement with the experimental values (Table 2 and Table S7). Regarding the spin density surface plots (Figure 9 and Figure S7), the emission in the complexes **1A**, **1B**, **2A**, and **2B** occurred due to the triplet excited state of an MLCT-ILCT, MLLCT, ILCT, and IL-MLCT nature, respectively.

## 5. Femtosecond Transient Absorption Spectra

**Figure 10:**
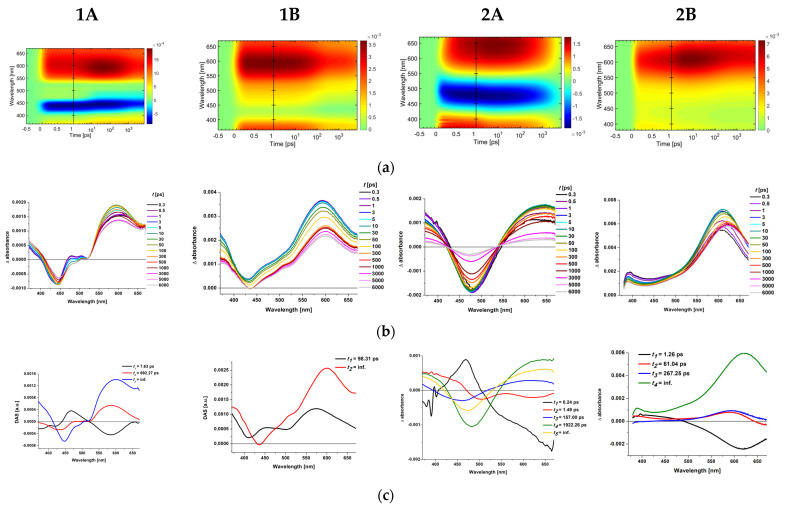
The results of fs-TA measurements and global fitting analyses: (**a**) 2D time–wavelength plots; (**b**) fs-TA spectra at selected time delays (ps); (**c**) decay-associated spectra DAS_i_ with appropriate time constant t_i_ (see also Figures S19–S22).

To further understand the triplet excited state characteristics of the studied complexes, femtosecond transient absorption (fs-TA) measurements were recorded in deaerated DMSO (**1A** and **1B**) and MeCN (**2A** and **2B**) solutions upon excitation at 355 nm. TA solutions were prepared at concentrations of 50–250 μM to provide an absorbance of ~0.5 in 2 mm path length quartz cells at the excitation wavelength. The results of the fs-TA measurements and global fitting analyses are summarized in Figure 10 and Figures S19–S23. The photostability of the Ir(III) complexes during the TA experiments was verified by comparing their UV–Vis spectra recorded before and after irradiation (Figure S24). 

The complexes **1A** and **2A** exhibited distinct transient absorption (TA) features compared to their parent chromophores (**1B** and **2B**), indicating that the introduction of the electron-donating morpholinyl group significantly affected the nature of the lowest triplet excited state of [IrCl_3_(R-C_6_H_4_-terpy-κ^3^N)] and [Ir(R-C_6_H_4_-terpy-κ^3^N)_2_](PF_6_)_3_. Specifically, **1A** and **2A** displayed ground-state bleaching (GSB) in wavelength regions corresponding to their visible charge-transfer absorptions, along with excited-state absorption bands (ESA) in the ranges of 375–415 nm and 522–670 nm for **1A**, and 375–425 nm and 540–670 nm for **2A**. In contrast, the TA spectra of the parent chromophores displayed only positive bands across the UV and visible regions, indicating that **1B** and **2B** have a stronger triplet excited-state absorption than the ground-state absorption [65,66,67,68]. For all the studied Ir(III) complexes, the TA signals appeared promptly after photoexcitation at 355 nm. After vibrational cooling, solvation, and geometrical relaxation within the triplet manifold, these signals persisted up to the end of the delay stage (7 ns). Ultrafast intersystem crossing occurs on a time scale shorter than the instrument response and is not detected with our experimental setup [48].

The comparison of the TA spectral profiles of **2A** and **2B** (Figure S23) indicated the involvement of different triplet excited states in their transient absorptions. For complex **2A**, the TA spectra comprised two ESA bands separated by a strong GSB signal, with two well-defined isosbestic points at 425 and 540 nm. The decay of both ESA bands and the recovery of the GSB occurred on the same time scale. The ESA band in the UV range was attributed to the terpyridyl anion radical [69,70], associated with the ^3^ILCT excited state, represented by the ESA with a maximum at 645 nm. The triplet excited state was of a predominantly ^3^ILCT nature.

The fs-TA data of **2B** indicated a mixed ^3^IL-^3^MLCT nature. With reference to the TA findings for [Ir(terpy-κ^3^N)_2_](PF_6_)_3_ [51], the higher energy TA signal suggests a ^3^IL_terpy_ triplet excited state, while the lower energy one can be assigned to the ^3^MLCT transient absorption. The presence of two different transient species undergoing interconversion was supported by the isosbestic point at 515 nm in the TA spectrum of **2B**. Additionally, the lower energy ESA band of **2A** was similar to the low energy ESA band of the model chromophore **1B**, characterized by the lowest triplet excited state of a ^3^MLLCT nature (Figure S23). 

The comparison of the TA spectral profiles of **1A** and **1B** (Figure S23) demonstrated that the morpholinyl group induces smaller variations in the TA spectral features of [IrCl_3_(R-C_6_H_4_-terpy-κ^3^N)]. The presence of a GSB band, the broadening of the visible ESA band, and different decay kinetics relative to **1B** suggest that the TA spectral features of **1A** correspond to a triplet excited state of a mixed ^3^MLLCT-^3^ILCT nature, with a dominant contribution of the first component.

## 6. Conclusions

The work presents comprehensive studies of [IrCl_3_(morph-C_6_H_4_-terpy-κ^3^N)] (**1A**), [Ir(morph-C_6_H_4_-terpy-κ^3^N)_2_](PF_6_)_3_ (**2A**), [IrCl_3_(Ph-terpy-κ^3^N)] (**1B**), and [Ir(Ph-terpy-κ^3^N)_2_](PF_6_)_3_ (**2B**), which were designed to explore the impact of the electron-donating morpholinyl (morph) group on the ground- and excited-state properties of two different types of Ir(III) complexes: [IrCl_3_(R-C_6_H_4_-terpy-κ^3^N)] and [Ir(R-C_6_H_4_-terpy-κ^3^N)_2_](PF_6_)_3_. We demonstrated that the attachment of morpholine leads to a change in the nature of the singlet and triplet excited states of bis-terpyridyl iridium(III) complexes, switching from intraligand with a small admixture of metal-to-ligand charge transfer (IL–MLCT) for **2B** to intraligand charge transfer (ILCT) in **2A**. Consequently, the compounds **2A** and **2B** exhibited completely different absorption and emission features. The deeply red complex **2A** displayed very strong absorption tailing up to 600 nm, which was absent in the UV–Vis spectrum of its parent chromophore **2B**, which was pale yellow in solution and predominately absorbed wavelengths below 400 nm. The room-temperature emission of **2A** was red-shifted by ~200 nm and showed a decreased lifetime relative to the model complex **2B**. The optical properties of the mono-terpyridyl iridium(III) complexes were less impacted by the morpholinyl substituent. Only an absorption enhancement in the range of 350–550 nm and a small red shift in the emission were observed for **1A** compared to the model chromophore **1B**. Bases on the static and time-resolved spectroscopic findings, accompanied with theoretical DFT/TD-DFT calculations, the character of the singlet and triplet excited states of **1A** was assigned as a mixed MLLCT-ILCT. The structure–property relationships discussed herein are of high importance for controlling the photophysical characteristics of [IrCl_3_(R-C_6_H_4_-terpy-κ^3^N)] and [Ir(R-C_6_H_4_-terpy-κ^3^N)_2_](PF_6_)_3_, and making further progress in the development of Ir-based luminophores.

## 7. Experimental

### 7.1. Materials and Methods

Commercially available iridium(III) chloride salt hydrate, ammonium hexafluorophosphate, 2-acetylpyridine, 4-(4-morpholinyl)benzaldehyde and benzaldehyde were used without further purification. The solvents for the syntheses and spectroscopic measurements were reagent and HPLC grade, respectively. Ligands morph-C_6_H_4_-terpy and Ph-terpy were prepared through base-mediated Kröhnke condensation from 2-acetylpyridine and two equivalents of the appropriate aldehyde (4-(4-morpholinyl)benzaldehyde for morph-C_6_H_4_-terpy and benzaldehyde for Ph-terpy, as described previously [31,71]. Elemental analyses were performed using a Vario EL Cube (Elementar) for the C, H, and N content. NMR spectra were recorded on a Bruker Avance 500 NMR spectrometer in DMSO-*d*_6_. IR spectra were acquired using a Nicolet iS5 FTIR spectrophotometer (4000–400 cm^−1^) using the KBr pellet method. X-ray diffraction data were collected at room temperature using a Gemini A Ultra diffractometer (Oxford Diffraction) with MoKα radiation (λ = 0.71073 Å), and crystallographic data for **1A**, **1B**, and **2B** were deposited with the Cambridge Crystallographic Data Center (CCDC 2359006-2359008). The UV–Vis absorption spectra were recorded on an Evolution 220 (ThermoScientific) UV–Vis spectrometer. The emission and excitation spectra along with the time-resolved TCSPC measurements at room temperature and in 77 K frozen matrix (4:1 *v*/*v*) were measured on an FLS-980 fluorescence spectrophotometer (Edinburgh Instruments). The fs TA spectra were acquired using a pump-probe transient absorption spectroscopy system (Ultrafast Systems, Helios). Theoretical calculations were performed using the GAUSSIAN-16 (Rev. C.01) program package [44] at the DFT or TD-DFT level with the PBE0 [72,73,74] functional. The basis sets used were Stuttgart Relativistic Small Core ECP with the corresponding pseudopotentials [45,75] for iridium (obtained from Basis Set Exchange Database [76]) and def2-TZVP for other elements [45,77]. A more extended experimental description is provided in the Supplementary Materials.

### 7.2. Preparation of Ir(III) Complexes

**[IrCl_3_(R-C_6_H_4_-terpy-κ^3^N)]**: A mixture of IrCl_3_·xH_2_O (0.30 g; 1 mmol) and the appropriate R-terpy ligand (1 mmol) in degassed methoxyethanol was placed in a 25 mL Teflon-lined hydrothermal synthesis autoclave reactor and heated at 120 °C for 24 h. After that, the autoclave was gradually cooled to room temperature (24 h). The resulting reddish-brown crystalline precipitate was collected by filtration; washed with acetonitrile, chloroform, and diethyl ether; and dried in air. 

**[IrCl_3_(morph-C_6_H_4_-terpy-κ^3^N)]** (**1A**): Yield: 0.48 g, 70%. **Anal. calc**. for C_25_H_22_Cl_3_IrN_4_O (693.04 g/mol): C 43.33 H 3.20, N 8.08% found: C 43.50; H 3.03; N 8.43%. **^1^H NMR** (500 MHz, DMSO-d_6_) ^1^H NMR (500 MHz, DMSO-d_6_) δ = 9.22 (dd, *J* = 5.8, 1.6 Hz, 1H), 9.04 (s, 1H), 8.92 (d, *J* = 8.1 Hz, 1H), 8.29 (td, *J* = 7.8, 1.6 Hz, 1H), 8.17 (d, *J* = 9.0 Hz, 1H), 7.96 (ddd, *J* = 7.6, 5.5, 1.4 Hz, 1H), 7.20 (d, *J* = 9.1 Hz, 1H), 3.81–3.79 (m, 2H), 3.38–3.36 (m, 2H) ppm. **^13^C NMR** (126 MHz, DMSO-d_6_) δ = 159.93, 157.44, 153.45, 151.20, 140.56, 129.62, 128.63, 125.62, 124.64, 119.59, 114.87 ppm. ^31^P NMR (202 MHz, DMSO-d_6_) δ = −133.56–(−154.64) (m, PF_6_^−^) ppm. **IR (KBr, cm^−1^)** intensity: s—strong, m—medium, w—weak: 3449 (w), 3060 (w), 2952 (w), 2844 (w), 1596 (s), 1529 (m), 1474 (w), 1446 (w), 1414 (m), 1385 (w), 1305 (w), 1265 (w), 1230 (s), 1220 (s), 1119 (m), 1051 (w), 930 (m), 883(w), 814 (w), 791 (m), 762 (w), 725 (w), 568 (w), 523 (w), 454 (w). 

**[IrCl_3_(Ph-terpy-κ^3^N)]** (**1B**): Yield: 0.110 g, 55%. **Anal. calc**. for C_21_H_15_Cl_3_IrN_3_ (607.94 g/mol): C 41.49, H 2.49, N 6.91% found: C 40.27; H 2.78; N 6.70%. **^1^H NMR** (500 MHz, DMSO-d_6_) δ = 9.22 (dd, *J* = 5.6, 1.6 Hz, 1H), 9.11 (s, 1H), 8.92 (d, *J* = 8.1 Hz, 1H), 8.31 (td, *J* = 7.8, 1.6 Hz, 1H), 8.21 (dd, *J* = 7.2, 1.4 Hz, 1H), 7.98 (ddd, *J* = 7.2, 5.6, 1.4 Hz, 1H), 7.71 (t, *J* = 7.7 Hz, 1H), 7.61 (t, *J* = 7.4 Hz, 1H) ppm. **^13^C NMR** (126 MHz, DMSO-d_6_) δ = 159.68, 159.07, 157.84, 155.95, 153.49, 153.38, 142.67, 140.73, 135.16, 130.32, 129.91, 129.72, 128.84, 128.81, 128.76, 127.46, 125.85, 123.03, 122,11, 121.56 ppm. ^31^P NMR (202 MHz, DMSO-d_6_) δ = −133.55–(−154.63) (m, PF_6_^−^) ppm. **IR (KBr, cm^−1^)** intensity: vs—very strong; s—strong, m—medium, w—weak: 3441 (m), 3047 (m), 1605 (s), 1547 (w), 1475 (m), 1451 (w), 1413 (s), 1304 (w), 1246(w), 1233 (m), 1161 (m), 1103 (w), 1051 (w), 1030 (w), 890 (m), 790 (s), 768 (s), 729 (w), 691 (m), 655 (w), 646 (w), 626 (w), 505 (w), 479 (m), 455 (w).

[**Ir(R-C_6_H_4_-terpy-κ^3^N)_2_](PF_6_)_3_**: A mixture of [IrCl_3_(R-C_6_H_4_-terpy-κ^3^N)] (0.1 mmol) and the appropriate R-C_6_H_4_-terpy ligand (0.125 mmol) in ethylene glycol (8 mL) was heated at 180 °C (oil bath) in an argon atmosphere overnight. Subsequently, a clear solution was cooled to room temperature, and a saturated aqueous solution of (NH_4_)_2_PF_6_ was added dropwise, leading to a reddish-brown (**2A**) and yellow-orange (**2B**) precipitate. After stirring for 8 h, the obtained precipitate was filtered, washed with water and diethyl ether, and dried in air. The crude products of **2A** and **2B** were purified by crystallization from the dichloromethane–methanol mixture. 

**[Ir(morph-C_6_H_4_-terpy-κ^3^N)_2_](PF_6_)_3_** (**2A**): Yield: 0.064 g, 45%. **Anal. calc**. for C_50_H_44_F_18_IrN_8_O_2_P_3_·CH_2_Cl_2_ (1500.98 g/mol): C 40.81, H 3.09, N 7.47% found: C 41.14; H 2.69; N 7.41%. **^1^H NMR** (500 MHz, DMSO-d_6_) δ = 9.49 (s, 1H), 9.21 (d, *J* = 8.4 Hz, 1H), 8.45 (d, *J* = 9.0 Hz, 1H), 8.35 (td, *J* = 7.9, 1.5 Hz, 1H), 7.95 (dd, *J* = 5.7, 1.5 Hz, 1H), 7.55 (ddd, *J* = 7.5, 5.7, 1.4 Hz, 1H), 7.31 (d, *J* = 9.1 Hz, 1H), 3.84 (t, *J* = 4.9 Hz, 2H), 3.47 (t, *J* = 4.9 Hz, 2H) ppm. **^13^C NMR** (126 MHz, DMSO-d_6_) δ = 159.04, 154.45, 153.93, 153.86, 153.61, 142.77, 129.98, 129.77, 127.41, 123.70, 121.61, 118.55, 114.61, 66.41, 47.27 ppm. **IR (KBr, cm^−1^)** intensity: vs—very strong; s—strong, m—medium, w—weak: 3422 (m), 3086 (w), 2853 (w), 1592 (s), 1527 (w), 1478 (m), 1465 (m), 1449 (m), 1419 (m), 1361 (s), 1298 (w), 1232 (s), 1213 (s), 1114 (m), 1048 (m), 1032 (m), 928 (m), 839 (vs), 785 (m), 755 (w), 645 (w), 558 (s).

**[Ir(Ph-terpy-κ^3^N)_2_](PF_6_)_3_** (**2B**): Yield: 0.081 g, 65%. **Anal. calc**. for C_42_H_30_F_18_IrN_6_P_3_·CH_2_Cl_2_ (1330.77 g/mol): C 38.81, H 2.42, N 6.32% found: C 39.19; H 2.13; N 6.35%. **^1^H NMR** (500 MHz, DMSO-d_6_ δ = 9.62 (s, 2H), 9.23 (dd, *J* = 8.2, 0.7 Hz, 2H), 8.46 (d, *J* = 7.2 Hz, 2H), 8.38 (td, *J* = 7.9, 1.4 Hz, 2H), 7.96 (dd, *J* = 5.7, 0.8 Hz, 2H), 7.85 (t, *J* = 7.6 Hz, 2H), 7.77 (t, *J* = 7.4 Hz, 1H), 7.58 (ddd, *J* = 7.5, 5.7, 1.4 Hz, 2H) ppm. **^13^C NMR** (126 MHz, DMSO-d_6_) δ = 158.69, 154.96, 154.46, 153.79, 142.97, 135.20, 132.40, 130.08, 129.96, 128.80, 127.69, 123.99, 118.55 ppm. **IR (KBr, cm^−1^)** intensity: vs—very strong; s—strong, m—medium, w—weak: 3789 (w), 3661 (w), 3432 (m), 3126 (m), 2250 (w), 1610 (s), 1556 (m), 1479 (s), 1454 (w), 1421 (s), 1244 (m), 1170 (m), 1103 (w), 1061 (w), 1034 (w), 1018(w), 977 (w), 896 (w), 838 (vs), 789 (w), 768 (s), 728 (w), 692 (m), 644(w), 558 (s), 498 (w). 

## Figures and Tables

**Table 1:** Experimental bond lengths [Å] and angles [°] for **1A**, **1B**, and **2B**.

	1A	1B	2B
Bond length [Å]			
Ir(1)–Cl(1)	2.372(1)	2.375(2)	
Ir(1)–Cl(2)	2.361(2)	2.347(2)	
Ir(1)–Cl(3)	2.363(1)	2.362(2)	
Ir(1)–N(1)	1.935(4)	1.938(5)	1.974(3)
Ir(1)–N(2)	2.046(4)	2.040(5)	2.051(3)
Ir(1)–N(3)	2.028(4)	2.045(5)	2.056(3)
Bond angle [°]			
Cl(1)–Ir(1)–Cl(2)	92.55(60)	90.03(60)	
Cl(1)–Ir(1)–Cl(3)	91.40(60)	91.29 (60)	
Cl(2)–Ir(1)–Cl(3)	175.67(50)	178.43(60)	
Cl(1)–Ir(1)–N(1)	179.39(13)	177.15(14)	
Cl(1)–Ir(1)–N(2)	98.69(12)	99.80(15)	
Cl(1)–Ir(1)–N(3)	99.39(12)	99.00(14)	
Cl(2)–Ir(1)–N(1)	86.93(12)	92.80(14)	
Cl(2)–Ir(1)–N(2)	91.46(12)	90.56(14)	
Cl(2)–Ir(1)–N(3)	87.92(12)	88.66(15)	
Cl(3)–Ir(1)–N(1)	89.13(12)	85.88(14)	
Cl(3)–Ir(1)–N(2)	89.69(12)	88.37(14)	
Cl(3)–Ir(1)–N(3)	89.70(13)	91.98(15)	
N(1)–Ir(1)–N(1a)			177.47(16)
N(1)–Ir(1)–N(2)	80.99(16)	80.53(19)	79.82 (11)
N(1)–Ir(1)–N(2a)			98.43(11)
N(1)–Ir(1)–N(3)	80.93(16)	80.73(19)	79.90(11)
N(1)–Ir(1)–N(3a)			101.88(11)
N(2)–Ir(1)–N(2a)			93.62(17)
N(2)–Ir(1)–N(3)	161.92(16)	161.18(19)	159.70(11)
N(2)–Ir(1)–N(3a)			90.27(12)
N(3)–Ir(1)–N(3a)			92.97(17)

**Table 2:** Summary of luminescence properties of **1A**–**1B** and **2A**–**2B**.

Medium	Compound	λ_ex_^a^ [nm]	λ_em_^b^ [nm]	τ_av_^c^ [μs]	Φ ^d^ [%]	Compound	λ_ex_ [nm]	λ_em_ [nm]	τ_av_ [μs]	Φ [%]
77 K	**1A**	520	639	10.90	–	**1B**	520	565	12.43	–
DMSO		440/520	670/650	1.22	3.74		440/520	600	1.75	37.80
MeCN		440/520	611/614	1.45	1.63		440/520	604	1.96	5.86
solid		530	710	0.21	4.38		525	745	0.21	2.48
77 K	**2A**	465	632	90.45	–	**2B**	370	512	40.08	–
DMSO		465/555	720/750	0.10	7.40		375/520	567/578	4.46	4.22
MeCN		465/555	765/780	0.07	0.16		375/520	522/580	6.50	10.18
solid		500	735	0.58	<0.05		500	600	10.30	4.99

^a^ λ_ex_—excitation wavelength; ^b^ λ_em_—emission wavelength; ^c^ τ_av_—average lifetime of luminescence; ^d^ Φ–quantum yield.

## Data Availability

Data are contained within the article and Supplementary Materials.

## Supplementary Materials

Crystallographic data for **1A**, **1B** and **2B** were deposited with the Cambridge Crystallographic Data Center, CCDC 2359006-2359008. Copies of this information may be obtained free of charge from the Director, CCDC, 12 Union Road, Cambridge CB2 1EZ, UK (Fax: +44 1223 336033; e-mail: deposit@ccdc.cam.ac.uk or www.ccdc.cam.ac.uk). The following supporting information can be downloaded at: https://www.mdpi.com/article/10.3390/molecules29133074/s1, Figures S1–S4: ^1^H and ^13^C NMR spectra; Figure S5: IR spectra; Table S1: Crystal data and structure refinement; Table S2: Short intra- and intermolecular contacts; Table S3: Short π•••π ring interactions; Table S4: P—F••• π-ring interactions for **2B**; Figure S6: View of 3D supramolecular structure of **1B** resulting from weak π•••π interactions; Table S5: Experimental and theoretical bond lengths and angles; Table S6: Assignment of electron excitations computed at TD-DFT/PCM/PBE0/SDD/def2TZVP level to acetonitrile UV–Vis absorptions; Table S7: Triplet energies; Figure S7: Spin density surface plots; Table S8: The absorption maxima and molar extinction coefficient values; Figure S8: The absorption spectra of **2B** recorded at 2.5 × 10^−5^ and 5 × 10^−4^ mol/dm^3^ concentrations; Figure S9: The photostability in DMSO and MeCN solutions; Figure S10: The kinetic stability; Figures S11 and S12: The emission spectra and decay curves in aerated and deaerated solutions: Figures S13 and S14: Excitation-dependent emission; Figures S15–S17: Absorbance, excitation, and emission steady-state spectra of **1**–**2** with the corresponding decay curves; Figure S18: Decay curves of **1**–**2** in solid state; Figures S19–S23: Summary of the TA analysis; Figure S24: The photodamage tests. References [31,44,45,75,76,77,78,79,80,81,82] has been cited the in the Supplementary Materials.
