# Supplementary material for: The Role of Intraligand Charge Transfer Processes in Iridium(III) Complexes with Morpholine-Decorated 4′-Phenyl-2,2′:6′,2″-terpyridine
Source: Molecules. 2024 Jun 27;29(13):3074. doi: 10.3390/molecules29133074 (PMC11243112; doi:10.3390/molecules29133074)
Supplement: Supplementary file 1 [file molecules-29-03074-s001.zip › molecules-3065792-supplementary.pdf]

SUPPORTING INFORMATION

The role of intraligand charge transfer processes in iridium(III) complexes with morpholine-decorated 4′-phenyl-2,2′:6′,2″-terpyridine

Joanna Palion-Gazda*, Aleksandra Kwiecień, Katarzyna Choroba, Mateusz Penkala, Anna Kryczka, Barbara Machura*

Institute of Chemistry, University of Silesia, 9 Szkolna Str., 40-006 Katowice, Poland

Table of contents

EXPERIMENTAL SECTION 2

Crystal structure determination and refinement 2

Computational details 2

Physical measurements 3

Photoluminescence spectra 3

Femtosecond transient absorption spectroscopy 4

NMR SPECTROSCOPY 5

Figure S1. ^{1}H (a), ^{13}C (b), NMR spectra of **1A** in DMSO-d_6. 5

Figure S2. ^{1}H (a), ^{13}C (b), NMR spectra of **1B** in DMSO-d_6. 6

Figure S3. ^{1}H (a), ^{13}C (b), ^{31}P (c) NMR spectra of **2A** in DMSO-d_6 8

Figure S4. ^{1}H (a), ^{13}C (b), ^{31}P (c) NMR spectra of **2B** in DMSO-d_6. 9

IR SPECTROSCOPY 10

Figure S5. IR spectra of complexes **1A**, **1B** (a) and **2A**, **2B** (b) 11

X-RAY ANALYSIS 12

Table S1. Crystal data and structure refinement of **1A, 1B** and **2B**. 12

Table S2. Short intra- and intermolecular contacts detected in structures **1A**, **1B** and **2B.** 12

Table S3. Short π•••π ring interactions with Cg•••Cg distances < 4Å for **1A** and **1B**. 13

Table S4. P—F•••Cg(J)(π-ring) interactions for **2B**. 13

Figure S6. View of 3D supramolecular structure of **1B** resulting from weak π•••π interactions. 14

DFT CALCULATIONS 14

Table S5. Experimental and theoretical bond lengths [Å] and angles [°] for **1A**, **1B** and **2B**. 14

Table S6. Assignment of electron excitations computed at TD-DFT/PBE0/SDD/def2TZVP level of theory with the use of the PCM model at polarities corresponding to acetonitrile to UV-Vis absorptions of **1A**, **1B**, **2A** and **2B**. 15

Table S7. Triplet energies calculated from the energy difference between the ground singlet and triplet excited states $\Delta E_{T_1-S_0}$ compared to the experimental values for complexes 1A, 1B, 2A and 2B. 19

Figure S7. Spin density surface plots for **1B** and **2B**. 20

Table S8. The absorption maxima and molar extinction coefficient values for the complexes **1–2**. 20

Figure S8. The absorption spectra of **2B** recorded in $2.5\cdot10^{-5}$ and $5\cdot10^{-4}$ mol/dm^3 concentration. 21

Figure S9. The photostability of **1A** (a), **1B** (b), **2A** (c), **2B** (d) in DMSO and MeCN solutions. 22

Figure S10. The kinetic stability of **1A** (a), **1B** (b), **2A** (c), **2B** (d) in DMSO and MeCN solutions in 2h intervals for 12h. 23

LUMINESCENCE PROPERTIES 24

Figure S11. The emission spectra and decay curves of **1A** and **1B** in an aerated and deareted solutions........ 24

Figure S12. The emission spectra and decay curves of **2A** and **2B** in an aerated and deareted solutions........ 25

Figure S13. Excitation-dependent emission spectra of **1A** and **1B** in DMSO and MeCN. 26

Figure S14. Excitation-dependent emission spectra of **2A** and **2B** in DMSO. 27

Figure S15. Absorbance, excitation and emission steady-state spectra of **1–2** in EtOH:MeOH glass matrix at 77K with the corresponding decay curves. 29

Figure S16. Absorbance, excitation and emission steady-state spectra of **1–2** in deaerated DMSO at RT with the corresponding decay curves. 30

Figure S17. Absorbance, excitation and emission steady-state spectra of **1–2** in deaerated MeCN at RT with the corresponding decay curves. 31

Figure S18. Decay curves of **1–2** in solid state. 32

FEMTOSECOND TRANSIENT ABSORPTION 33

Figure S19. Summary of the TA analysis of **1A** containing time traces at several wavelength, evolution associated spectra, residual map and transient spectra. 33

Figure S20. Summary of the TA analysis of **1B** containing time traces at several wavelength, evolution associated spectra, residual map and transient spectra. 34

Figure S21. Summary of the global lifetime analysis of **2A** containing 3 time traces at several wavelength, evolution associated spectra, residual map and transient spectra.......... 35

Figure S22. Summary of the global lifetime analysis of **2B** time traces at several wavelength, evolution associated spectra, residual map and transient spectra.......... 36

Figure S23. Comparison of the TA spectral profiles of investigated Ir(III) complexes at 3ns delay time....... 37

Figure S24. The photodamage test for **1A** and **1B** in DMSO (a) and **2A** and **2B** in MeCN (b). 38

EXPERIMENTAL SECTION

Crystal structure determination and refinement

The X-ray data for compounds **1A, 1B** and **2B** were collected using Oxford Diffraction four-circle diffractometer Gemini A Ultra with Atlas CCD detector with graphite monochromated MoKα radiation (λ = 0.71073 Å). The CrysAlis^{Pro} software [1] was used to preform diffraction data collection, cell refinement and data reduction. The structures of Ir(III) complexes were solved with the direct methods in SHELXS program and refined with the SHELXL-2014 package using least square minimization.[2] All non-hydrogen atoms were refined with anisotropic thermal parameters. Hydrogen atoms in all structures were placed in geometrically defined positions and refined with riding constrains: d(C–H) = 0.93 Å, U_{iso}(H) = 1.2 U_{eq}(C). Details of the crystallographic data collection, structural determination, and refinement for all examined complexes are summarized in Table S1.

Computational details

Theoretical DFT and TD-DFT calculations have been performed using the GAUSSIAN-16 (Rev. C.01) program package [3]. The basis sets used were Stuttgart Relativistic Small Core ECP with the corresponding pseudopotentials [4,5] for iridium (obtained from Basis Set Exchange Database) [6] and def2-TZVP for other elements [4,7]. PBE0 hybrid exchange functional has been chosen based on the previous studies on D–π–A systems [8], where hybrid-exchange functionals gave the best results. The polarizable continuum model (PCM) was used to simulate the acetonitrile solvent environment. The starting point for the geometry optimization of 1A, 1B and 2B was taken from the X-ray structures. In the case of 2A, the starting point for the geometry optimization was taken from the X-ray structure of 2B after addition of morpholine units. After optimization of the geometry, vibrational frequencies were calculated to verify the minimum on the potential energy surface. All the further calculations were obtained based on the optimized geometries. The predicted structural parameters for the ground state are within the range of error expected for DFT calculations. The calculations have been carried out using resources provided by Wroclaw Centre for Networking and Supercomputing (http://wcss.pl).

Physical measurements

All NMR spectra (^{1}H, ^{13}C, ^{31}P) were registered on Bruker Avance 500 NMR spectrometer (298 K) using DMSO-d_6 as solvent. The resonance frequency of 500 MHz, 125 MHz and 202 MHz was used for ^{1}H NMR, ^{13}C NMR and ^{31}P NMR spectra, respectively. ^{1}H and ^{13}C NMR chemical shifts where referred to solvent residual signal. Chemical shifts of ^{31}P NMR spectra were referenced to 85% phosphoric acid solution as external standard. The IR spectra were recorded using Nicolet iS5 FTIR spectrophotometer (4000–400 cm^{-1}) in the form of KBr pellets. The UV-Vis spectra were measured using ThermoScientific Evolution 220 spectrometer in DMSO and acetonitrile solutions. In the kinetic stability studies, UV-Vis spectra of investigated compounds in acetonitrile and chloroform solutions were measured once every two hours for 12h. The photostability of all complexes in DMSO and acetonitrile solutions were verified by measurements of the series of UV-Vis spectra, each after 20 minutes of irradiation of the sample with xenon light source at low-energy excitation wavelength.

Photoluminescence spectra

Steady-state luminescence spectra of argon-saturated samples in acetonitrile and DMSO solutions were measured on FLS-980 fluorescence spectrophotometer (Edinburgh Instruments). Spectrophotometer FLS-980 is equipped with a 450 W Xe lamp and high-gain photomultiplier PMT + 500nm (Hamamatsu, R928P) detector. Measurements were carried out for both deaerated and aerated samples. Deaerated samples were prepare using 1h of argon-bubbling. The emission spectra at 77 K were registered in an ethanol:methanol (4:1 v/v) matrix frozen with liquid nitrogen. The quantum yields of luminescence were determined by absolute method using the integrating sphere with solvent or Spectralon® reflectance standard (for powdered samples) as blanks. The FLS-980 software was used to perform the emission correction and calculation of the quantum yield. The PL lifetime measurements were carried out with a time correlated single photon counting (TCSPC) method using the diodes

(EPLED 470 nm, EPLED 515 nm) with picosecond pulse period as excitation light sources and PMT (Hamamatsu, R928P, Japan) as a detector. The IRF was designates using ludox solution. Obtained decay curves were fitted using deconvolution fit analysis with one or two exponential terms. Long-lived phosphorescence lifetimes were measured with use of 60 W microsecond Xe flash lamp with 10 Hz or 0.1 Hz repetition rate, and the calculations of the lifetimes were performed via FLS-980 software.

Femtosecond transient absorption spectroscopy

The fsTA spectra were measured using a pump–probe transient absorption spectroscopy system (Ultrafast Systems, Helios), which was specified previously.[9,10] The samples of **1A** and **1B** were prepared in DMSO, while **2A** and **2B** in acetonitrile, with absorbance of the first absorption band equal 0.5 (which correspond to concentrations in the range 2.5×10^{-4} – 5×10^{-5} mol/dm^3), and placed in a 2 mm path length quartz cells equipped magnetic stirring. The samples were excited with 355 nm for all compounds. Transient absorption data were prepared using the Surface Xplorer (Ultrafast Systems) software and then analysed with use of Optimus^{TM} software. Correction of background, scattered light subtraction were performed routinely prior to the analysis. Also, coherent artefact analysis provided necessary information about IRF (IRF FWHM was estimated as about 200 fs) and allowed to the corrections of the probe chirp. In the ΔA vs λ spectra positive bands due to excited-state absorption (ESA), whereas the negative are ground state bleaching (GSB) bands. The global analysis was performed using linear unidirectional sequential model implemented in Optimus^{TM} software[11]. The analysis allowed to conduct the deconvolution of the transient spectra into species-associated spectra (SAS) and provides the decay-associated spectra (DAS) as a linear combination of the SAS of the compartments. During the femtosecond experiments photodamage tests were conduct (Figure S24).

NMR SPECTROSCOPY

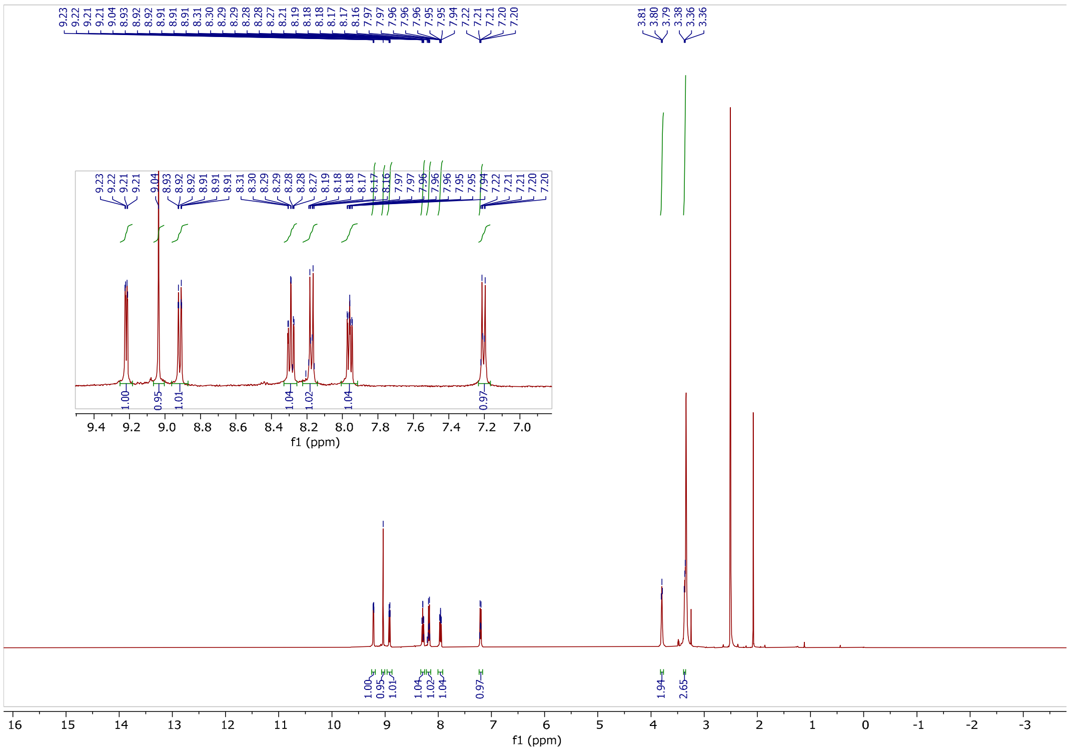

(a)

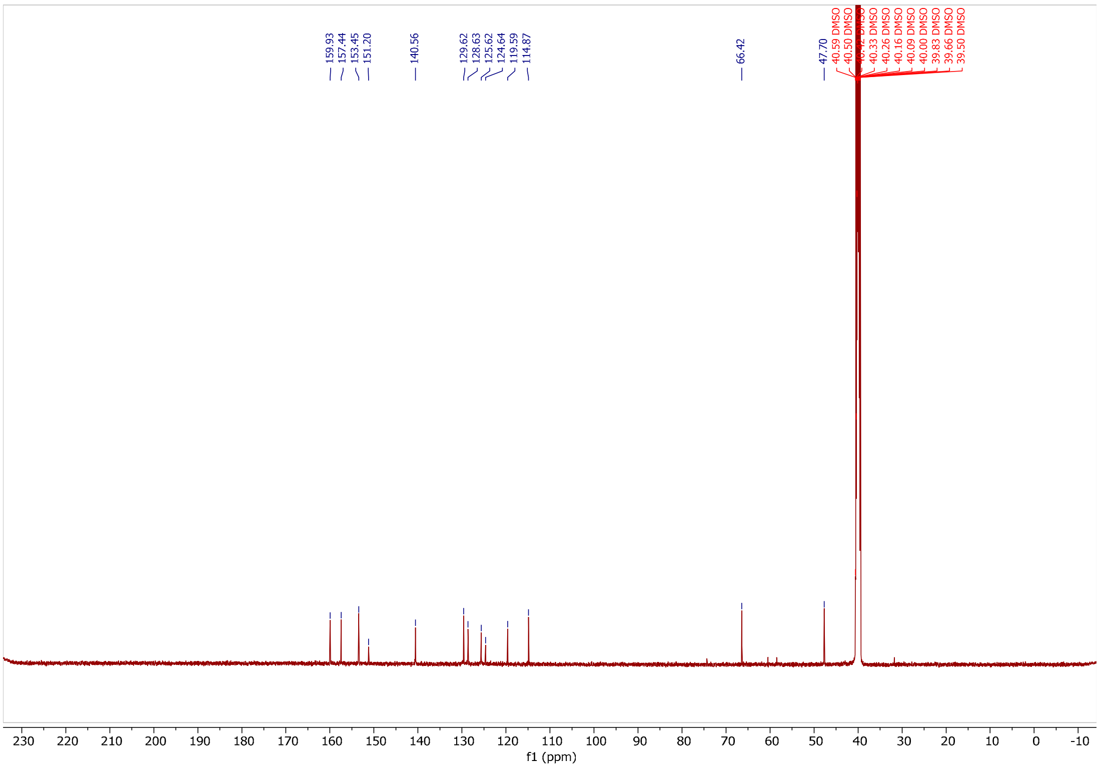

(b)

Figure S1. ^{1}H (a), ^{13}C (b), NMR spectra of **1A** in DMSO-d6.

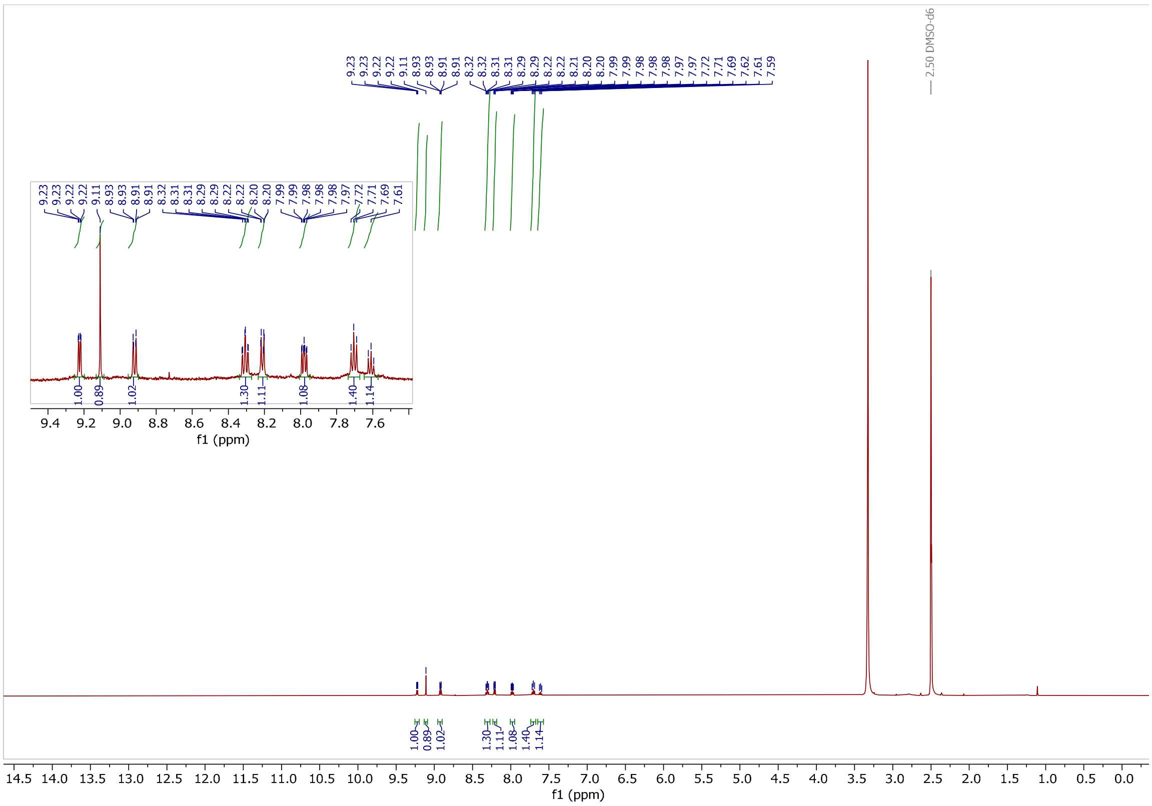

(a)

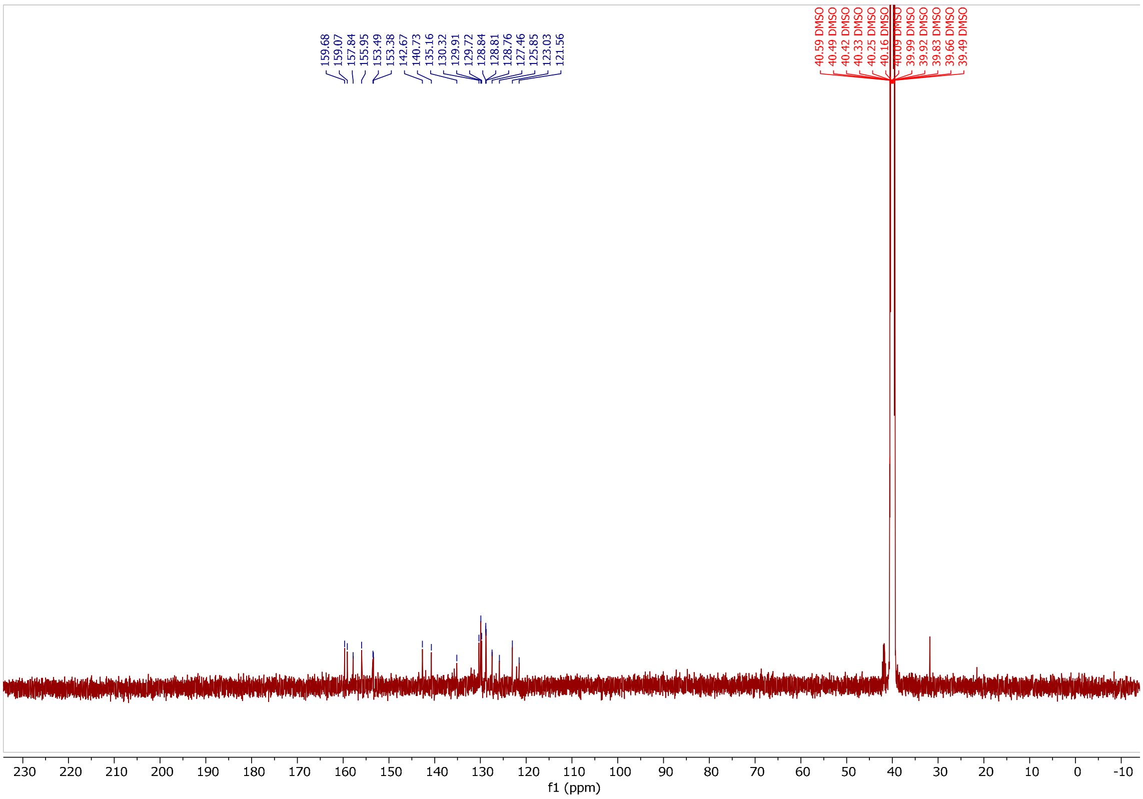

(b)

Figure S2. ^{1}H (a), ^{13}C (b), NMR spectra of **1B** in DMSO-d$_6$.

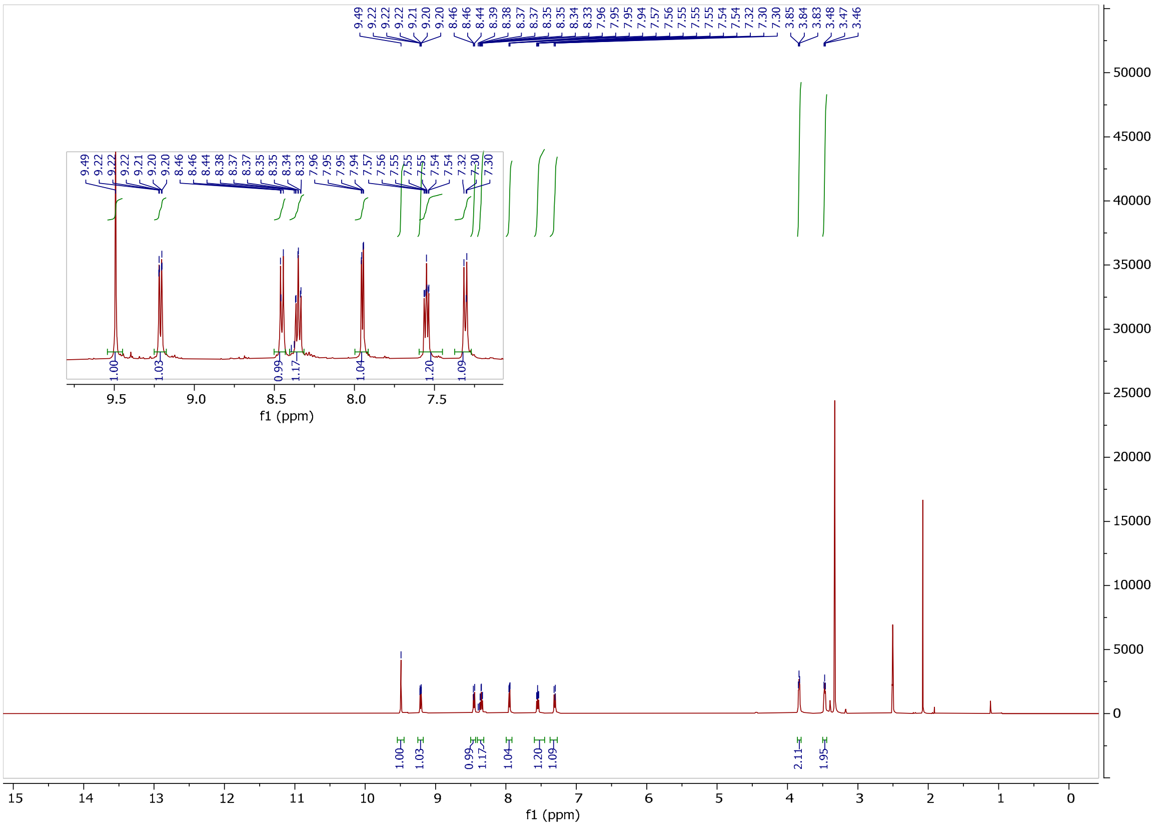

(a)

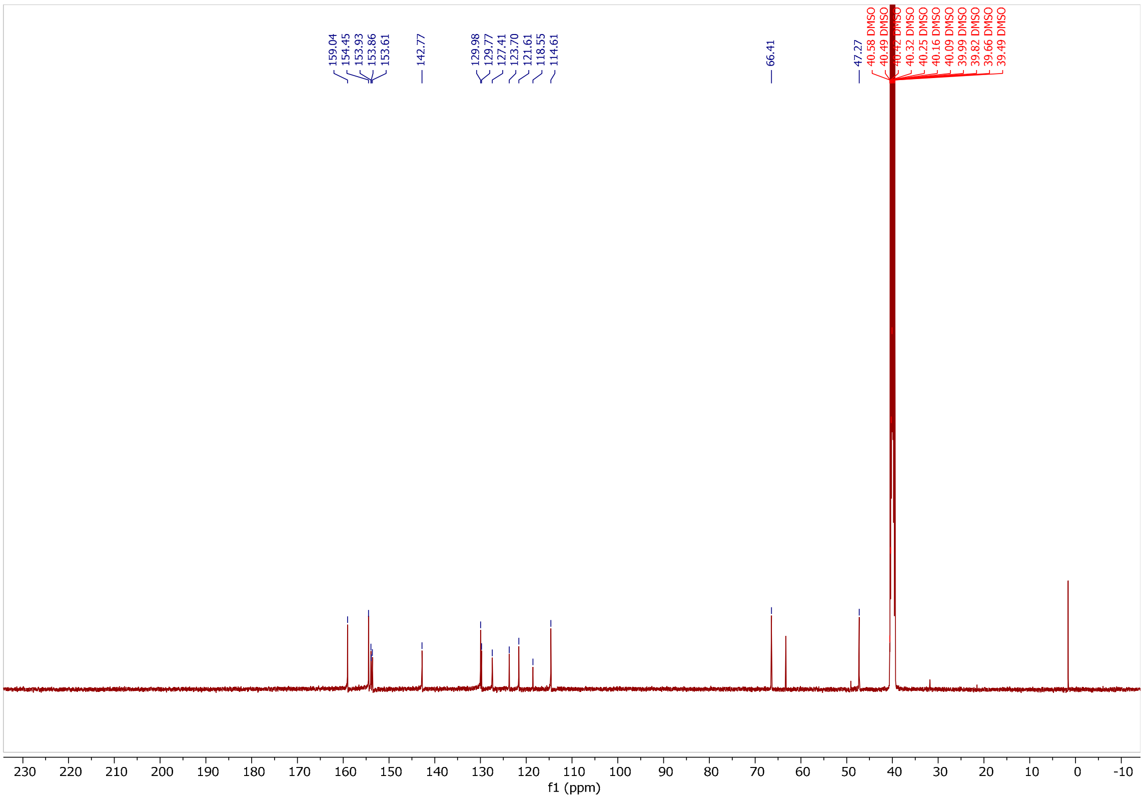

(b)

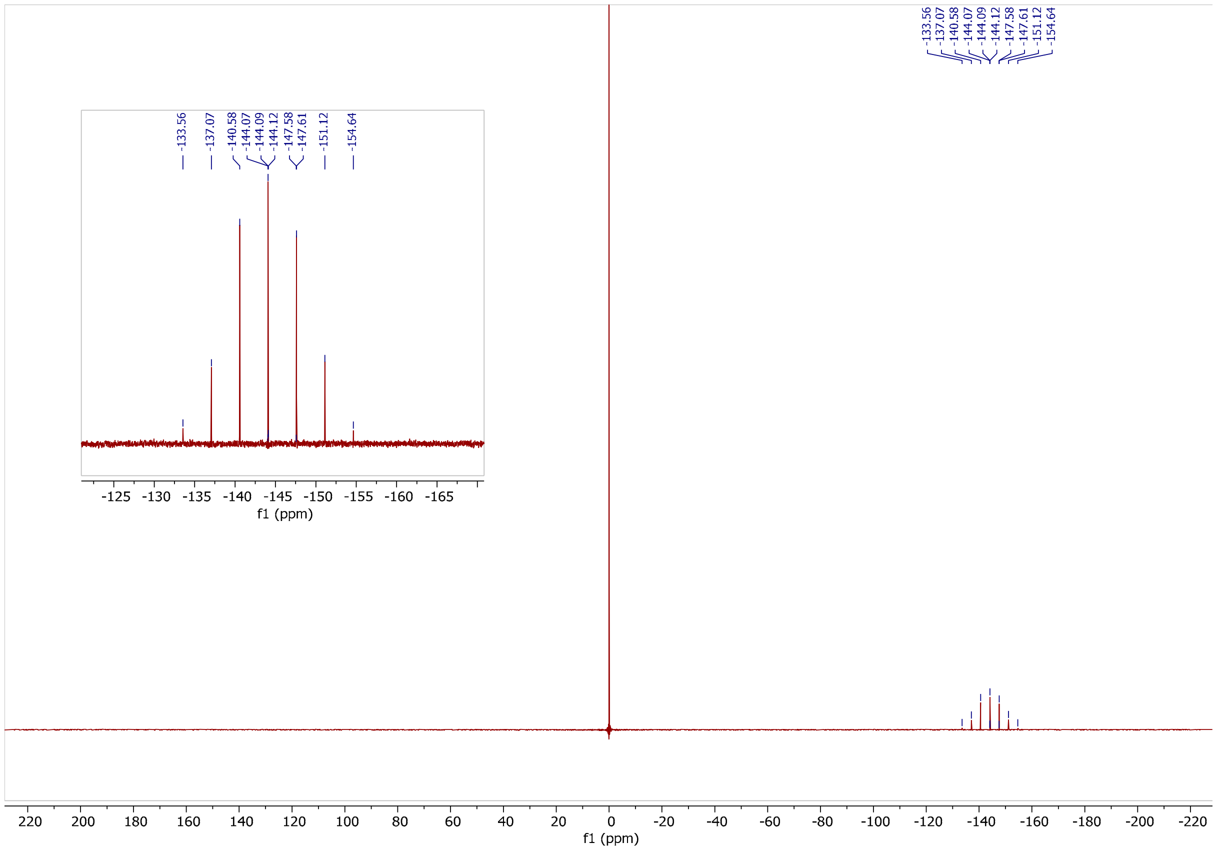

(c)

Figure S3. **^{1}H (a), ^{13}C (b), ^{31}P (c) NMR spectra of 2A in DMSO-d_6.**

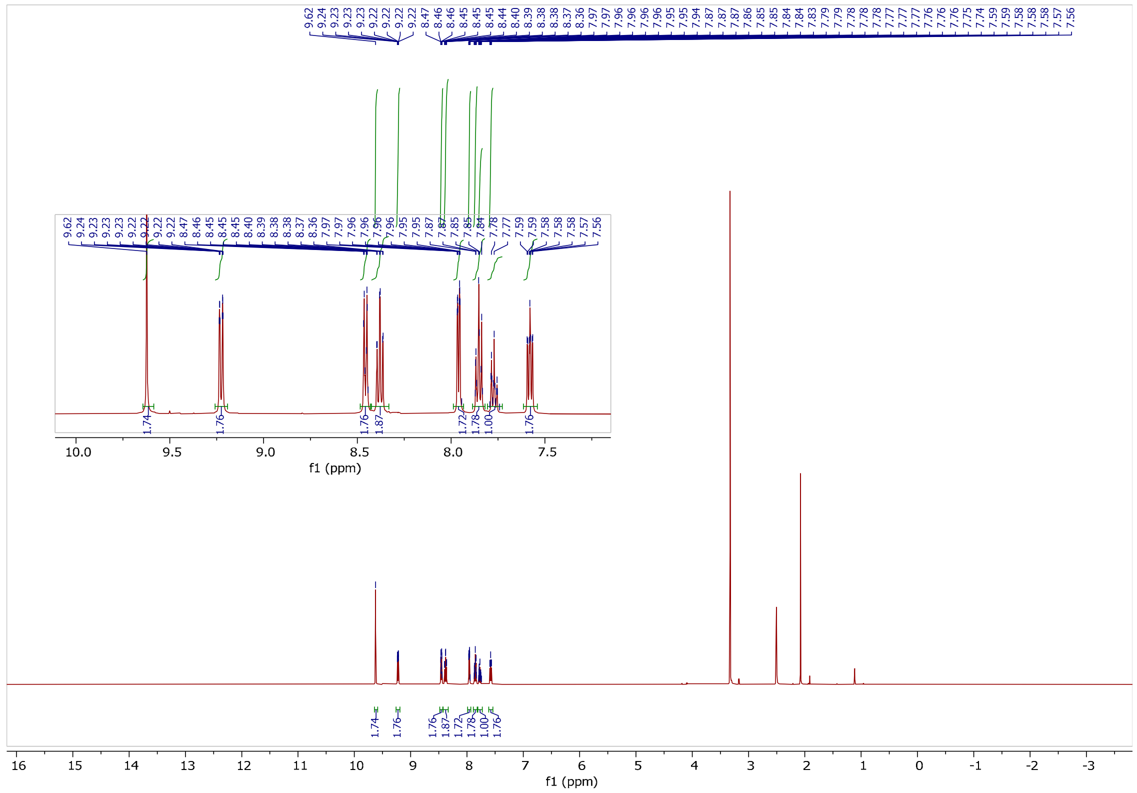

(a)

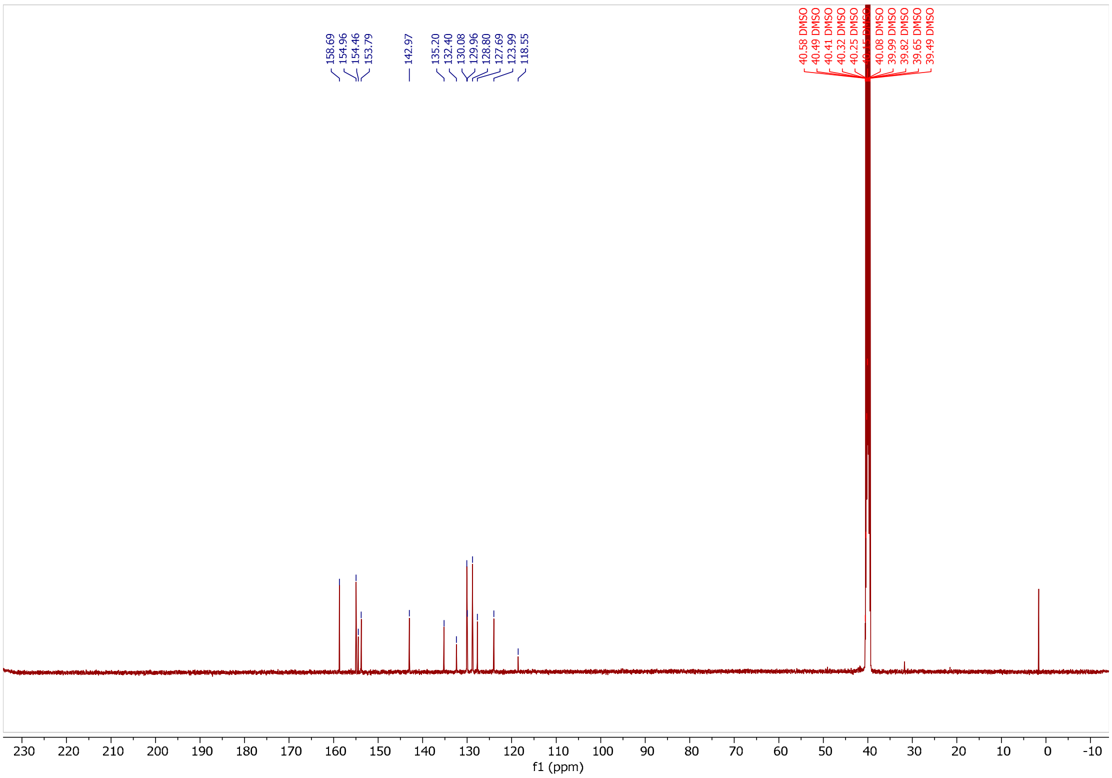

(b)

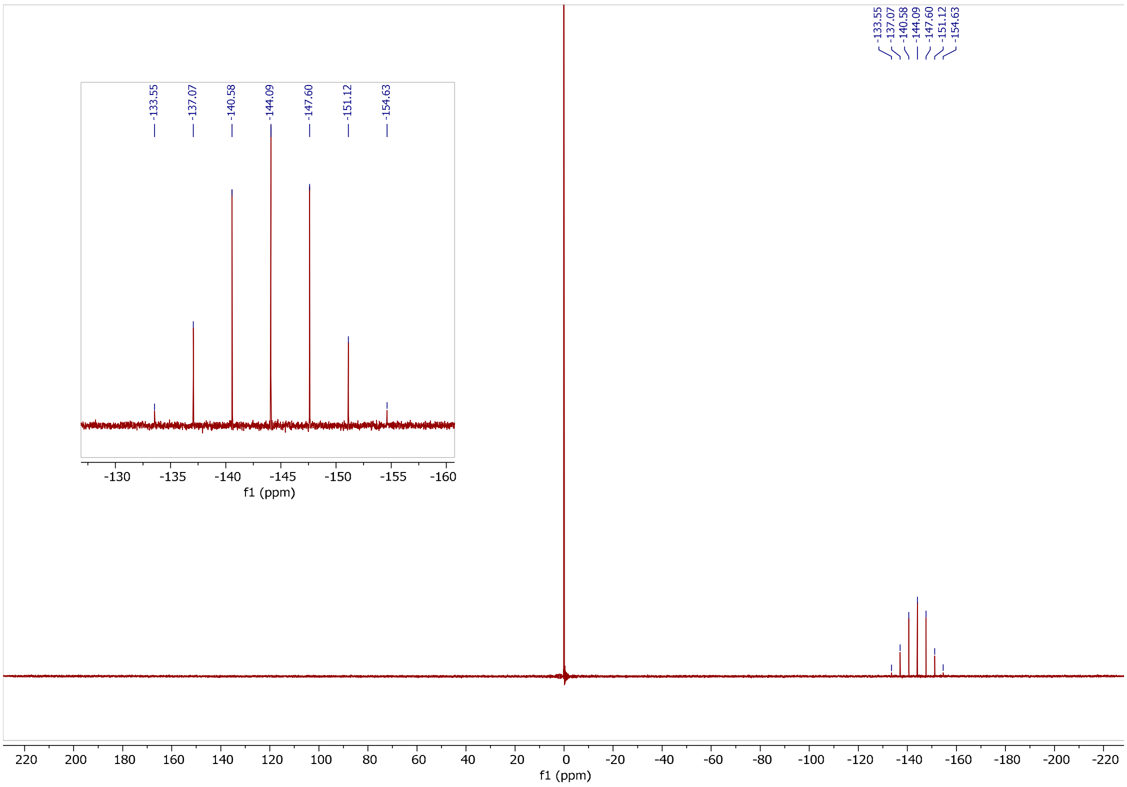

(c)

Figure S4. ^{1}H (a), ^{13}C (b), ^{31}P (c) NMR spectra of **2B** in DMSO-d_6.

IR SPECTROSCOPY

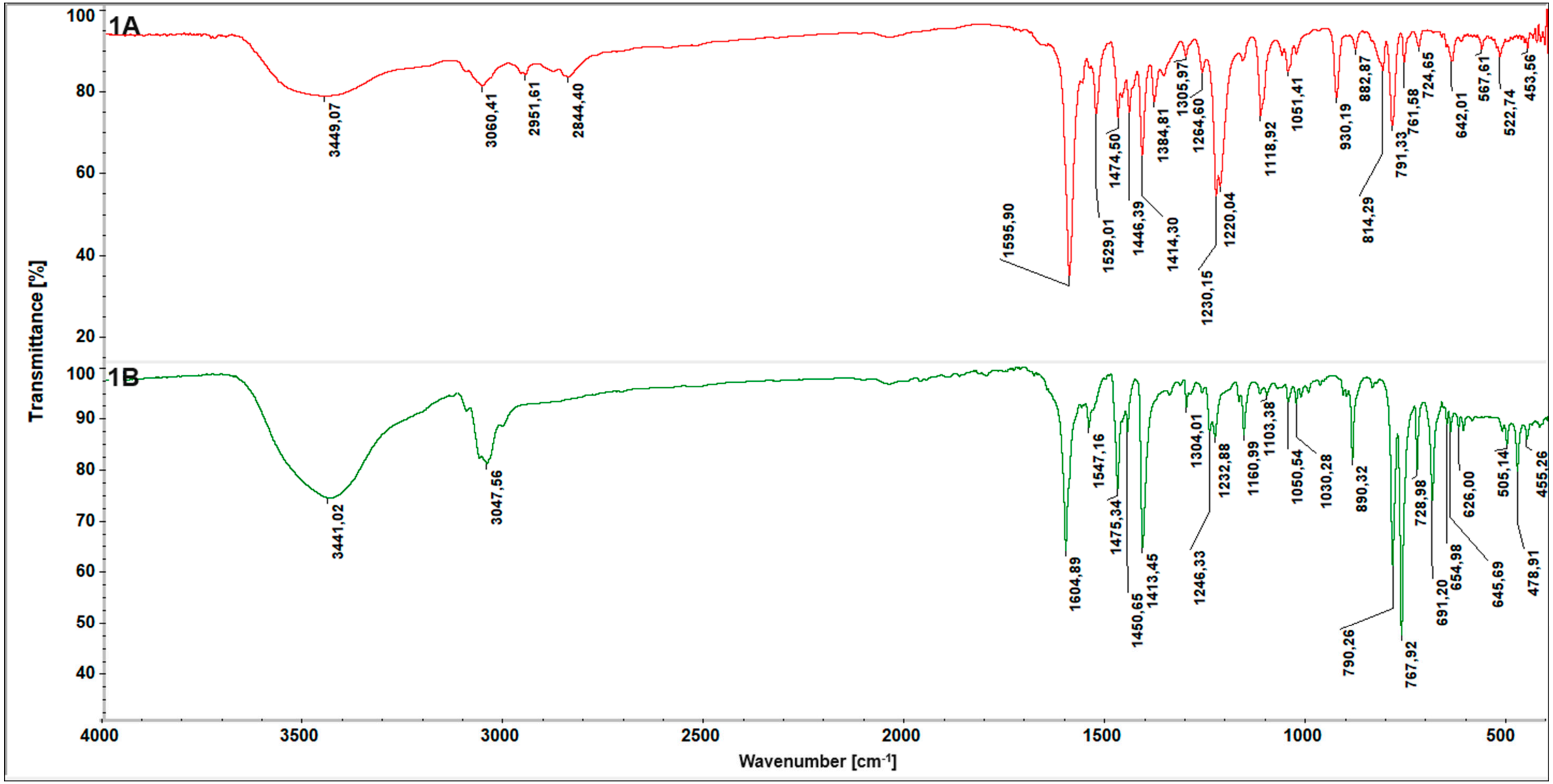

(a)

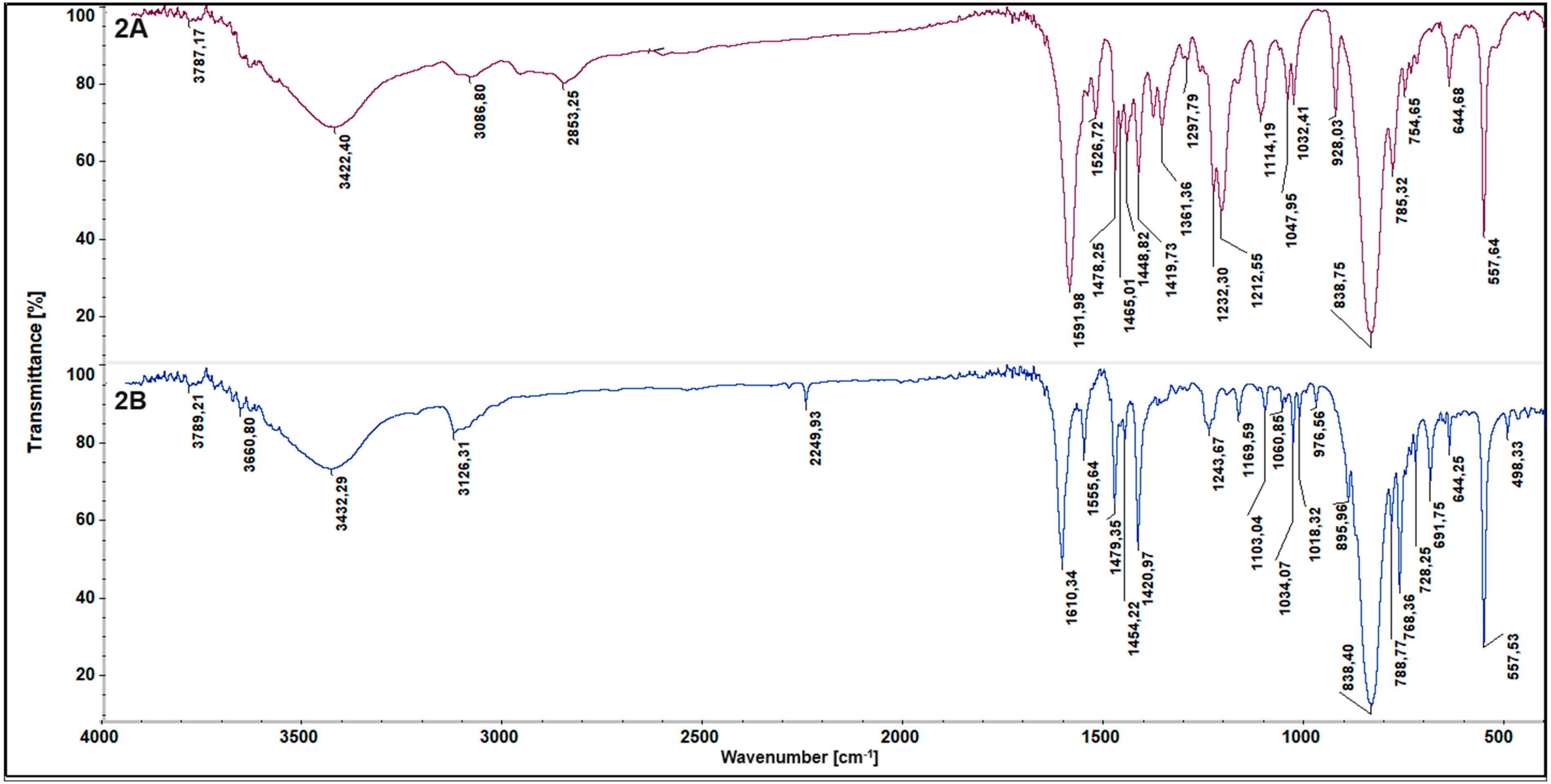

(b)

Figure S5. IR spectra of complexes **1A, 1B** (a) and **2A, 2B** (b).

X-RAY ANALYSIS

Table S1. Crystal data and structure refinement of **1A**, **1B** and **2B**.

	**1A**	**1B**	**2B**
Empirical formula	$C_{25}H_{22}Cl_3IrN_4O$	$C_{21}H_{15}Cl_3IrN_3$	$C_{46}H_{36}F_{18}IrN_8P_3$
Formula weight	693.01	607.91	1327.94
Temperature [K]	293.0(2)	293.0(2)	293.0(2)
Wavelength [Å]	0.71073	0.71073	0.71073
Crystal system	*monoclinic*	*monoclinic*	*monoclinic*
Space group	*C*2/*c*	*P*2$_1$/*c*	*C*2/*c*
Unit cell dimensions [Å, °]	a = 18.6044(8)	a = 10.2968(5)	a = 14.7880(5)
	b = 13.1121(5)	b = 13.9918(7)	b = 16.3066(6)
	c = 20.2140(8)	c = 13.6761(8)	c = 21.7790(8)
	β = 90.534(4)	β = 98.539(5)	β = 105.227(4)
Volume [Å ^{3}]	4930.8(3)	1948.48(18)	5067.5(3)
Z	8	4	4
Density (calculated) [g/cm^3]	1.867	2.072	1.741
Absorption coefficient [mm^{-1}]	5.767	7.276	2.839
F(000)	2688.0	1160.0	2608.0
Crystal size [mm]	0.102×0.076×0.033	0.291×0.204×0.078	0.237×0.166×0.091
θ range for data collection [°]	3.56 to 29.31	3.53 to 29.44	3.73 to 29.44
Index ranges	$-22 \le h \le 25$	$-14 \le h \le 13$	$-16 \le h \le 15$
	$-13 \le k \le 17$	$-18 \le k \le 18$	$-18 \le k \le 17$
	$-25 \le l \le 27$	$-11 \le l \le 18$	$-33 \le l \le 33$
Reflections collected	13689	11042	12461
Independent reflections	5842 (R_{int} = 0.0324)	4628 (R_{int} = 0.0529)	6043 (R_{int} = 0.0304)
Completeness to 2θ=50° [%]	86.6	85.7	85.9
Max. and min. transmission	0.459 and 1.000	0.213 and 1.000	0.651 and 1.000
Data / restraints / parameters	5842/0/307	4628/0/253	6043/0/345
Goodness-of-fit on F^2	1.103	1.050	1.089
Final R indices [I>2σ(I)]	R_1 = 0.0379	R_1 = 0.0408	R_1 = 0.0337
	wR_2 = 0.0749	wR_2 = 0.0820	wR_2 = 0.0756
R indices (all data)	R_1 = 0.0594	R_1 = 0.0623	R_1 = 0.0412
	wR_2 = 0.081	wR_2 = 0.0941	wR_2 = 0.0798
Largest diff. peak and hole [eÅ^{-3}]	1.04/–0.67	1.40/–1.37	0.89/–0.54

Table S2. Short intra- and intermolecular contacts detected in structures **1A**, **1B** and **2B**.

D	A[a]	D—H [Å]	H•••A [Å]	D•••A [Å]	D—H•••A [°]
			**1A**		
C(4)	Cl(2a)	0.93	2.75	3.625(6)	156.0
C(7)	Cl(3b)	0.93	2.73	3.598(6)	155.0
C(12)	Cl(2a)	0.93	2.65	3.508(6)	154.0
C(13)	O(1c)	0.93	2.44	3.361(9)	170.0
C(14)	Cl(3d)	0.93	2.75	3.658(6)	167.0
			**1B**		
C(2)	Cl(2e)	0.93	2.72	3.641(6)	173.0
C(7)	Cl(2e)	0.93	2.75	3.675(6)	178.0
C(8)	Cl(2f)	0.93	2.66	3.385(7)	135.0
C(14)	Cl(3g)	0.93	2.80	3.437(7)	127.0
C(21)	Cl(3h)	0.93	2.81	3.727(7)	168.0
			**2B**		

C(1)	N(4i)	0.93	2.50	3.294(8)	143.0
C(9)	F(8j)	0.93	2.40	3.331(6)	174.0
C(12)	F(8j)	0.93	2.53	3.460(6)	174.0
C(21)	F(8j)	0.93	2.53	3.323(6)	143.0

[a]Symmetry code: (a) = 1/2–*x*, 1/2+*y*, 3/2–*z*; (b) = 1–*x*, *y*, 3/2–*z*; (c) = –1/2+*x*, 3/2–*y*, –1/2+*z*; (d) = 1/2–*x*, –1/2+*y*, 3/2–*z*; (e) = 1–*x*, 1/2+*y*, 1/2–*z*; (f) = *x*, 1/2–*y*, –1/2+*z*; (g) = 2–*x*, –*y*, 1–*z*; (h) = *x*, 1/2–*y*, 1/2+*z*; (i) = 1+*x*, *y*, *z*; (j) = –1/2+*x*, –1/2+*y*, *z*.

Table S3. Short π•••π ring interactions with Cg•••Cg distances < 4Å for **1A** and **1B.**

Cg(I)•••Cg(J)[g]	Cg(I)•••Cg(J) [Å]	α[°][b]	β[°][c]	γ [°][d]	Cg(I)-Perp [Å][e]	Cg(J)-Perp [Å][f]
**1A**						
Cg(1) •••Cg(1b)	3.953(3)	3	25.37	25.37	3.572(2)	3.572(2)
Cg(1) •••Cg(2d)	3.878(3)	4.3(3)	23.07	26.38	–3.568(2)	–3.475(2)
**1B**						
Cg(1) •••Cg(3f)	3.989(3)	3.5(3)	30.70	27.56	–3.536(2)	3.431(2)
Cg(1) •••Cg(4g)	3.865(3)	17.1(3)	34.78	17.89	–3.678(2)	–3.174(3)

[a] Symmetry code: (b) = 1–*x*, *y*, 3/2–*z*; (d) = 1/2–*x*, –1/2+*y*, 3/2–*z*; (f) = *x*, 1/2–*y*, –1/2+*z*; (g) = 1–*x*, –1/2–*y*, 1/2+*z*.
[b]α = dihedral angle between *Cg*(I) and *Cg*(J);
[c] β = angle *Cg*(I)→*Cg*(J) vector and normal to ring I;
[d] γ = angle between the *Cg*(I) →*Cg*(J) vector and the normal to plane J;
[e] *Cg*(I)-Perp = Perpendicular distance of *Cg*(I) on ring J;
[f] *Cg*(J)-Perp = perpendicular distance of *Cg*(J) on ring I
[g] Cg1 is the centroid of the N(2)/C(6)/C(7)/C(8)/C(9)/C(10) set of atoms; Cg2 is the centroid of the N(3)/C(11)/C(12)/C(13)/C(14)/C(15) set of atoms. Cg3 is the centroid of the N(1)/C(1)/C(2)/C(3)/C(4)/C(5) set of atoms. Cg4 is the centroid of the C(16)/C(17)/C(18)/C(19)/C(20)/C(21) set of atoms.

Table S4. P—F•••Cg(J)(π-ring) interactions for **2B.**

P–F(I)•••Cg(J)[c]	F(I)•••Cg(J) [Å]	F-Perp [Å]	γ [°][b]	P–F(I)•••Cg(J) [°]
P(1)–F(1)•••Cg(3)	3.712(5)	–3.298	27.34	100.3(2)
P(1)–F(2)•••Cg(3)	3.093(4)	–3.020	12.44	130.0(2)
P(2)–F(7)•••Cg(1)	3.237(5)	3.185	10.34	113.6(2)
P(2)–F(7)•••Cg(1k)	3.710(5)	3.267	28.30	93.6(2)
P(2)–F(8)•••Cg(1)	3.452 (5)	3.001	29.62	103.7(2)

[a] Symmetry codes: (k) = = 2–*x*, *y*, 3/2–*z*;
[b] γ = angle X(I)→Cg(J) vector and normal to plane J.
[c] Cg1 is the centroid of the N(2)/C(6)/C(7)/C(8)/C(9)/C(10) set of atoms; Cg3 is the centroid of the N(1)/C(1)/C(2)/C(3)/C(4)/C(5) set of atoms.

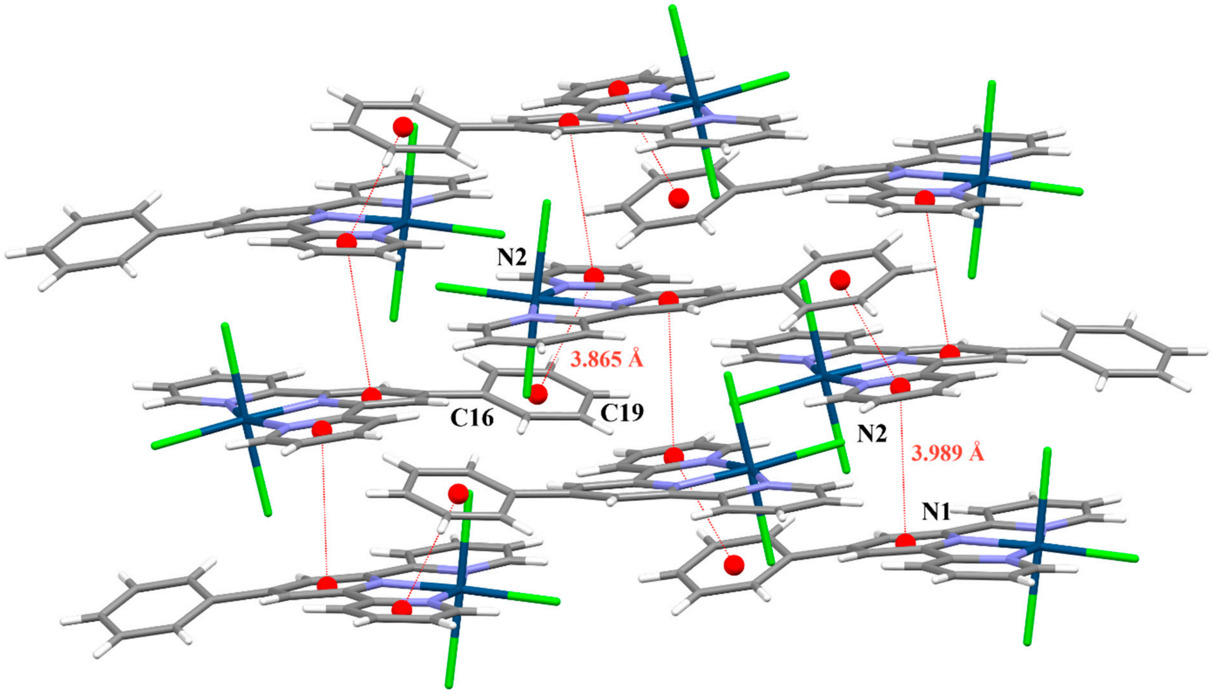

Figure S6. View of 3D supramolecular structure of **1B** resulting from weak π•••π interactions (red dashed line).

DFT CALCULATIONS

Table S5. Experimental and theoretical bond lengths [Å] and angles [°] for **1A**, **1B** and **2B**.

	1A experimental	1A theoretical	1B experimental	1B theoretical	2B experimental	2B theoretical
**Bond lengths [Å]**						
Ir(1)–Cl(1)	2.3721(14)	2.4015	2.3746(16)	2.3988		
Ir(1)–Cl(2)	2.3614(15)	2.3678	2.3472(17)	2.3671		
Ir(1)–Cl(3)	2.3627(15)	2.3677	2.3622(16)	2.3671		
Ir(1)–N(1)	1.935(4)	1.9431	1.938(5)	1.9427	1.976(3)	1.9837
Ir(1)–N(2)	2.046(4)	2.0496	2.040(5)	2.0493	2.053(3)	2.0702
Ir(1)–N(3)	2.028(4)	2.0497	2.045(5)	2.0493	2.055(3)	2.0703
**Bond angles [°]**						
Cl(1)–Ir(1)–Cl(2)	92.55(6)	90.31	90.03(6)	90.27		
Cl(1)–Ir(1)–Cl(3)	91.40(6)	90.30	91.29 (6)	90.27		
Cl(2)–Ir(1)–Cl(3)	175.67(5)	179.39	178.43(6)	179.47		
Cl(1)–Ir(1)–N(1)	179.39(13)	179.91	177.15(14)	179.97		
Cl(1)–Ir(1)–N(2)	98.69(12)	99.46	99.80(15)	99.45		
Cl(1)–Ir(1)–N(3)	99.39(12)	99.55	99.00(14)	99.43		
Cl(2)–Ir(1)–N(1)	86.93(12)	89.78	92.80(14)	89.70		
Cl(2)–Ir(1)–N(2)	91.46(12)	90.27	90.56(14)	90.33		
Cl(2)–Ir(1)–N(3)	87.92(12)	89.63	88.66(15)	89.58		
Cl(3)–Ir(1)–N(1)	89.13(12)	89.62	85.88(14)	89.76		
Cl(3)–Ir(1)–N(2)	89.69(12)	89.66	88.37(14)	89.57		
Cl(3)–Ir(1)–N(3)	89.70(13)	90.24	91.98(15)	90.34		
N(1)–Ir(1)–N(1a)					177.50(18)	180.00
N(1)–Ir(1)–N(2)	80.99(16)	80.50	80.53(19)	80.56	79.79(13)	79.60
N(1)–Ir(1)–N(2a)					98.47(13)	100.40
N(1)–Ir(1)–N(3)	80.93(16)	80.49	80.73(19)	80.56	79.84(12)	79.61

N(1)–Ir(1)–N(3a)					101.91(13)	100.39
N(2)–Ir(1)–N(2a)					93.72(19)	91.86
N(2)–Ir(1)–N(3)	161.92(16)	160.99	161.18(19)	161.12	159.62(13)	159.21
N(2)–Ir(1)–N(3a)					90.22(13)	91.87
N(3)–Ir(1)–N(3a)					93.02(19)	91.86

Table S6. Assignment of electron excitations computed at TD-DFT/PBE0/SDD/def2TZVP level of theory with the use of the PCM model at polarities corresponding to acetonitrile to UV-Vis absorptions of **1A, 1B, 2A** and **2B.**

1A

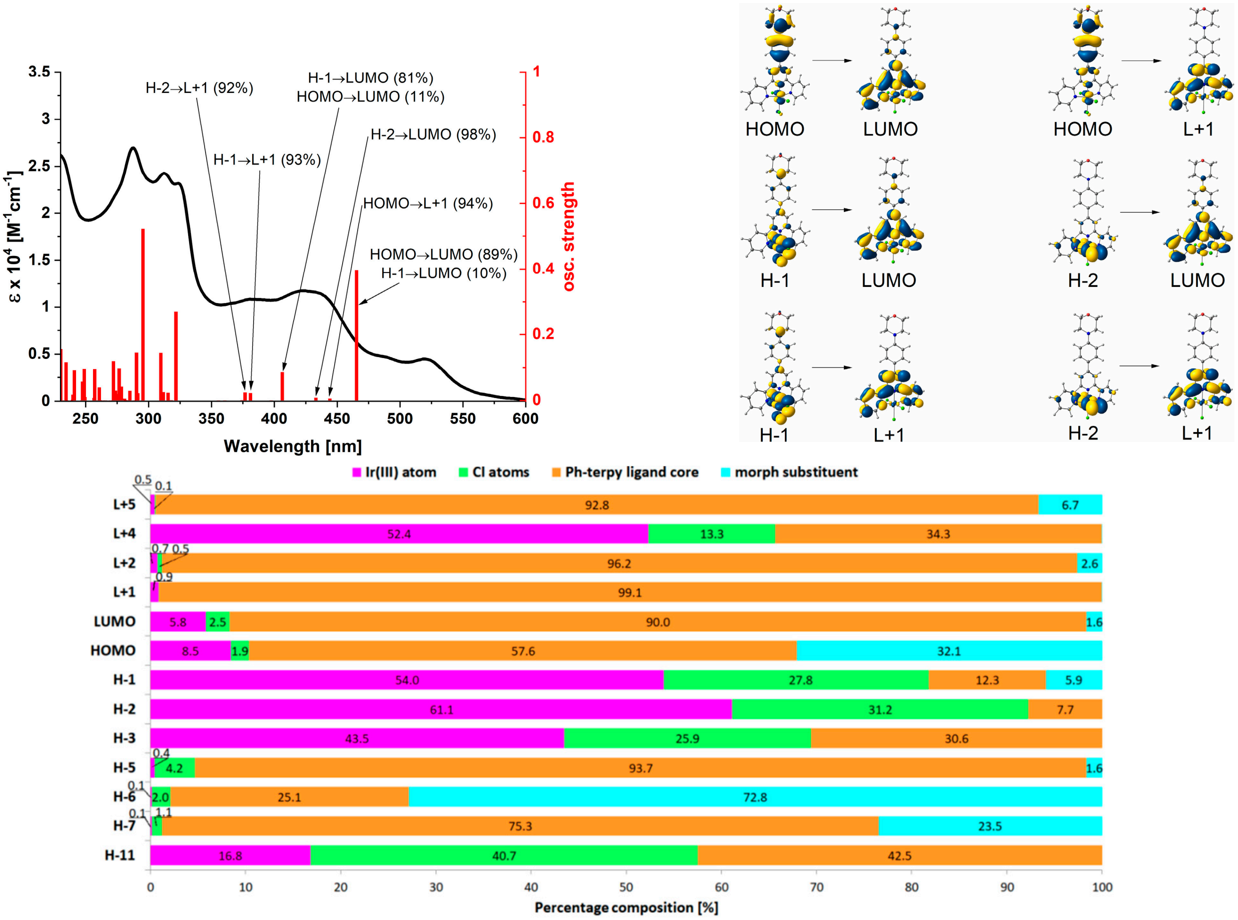

Exp. absorption λ; nm (10^4 ε; $M^{-1}cm^{-1}$)	Calculated transitions					
	Major contribution (%)	Character	E [eV]	λ [nm]	Oscillator strength	transition No.
488.6 (0.45)	HOMO→LUMO (89%)<br>H-1→LUMO (10%)	ILCT/IL<br>MLLCT	2.66	465.51	0.3978	S1
427.5 (1.11)	HOMO→L+1 (94%)	ILCT/IL	2.79	444.24	0.0057	S2
	H-2→LUMO (98%)	MLLCT	2.86	433.11	0.0081	S3
	H-1→LUMO (81%)<br>HOMO→LUMO (11%)	MLLCT<br>ILCT/IL<br>MLLCT	3.05	406.42	0.0849	S4
378.3 (1.03)	H-1→L+1 (93%)	MLLCT<br>ILCT/IL	3.25	381.04	0.0218	S6
	H-2→L+1 (92%)	MLLCT<br>MLLCT	3.29	376.78	0.024	S7
323.7 (2.28)	H-3→L+1 (97%)	MLLCT/IL	3.85	321.80	0.2697	S11
313.1 (2.36)	H-5→LUMO (66%)	IL	4.00	309.80	0.1444	S15

	H-3→L+4 (13%)	MLLCT/IL				
288.8 (2.61)	HOMO→L+5 (72%)<br>H-1→L+2 (19%)	ILCT/IL<br>MLLCT	4.19	295.63	0.5213	S16
	H-2→L+2 (70%)	MLLCT	4.27	290.44	0.145	S19
	H-5→L+1 (53%)<br>H-7→L+1 (27%)	IL<br>IL	4.48	276.65	0.0972	S29
	H-7→L+1 (33%)<br>H-5→L+1 (29%)<br>H-6→L+1 (13%)	IL<br>IL<br>ILCT/IL	4.56	272.14	0.1193	S33
	H-11→LUMO (84%)	IL/LLCT	4.82	257.12	0.0953	S40

1B

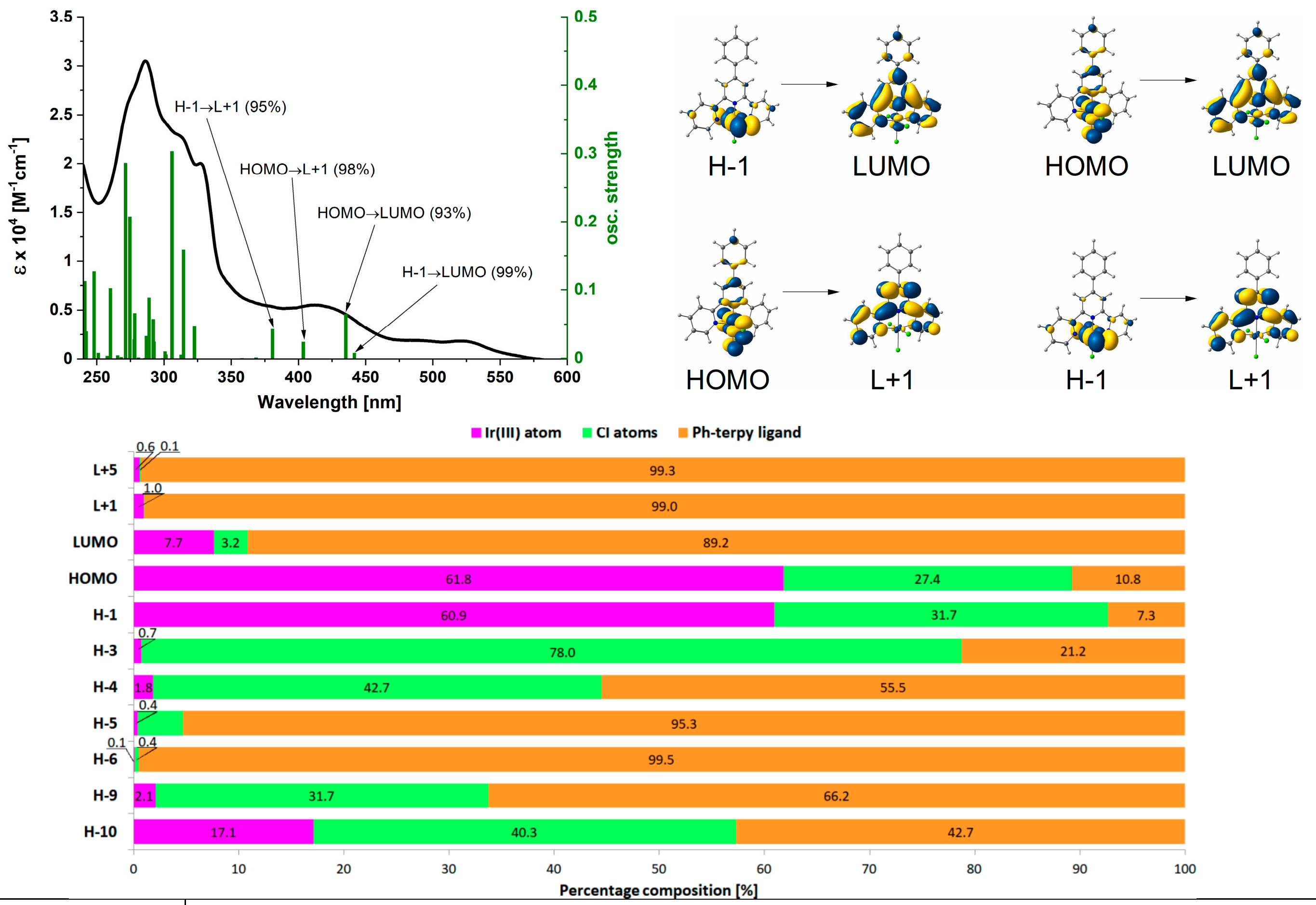

Exp. absorption λ; nm (10^4 ε; $M^{-1}cm^{-1}$)	Calculated transitions					
	Major contribution (%)	Character	E [eV]	λ [nm]	Oscillator strength	transition No.
489.4 (0.17)	H-1→LUMO (99%)	MLLCT	2.81	441.68	0.0078	S1
418.7 (0.54)	HOMO→LUMO (93%)	MLLCT	2.85	435.29	0.0649	S2
	HOMO→L+1 (98%)	MLLCT	3.07	403.70	0.0241	S4
	H-1→L+1 (95%)	MLLCT	3.26	380.78	0.043	S5
328.3 (1.97)	H-3→LUMO (92%)	LLCT/IL	3.84	322.68	0.0472	S9
312.5 (2.29)	H-5→LUMO (76%)<br>H-3→L+1 (10%)	IL<br>LLCT/IL	3.94	314.51	0.1587	S10
285.3 (3.05)	H-4→LUMO (85%)	IL/LLCT	4.05	305.97	0.3035	S12
	H-5→L+1 (65%)<br>H-6→L+1 (19%)	IL<br>IL	4.51	274.66	0.2074	S26
272.6 (2.63)	HOMO→L+5 (86%)	MLLCT	4.57	271.48	0.2862	S28
	H-10→LUMO (86%)	IL/LLCT	4.76	260.22	0.1022	S33
	H-9→L+1 (85%)	IL/LLCT	5.00	248.05	0.1274	S37

2A

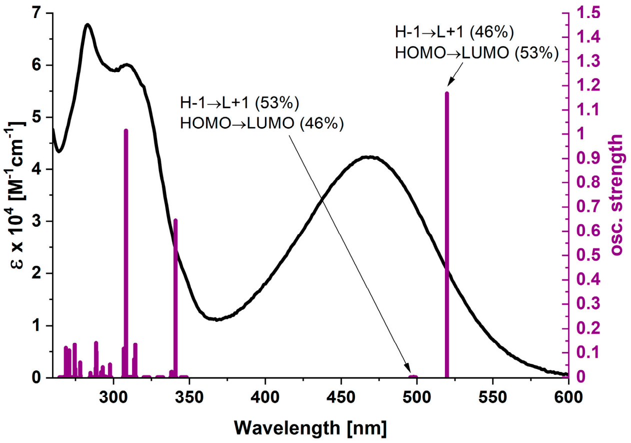

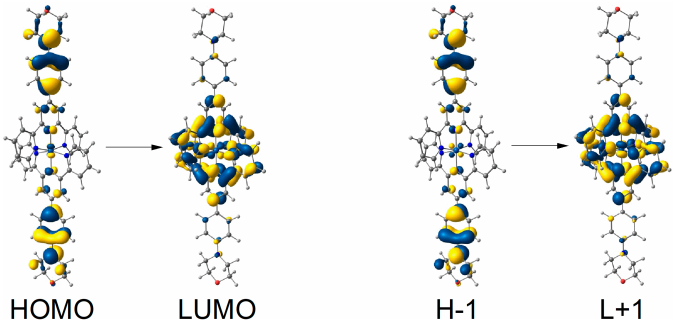

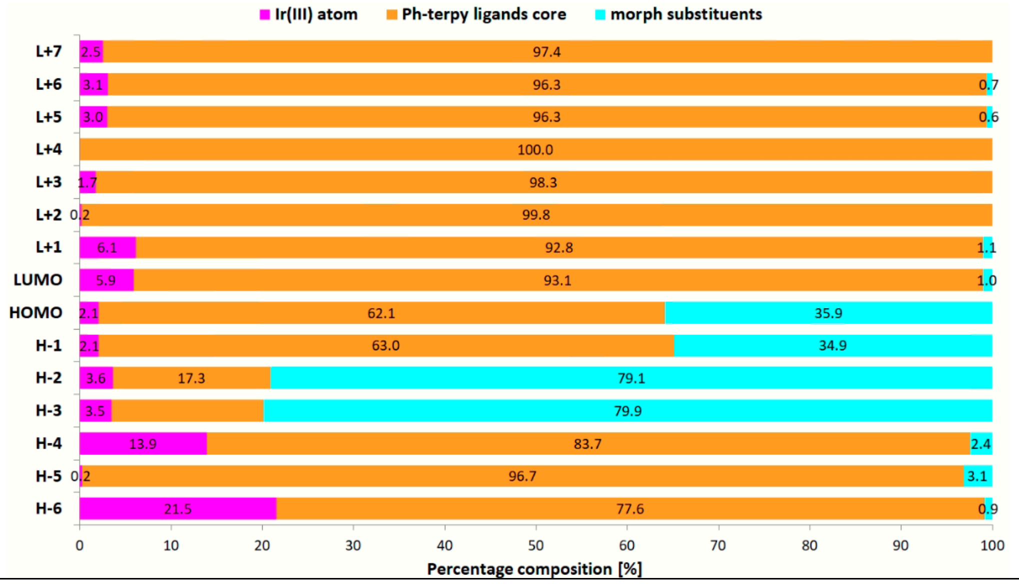

Exp. absorption λ; nm (10^4 ε; $M^{-1}cm^{-1}$)	Calculated transitions					
	Major contribution (%)	Character	E [eV]	λ [nm]	Oscillator strength	transition No.
469.0 (4.95)	HOMO→LUMO (53%)<br>H-1→L+1 (46%)	ILCT	2.39	519.74	1.1681	S1
	H-1→L+1 (53%)<br>HOMO→LUMO (46%)	ILCT	2.49	497.79	0.0015	S3
322.0 (5.43)	H-1→L+5 (48%)<br>HOMO→L+6 (48%)	ILCT	3.64	340.81	0.6448	S11
	H-6→LUMO (44%)<br>H-4→LUMO (41%)	IL/MLCT	3.66	338.47	0.0229	S13
	H-6→L+1 (44%)<br>H-4→L+1 (41%)	IL/MLCT	3.67	338.03	0.0219	S14

2B

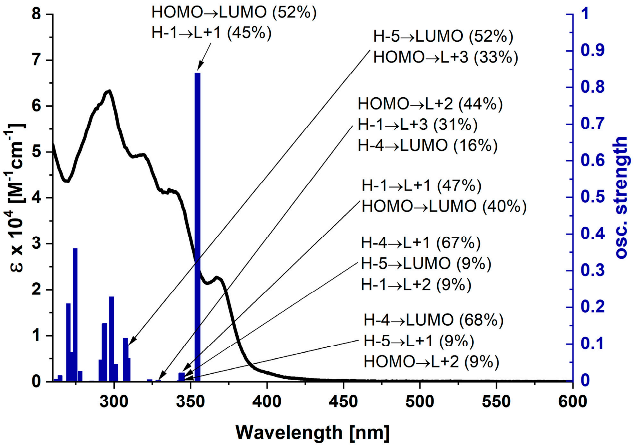

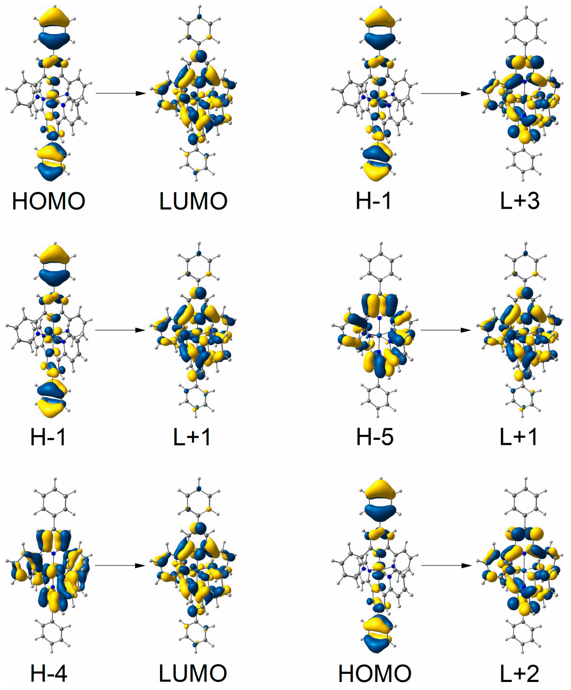

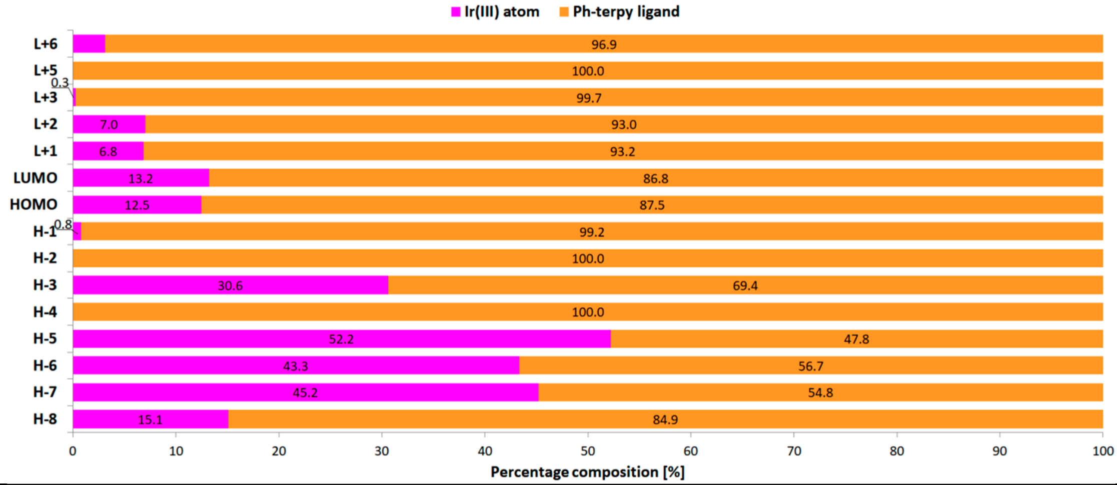

Exp. absorption λ; nm (10^4 ε; $M^{-1}cm^{-1}$)	Calculated transitions					
	Major contribution (%)	Character	E [eV]	λ [nm]	Oscillator strength	transition No.
368.8 (2.27)	H-1→L+1 (45%)<br>HOMO→LUMO (52%)	IL<br>IL/MLCT	3.50	354.62	0.8401	S1
339.8 (4.18)	H-4→LUMO (68%)<br>H-1→L+3 (6%)<br>H-5→L+1 (9%)<br>HOMO→L+2 (9%)	IL<br>IL<br>MLCT/IL<br>IL/MLCT	3.60	344.40	0.0218	S3
	H-4→L+1 (67%)<br>H-5→LUMO (9%)<br>H-1→L+2 (9%)<br>HOMO→L+3 (7%)	IL<br>MLCT/IL<br>IL<br>IL/MLCT	3.60	344.04	0.0205	S4
	H-1→L+1 (47%)<br>HOMO→LUMO (40%)<br>H-8→LUMO (5%)<br>H-7→L+1 (6%)	IL<br>IL/MLCT<br>IL<br>IL/MLCT	3.62	342.92	0.0014	S5
	H-4→LUMO (16%)<br>H-1→L+3 (31%)<br>HOMO→L+2 (44%)	IL<br>IL<br>IL/MLCT	3.77	329.08	0.0027	S7

	H-6→LUMO (5%)	IL/MLCT				
	H-4→L+1 (17%),<br>H-1→L+2 (42%),<br>HOMO→L+3 (31%)<br>H-6→L+1 (5%)	IL<br>IL<br>IL/MLCT<br>IL/MLCT	3.77	328.98	0.0027	S8
	H-3→LUMO (50%)<br>H-2→L+1 (44%)	IL/MLCT<br>IL	3.83	323.39	0.0043	S9
317.8 (4.87)	H-5→LUMO (32%)<br>H-1→L+2 (38%)<br>HOMO→L+3 (22%)	MLCT/IL<br>IL<br>IL/MLCT	4.01	309.10	0.0607	S11
	H-5→L+1 (25%)<br>H-1→L+3 (27%)<br>HOMO→L+2 (39%)	MLCT/IL<br>IL<br>IL/MLCT	4.01	309.05	0.0488	S12
	H-5→LUMO (52%),<br>HOMO→L+3 (33%)<br>H-1→L+2 (6%)	MLCT/IL<br>IL/MLCT<br>IL	4.03	307.83	0.1007	S13
	H-5→L+1 (58%)<br>H-1→L+3 (29%)	MLCT/IL<br>IL	4.03	307.47	0.1168	S14
297.7 (6.35)	H-3→L+3 (38%)<br>H-2→L+2 (59%)	IL/MLCT<br>IL	4.12	301.03	0.0448	S17
	H-4→L+2 (83%)<br>H-6→L+2 (8%)	IL<br>IL/MLCT	4.16	298.38	0.2286	S19
	H-6→LUMO (86%)<br>H-4→LUMO (8%)	IL/MLCT<br>IL	4.22	293.96	0.1565	S21
	H-6→L+1 (86%)<br>H-4→L+1 (8%)	IL/MLCT<br>IL	4.22	293.60	0.1544	S22
	H-8→LUMO (42%)<br>H-7→L+1 (43%)<br>H-5→L+3 (5%)	IL/MLCT<br>IL/MLCT<br>MLCT/IL	4.25	291.45	0.0567	S23
	H-5→L+3 (86%)	MLCT/IL	4.51	274.69	0.3604	S31
	H-8→L+3 (37%)<br>H-7→L+2 (56%)	IL/MLCT<br>IL/MLCT	4.56	271.92	0.0736	S32
	H-8→L+2 (53%)<br>H-7→L+3 (39%)	IL/MLCT<br>IL/MLCT	4.56	271.79	0.0776	S33
	H-6→L+2 (80%)<br>H-4→L+2 (11%)	IL/MLCT<br>IL	4.59	270.22	0.2099	S34
	H-1→L+5 (46%)<br>HOMO→L+6 (48%)	IL<br>IL/MLCT	4.86	254.86	0.4692	S42

Table S7. Triplet energies calculated from the energy difference between the ground singlet and triplet excited states $\Delta E_{T_1-S_0}$ compared to the experimental values for complexes **1A, 1B, 2A** and **2B.**

**Complex**	**DFT $\Delta E_{T_1-S_0}$**		**λ_{exp}**
	**eV / nm**	**Character**	**eV / nm**
**1A**	1.78 / 694	MLCT-ILCT	2.02 / 614
**1B**	2.10 / 590	MLLCT	2.05 / 604
**2A**	1.49 / 832	ILCT	1.63 / 760
**2B**	2.30 / 540	IL-MLCT	2.14 / 580

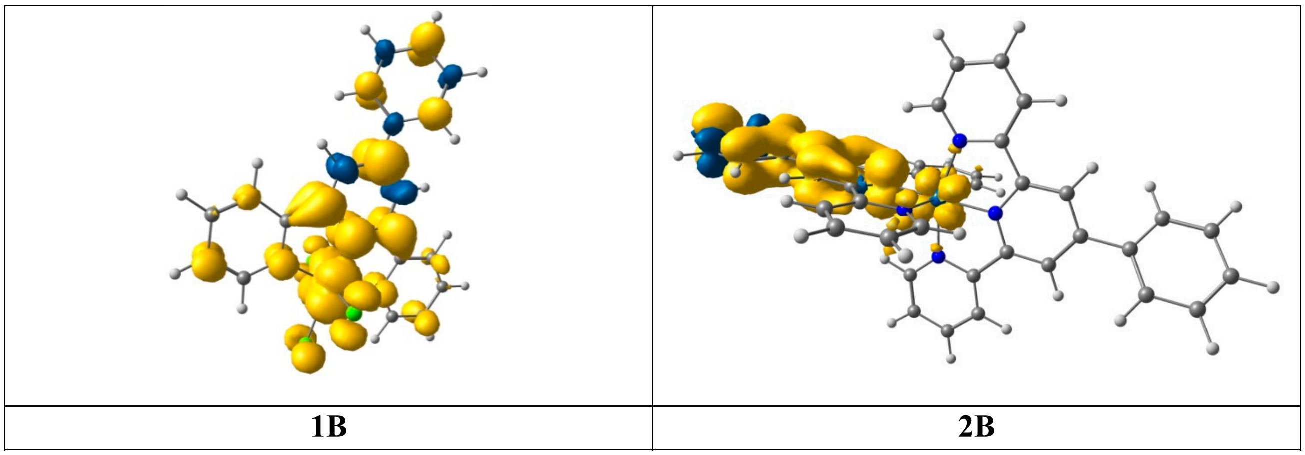

1B	2B

Figure S7. Spin density surface plots for **1B** and **2B.**

UV-VIS SPECTROSCOPY

Table S8. The absorption maxima and molar extinction coefficient values for the complexes **1–2.**

Compound	Medium	λ/nm (10^4 ε/ $dm^3 \cdot mol^{-1} \cdot cm^{-1}$)
1A	MeCN	520.6 (0.41), 488.6 (0.45), 427.5 (1.11), 378.3 (1.03), 323.7 (2.28), 313.1 (2.36), 288.8 (2.61)
	DMSO	528.9 (0.37), 492.7 (0.39), 441.1 (0.93), 421.6 (0.97), 381.9 (0.92), 324.9 (2.16), 311.94 (2.34), 289.3 (2.78), 273.4 (2.35)
1B	MeCN	523.6 (0.18), 489.4 (0.17), 418.7 (0.54), 328.3 (1.97), 312.5 (2.29), 285.3 (3.05), 272.6 (2.63)
	DMSO	525.9 (0.12), 458.9 (0.11), 415.8 (0.38), 332.9 (1.54), 318.4 (1.84), 289.9 (2.93), 277.24 (2.63)
2A	MeCN	469.6 (4.20), 322.0 (5.43), 309.5 (5.98), 282.2 (6.78)
	DMSO	469.0 (4.95), 312.5 (7.23), 289.3 (7.34), 262.7 (6.33)
2B	MeCN	520.6 (0.01), 450.0 (0.02), 368.8 (2.27), 339.8 (4.18), 317.8 (4.87), 297.7 (6.35)
	DMSO	520.6 (0.006), 456.6 (0.01), 374.7 (2.55), 347.5 (4.57), 324.4 (5.47), 300.1 (7.19), 263.9 (6.07)

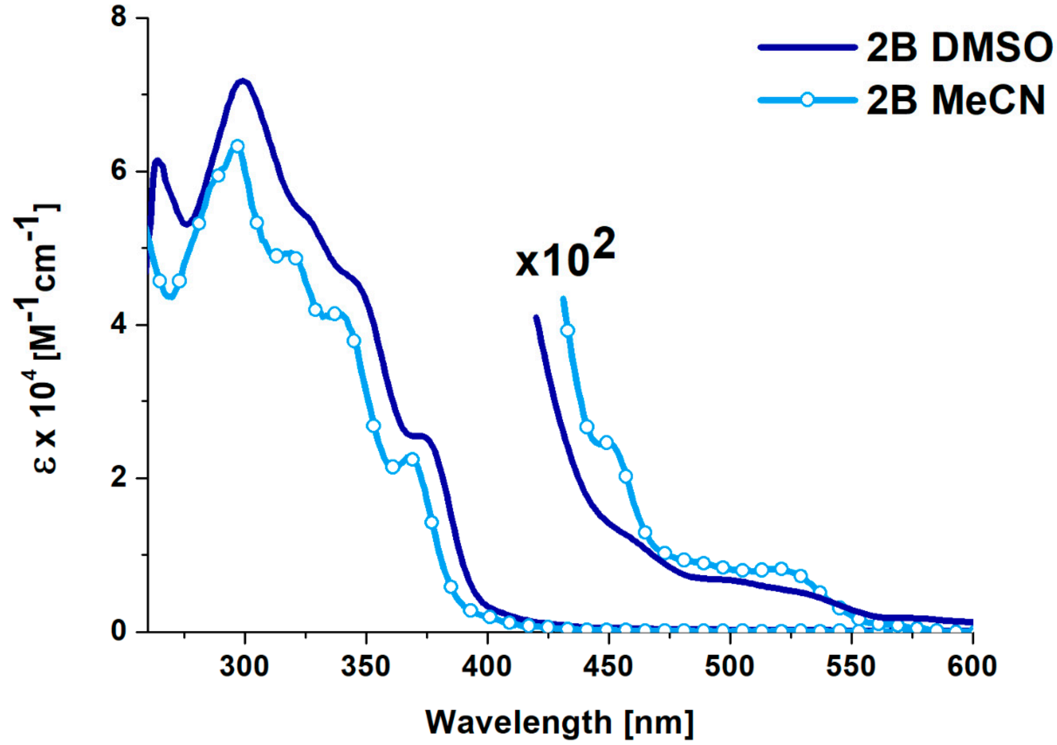

Figure S8. The absorption spectra of **2B** recorded in $2.5 \cdot 10^{-5}$ and $5 \cdot 10^{-4}$ mol/dm^3 concentration.

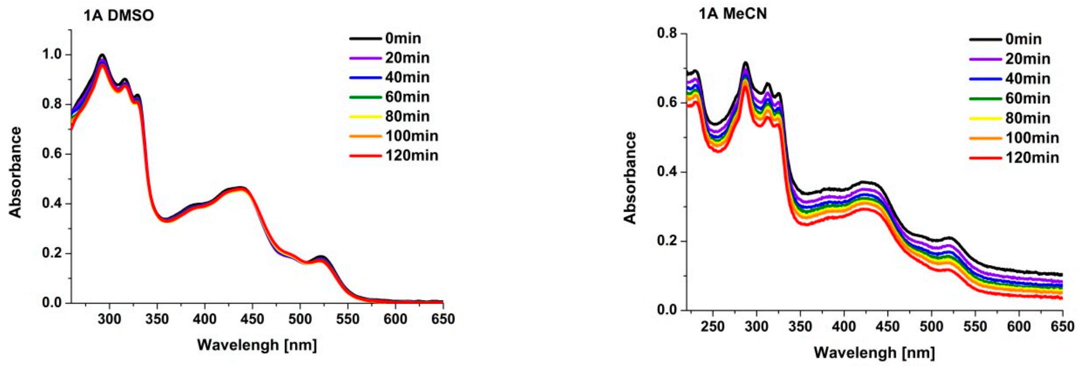

(a)

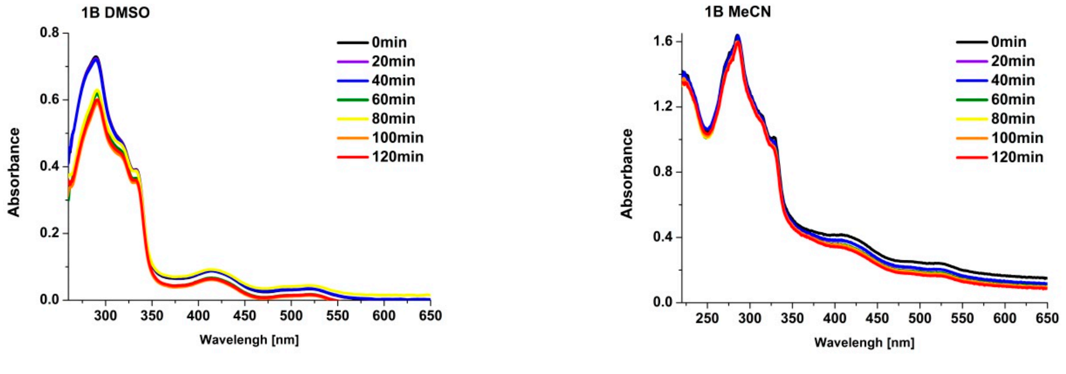

(b)

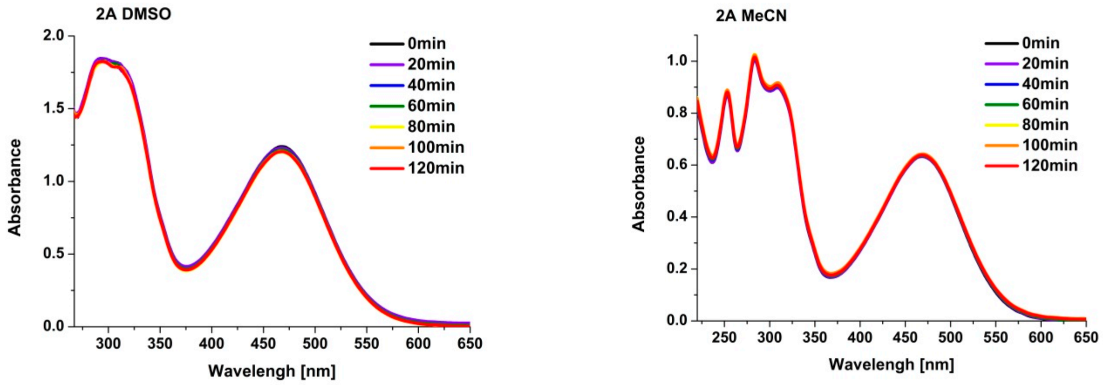

(c)

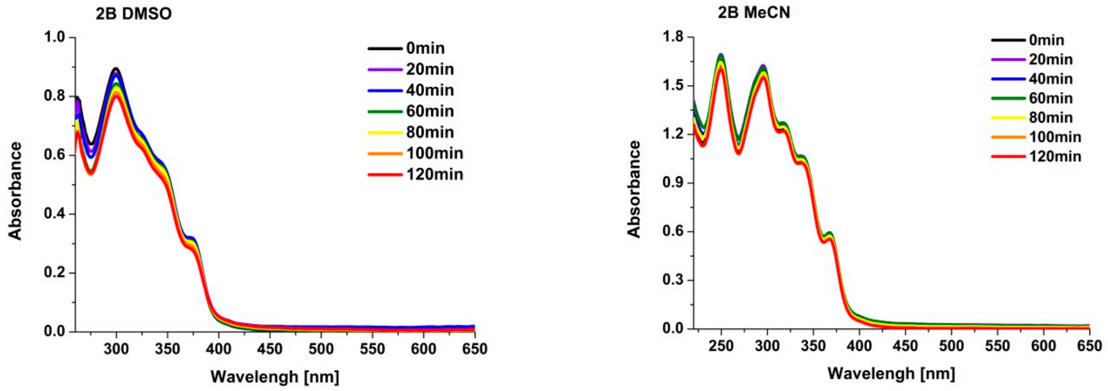

(d)

Figure S9. The photostability of **1A** (a), **1B** (b), **2A** (c), **2B** (d) in DMSO and MeCN solutions.

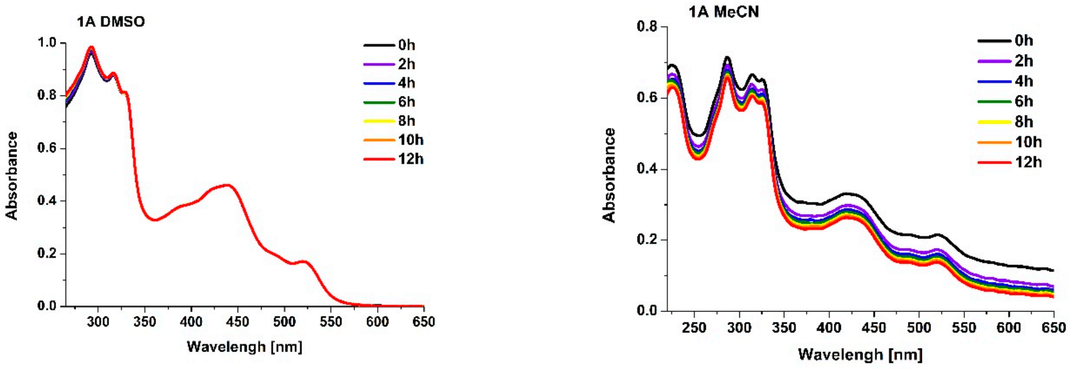

(a)

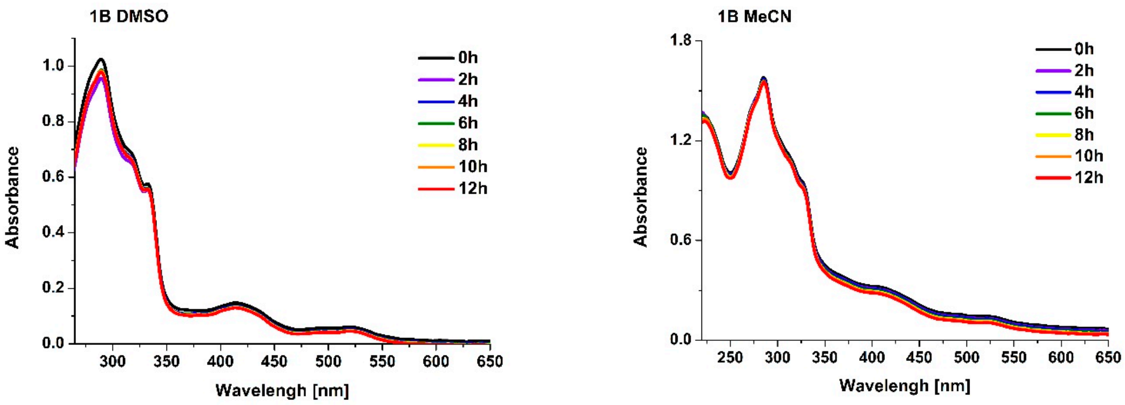

(b)

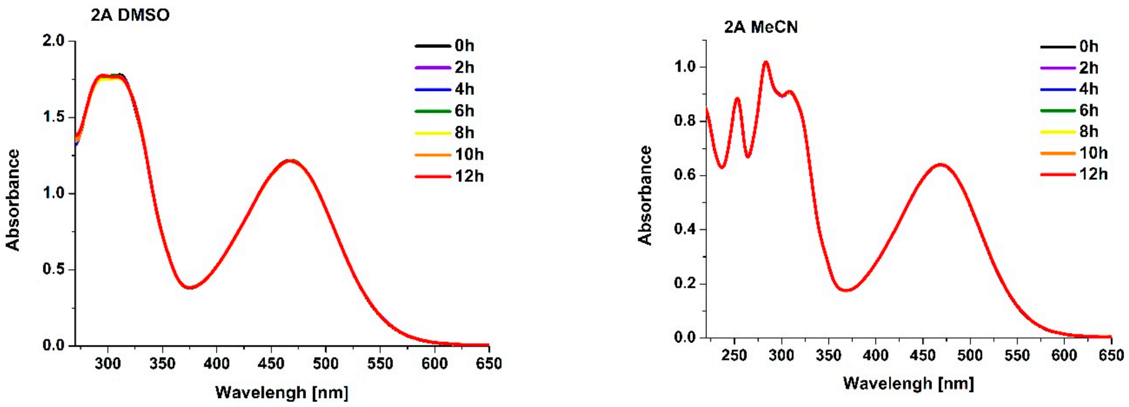

(c)

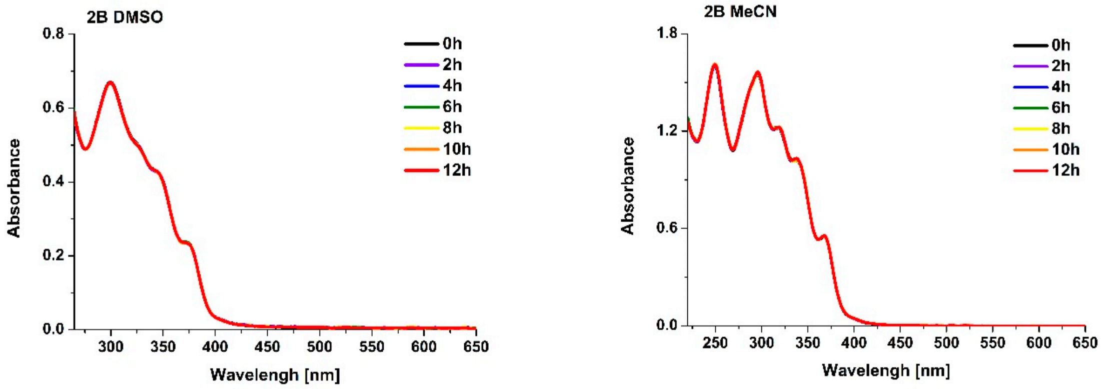

(d)

Figure S10. The kinetic stability of **1A** (a), **1B** (b), **2A** (c), **2B** (d) in DMSO and MeCN solutions in 2h intervals for 12h.

LUMINESCENCE PROPERTIES

1A

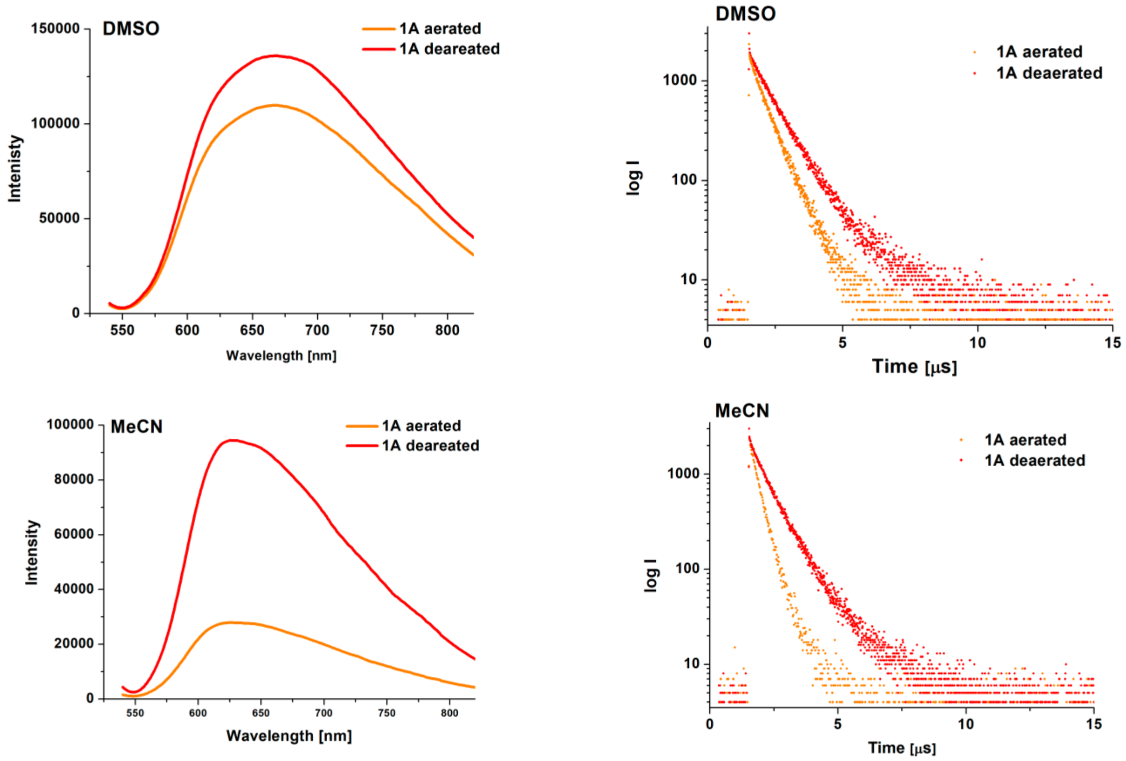

1B

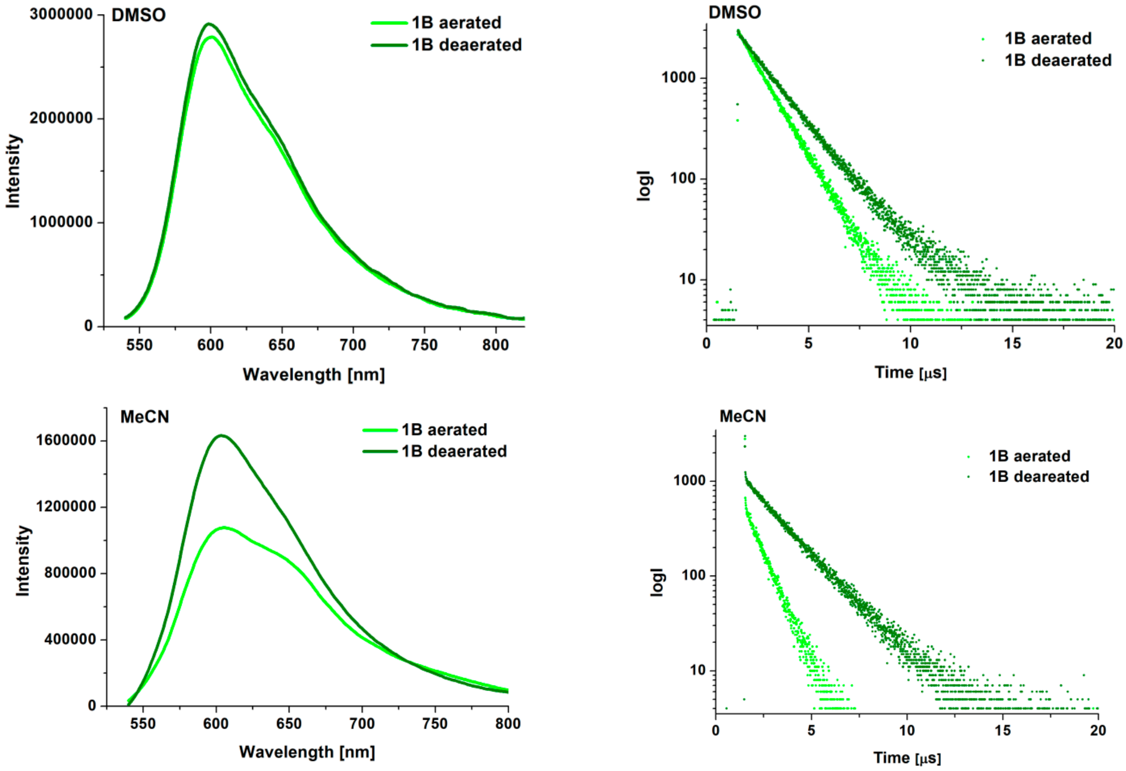

Figure S11. The emission spectra and decay curves of **1A** and **1B** in an aerated and deareted solutions.

2A

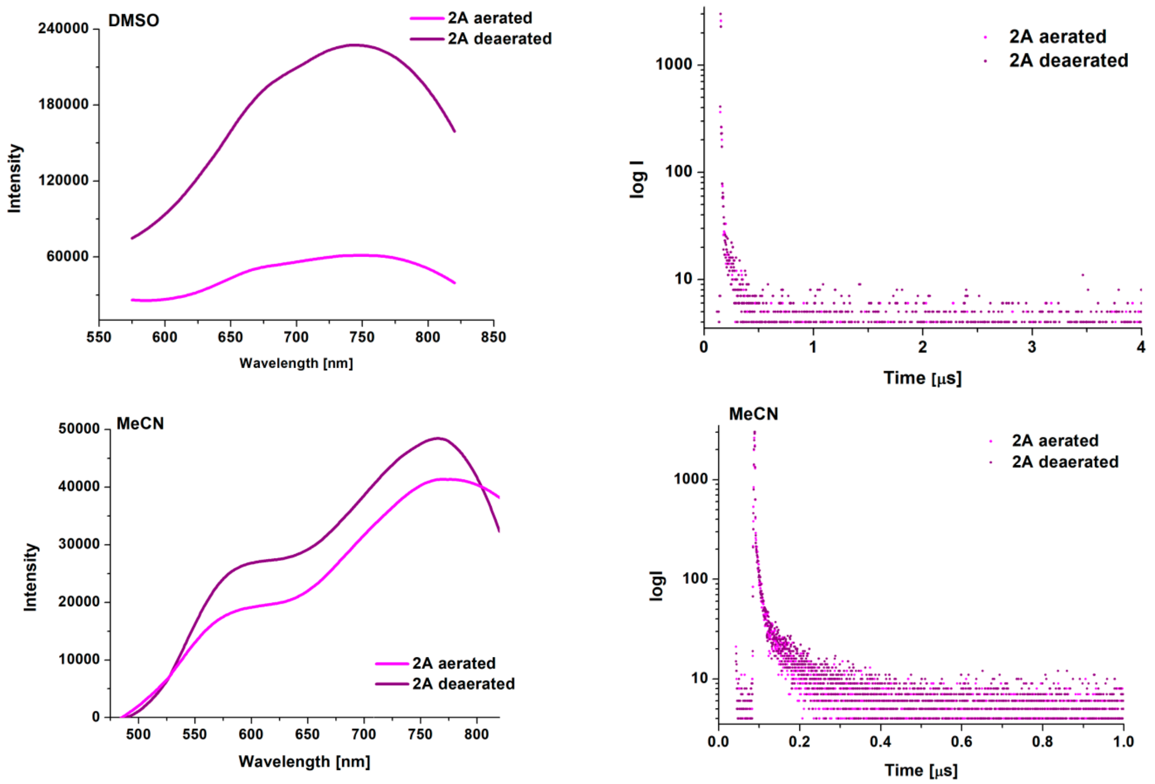

2B

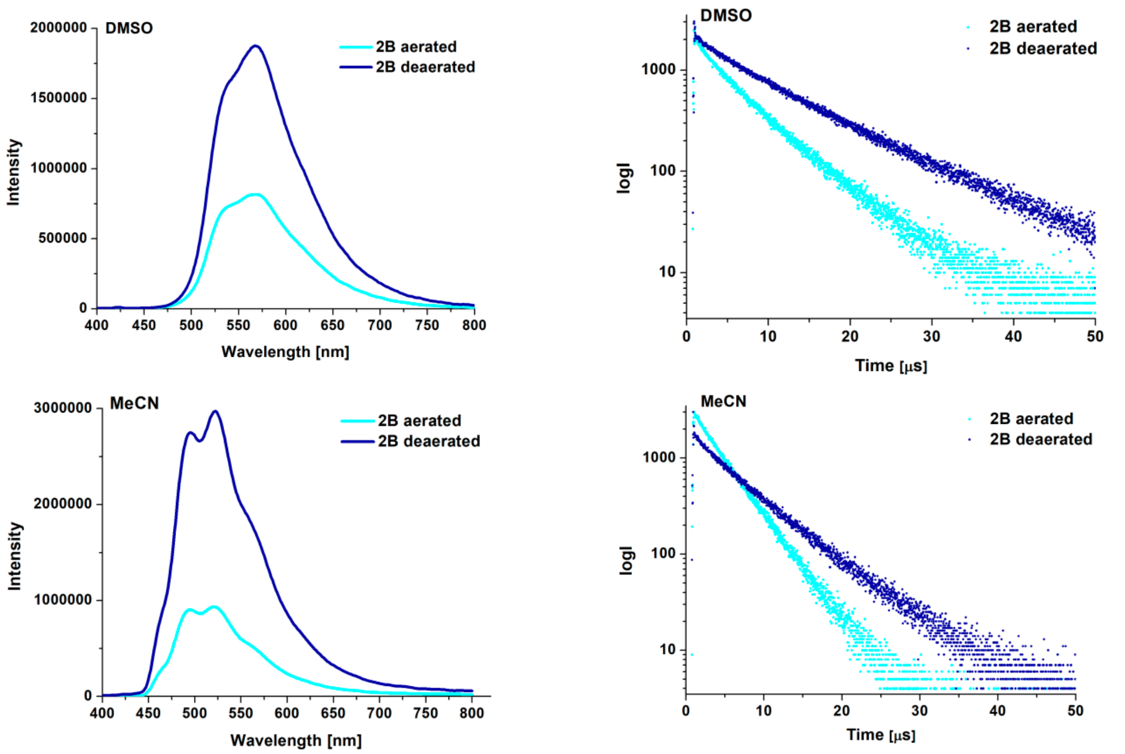

Figure S12. The emission spectra and decay curves of **2A** and **2B** in an aerated and deareted solutions.

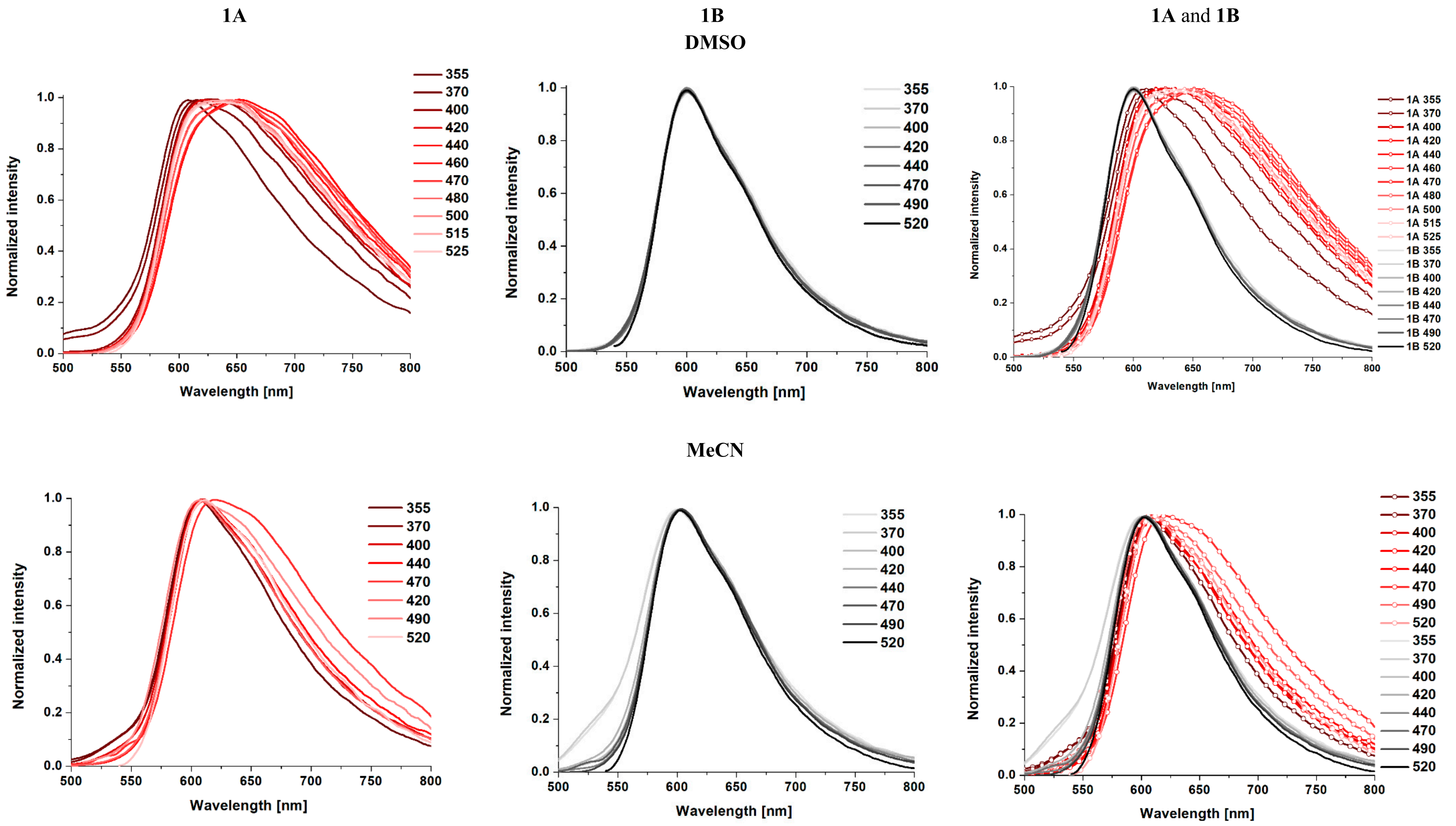

Figure S13. Excitation-dependent emission spectra of **1A** and **1B** in DMSO and MeCN.

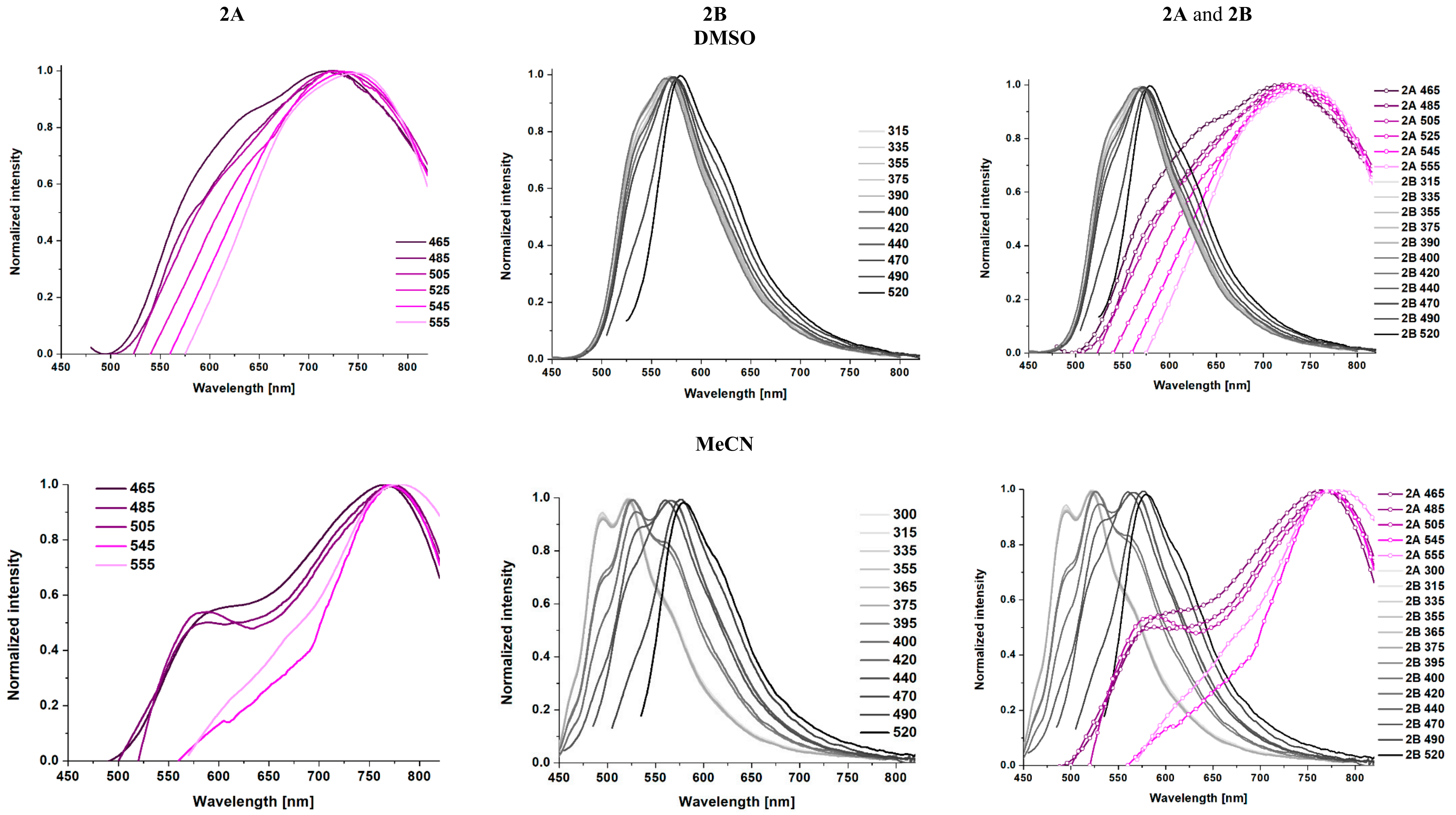

Figure S14. Excitation-dependent emission spectra of **2A** and **2B** in DMSO and MeCN.

77 K

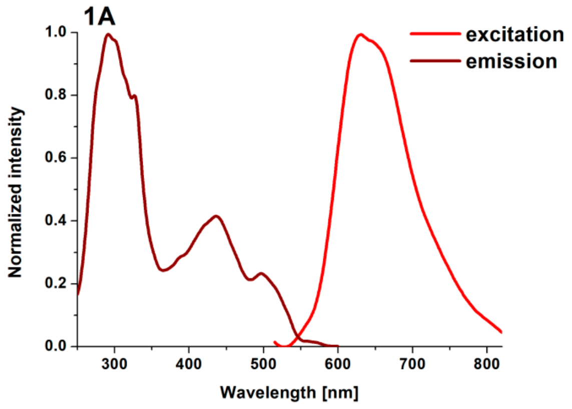

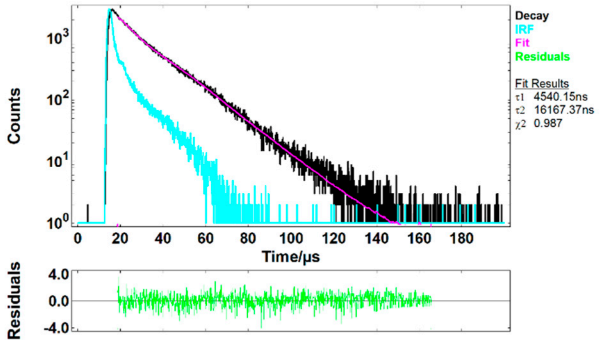

Fit results: τ_1 = 4.540 µs ± 0.128 µs (45.32 %)
τ_2 = 16.167 µs ± 0.141 µs (54.68 %)

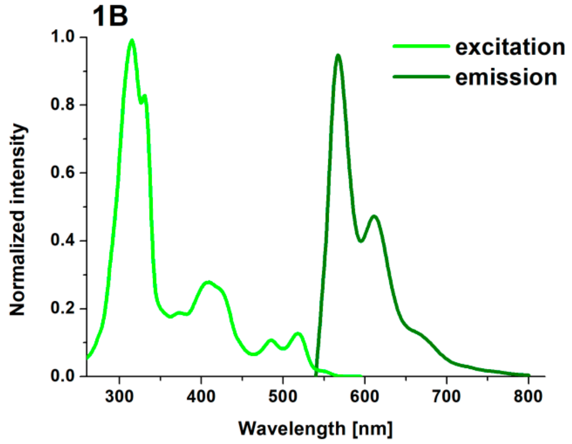

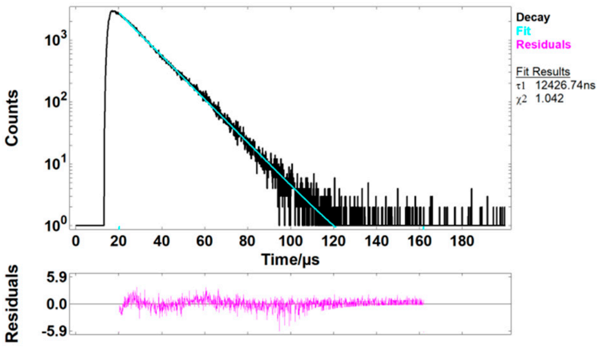

Fit results: τ_1 = 12.427 µs ± 0.022 µs

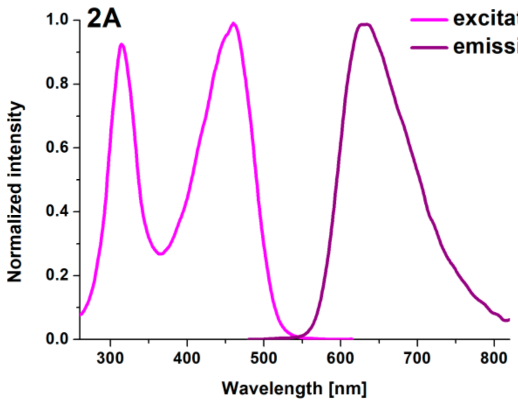

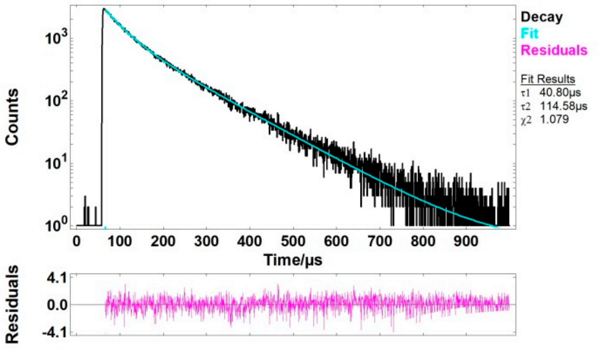

Fit results: τ_1 = 40.800 µs ± 0.653 µs (32.71 %)
τ_2 = 114.580 µs ± 0.777 µs (67.29 %)

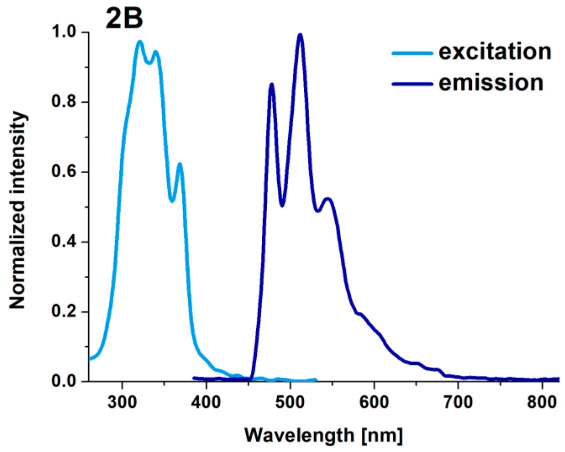

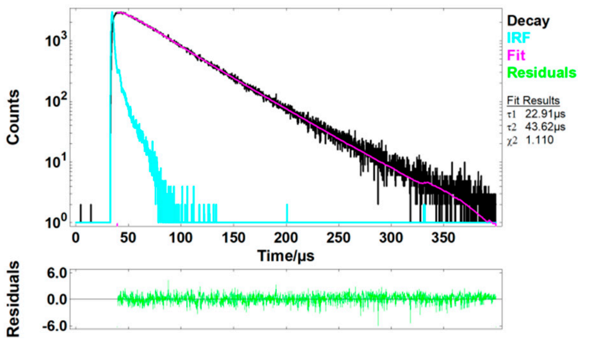

Fit results: τ_1 = 22.910 μs ± 1.798 μs (17.09 %)
τ_2 = 43.620 μs ± 0.487 μs (82.91 %)

Figure S15. Absorbance, excitation and emission steady-state spectra of **1**–**2** in EtOH:MeOH glass matrix at 77K with the corresponding decay curves.

DMSO

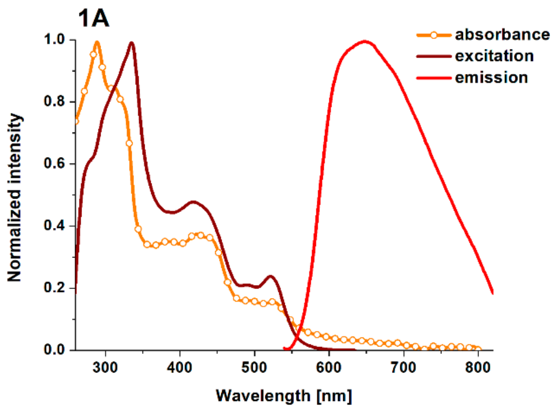

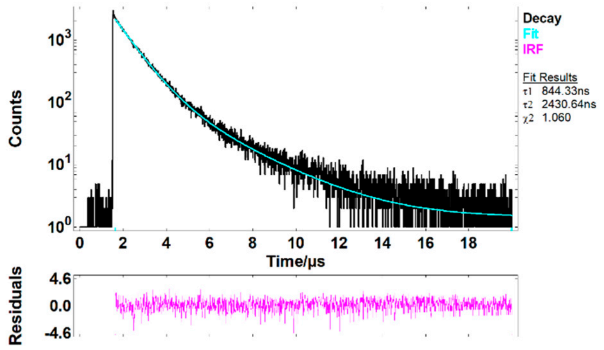

Fit results: τ_1 = 0.844 μs ± 0.007 μs (76.54 %)
τ_2 = 2.431 μs ± 0.056 μs (23.46 %)

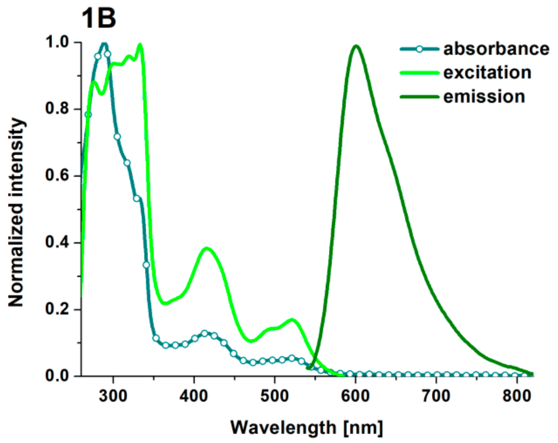

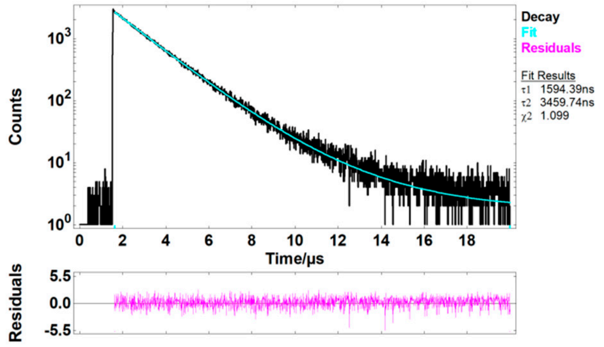

Fit results: τ_1 = 1.594 μs ± 0.016 μs (91.65 %)
τ_2 = 3.460 μs ± 0.422 μs (8.35 %)

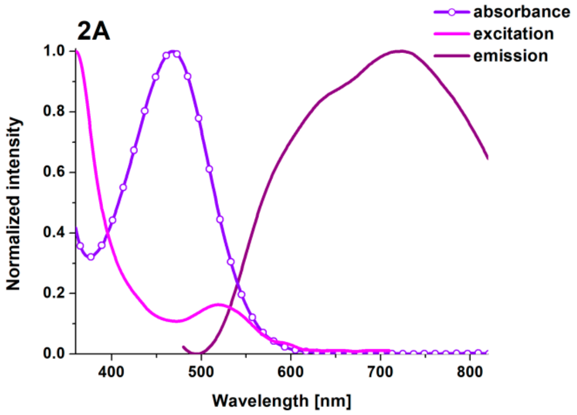

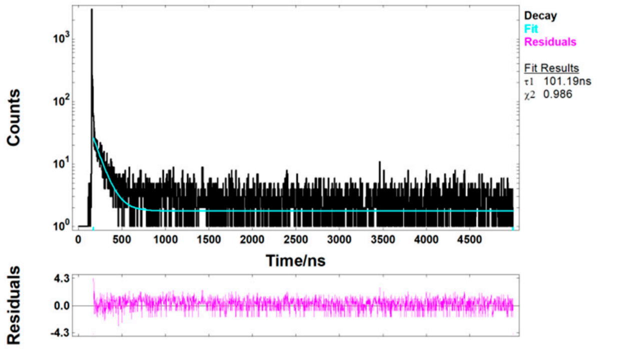

Fit results: τ_1 = 0.101 μs ± 0.005 μs

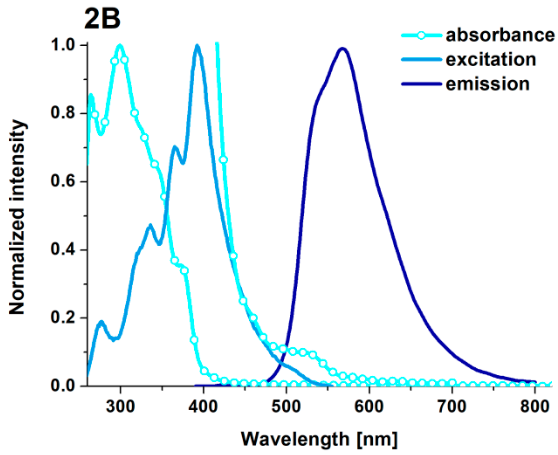

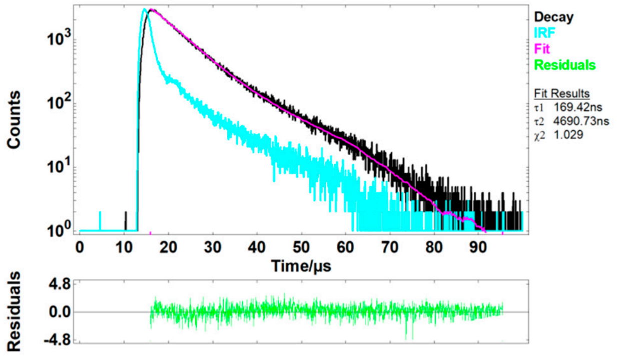

Fit results: τ_1 = 0.169 μs ± 0.244 μs (5.05 %)
τ_2 = 4.691 μs ± 0.016 μs (94.95 %)

Figure S16. Absorbance, excitation and emission steady-state spectra of **1–2** in **deaerated** DMSO at RT with the corresponding decay curves.

MeCN

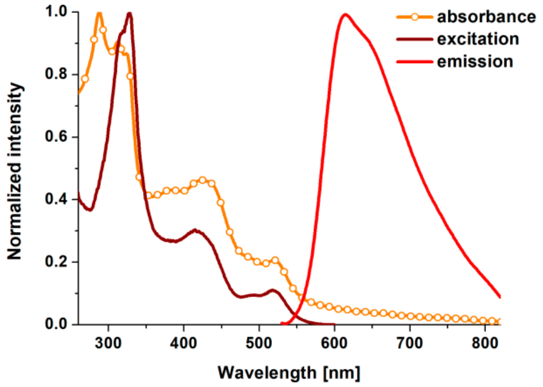

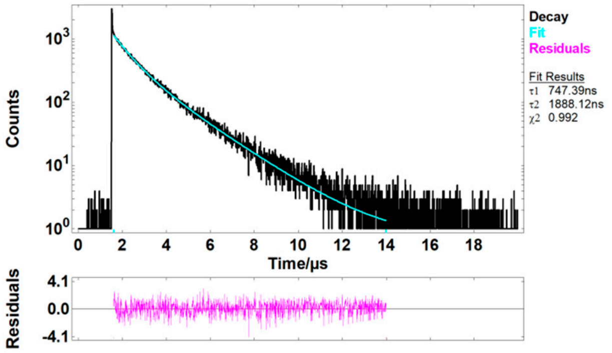

Fit results: τ_1 = 0.747 ± 0.024 μs (38.56 %)
τ_2 = 1.888 μs ± 0.039 μs (61.44 %)

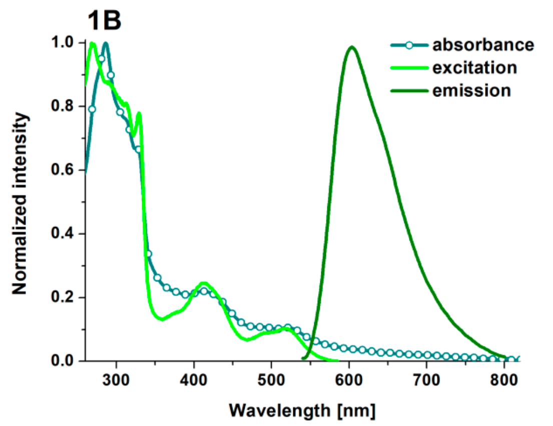

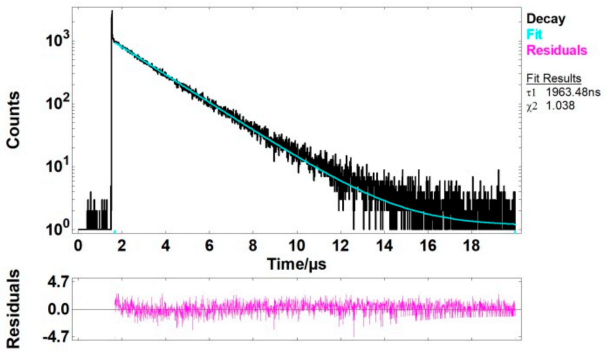

Fit results: τ_1 = 1.963 ± 0.005 μs

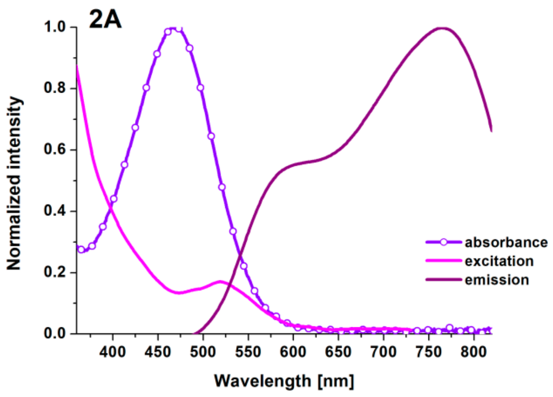

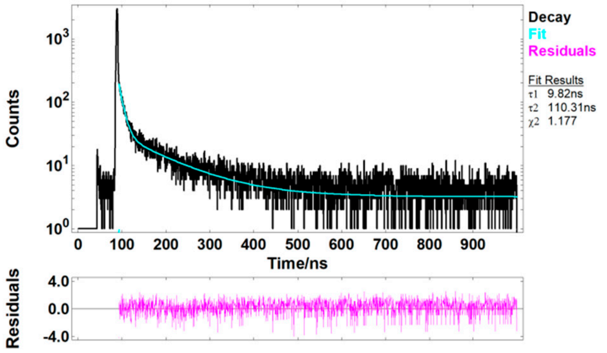

Fit results: τ_1 = 0.010 μs ± 0.001 μs (35.96 %)
τ_2 = 0.110 μs ± 0.004 μs (64.04 %)

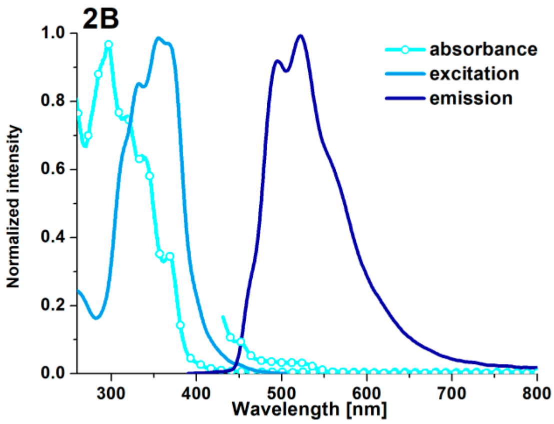

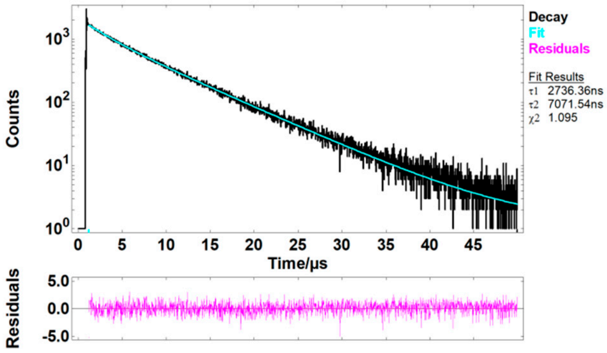

Fit results: τ_1 = 2.736 μs ± 0.142 μs (13.30 %)
τ_2 = 7.072 μs ± 0.059 μs (86.70 %)

Figure S17. Absorbance, excitation and emission steady-state spectra of **1**–**2** in deaerated MeCN at RT with the corresponding decay curves.

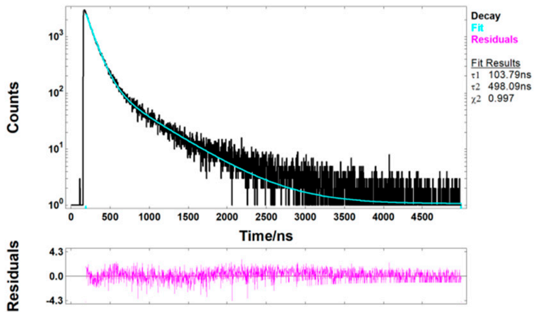

Fit results: τ_1 = 0.104 μs ± 0.001 μs (73.41 %)
τ_2 = 0.498 μs ± 0.007 μs (26.59 %)

1A

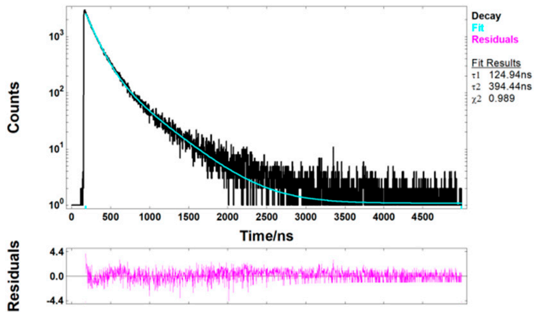

Fit results: τ_1 = 0.125 μs ± 0.001 μs (67.25 %)
τ_2 = 0.394 μs ± 0.005 μs (32.75 %)

1B

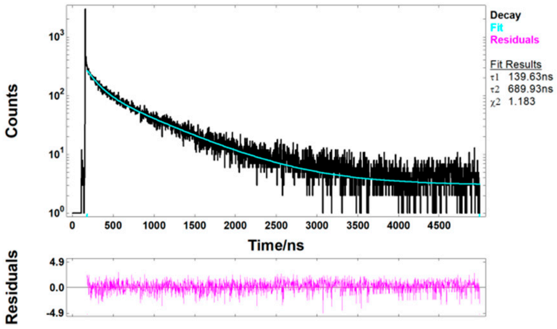

Fit results: τ_1 = 0.140 μs ± 0.007 μs (19.35 %)
τ_2 = 0.690 μs ± 0.012 μs (80.65 %)

2A

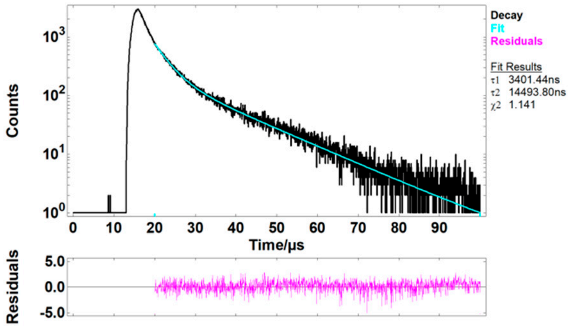

Fit results: τ_1 = 3.401 μs ± 0.068 μs (37.86 %)
τ_2 = 14.494 μs ± 0.217 μs (62.14 %)

2B

Figure S18. Decay curves of **1–2** in solid state.

FEMTOSECOND TRANSIENT ABSORPTION

1A

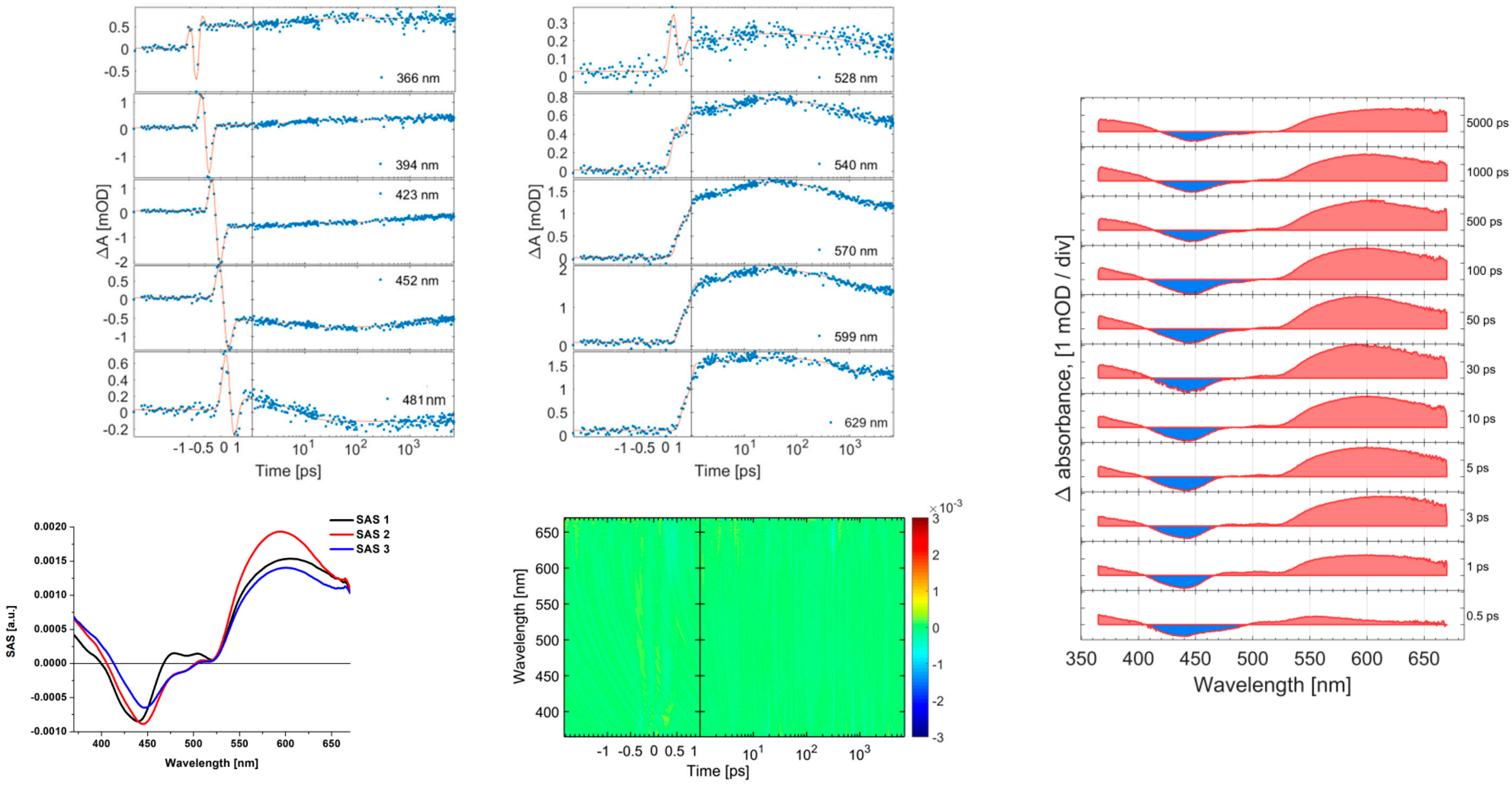

Figure S19. Summary of the TA analysis of **1A** (pump wavelength 355 nm; pump power 0.24 μJ per pulse) containing time traces at several wavelength, evolution associated spectra, residual map and transient spectra.

1B

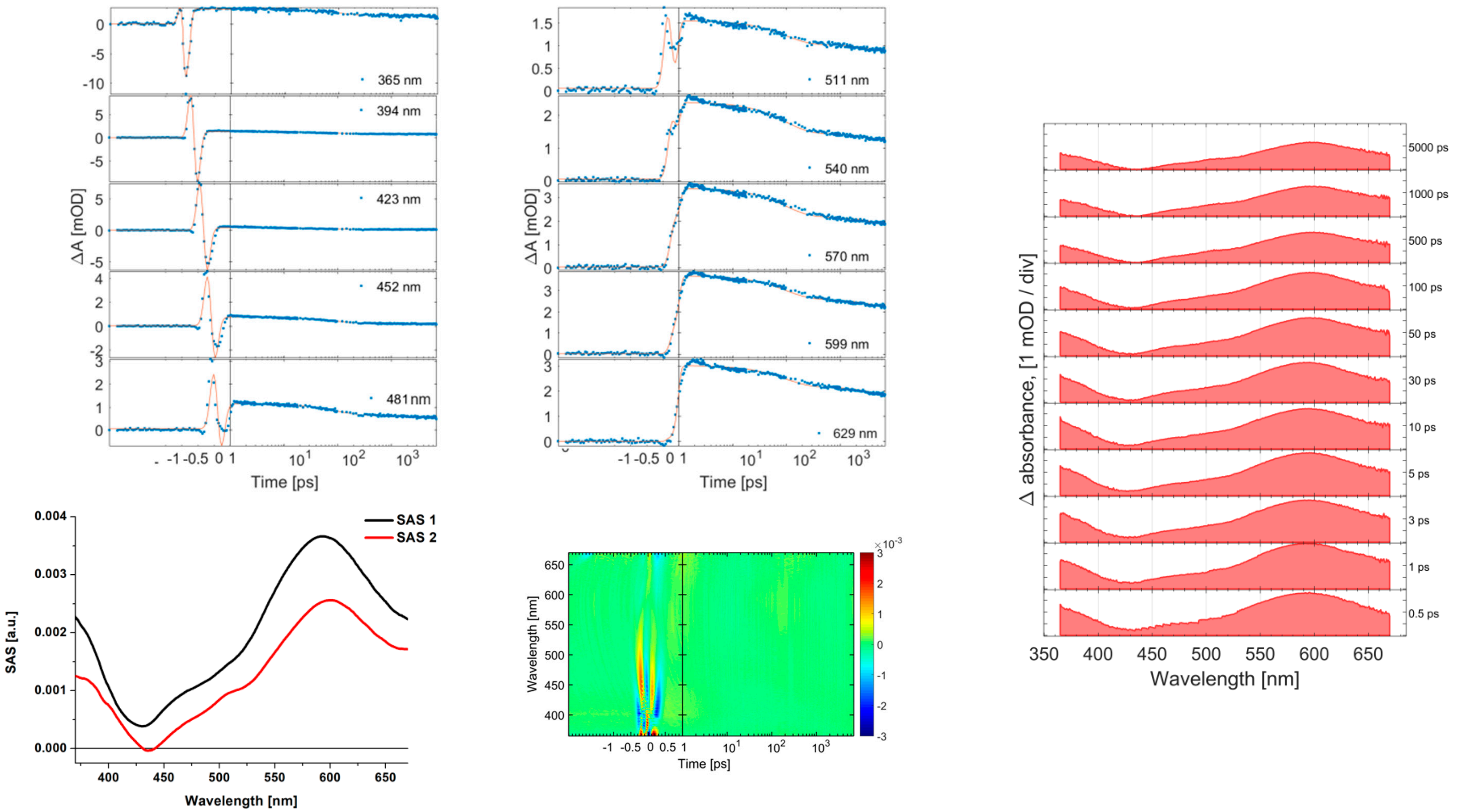

Figure S20. Summary of the TA analysis of **1B** (pump wavelength 355 nm; pump power 0.24 μJ per pulse) containing time traces at several wavelength, evolution associated spectra, residual map and transient spectra.

2A

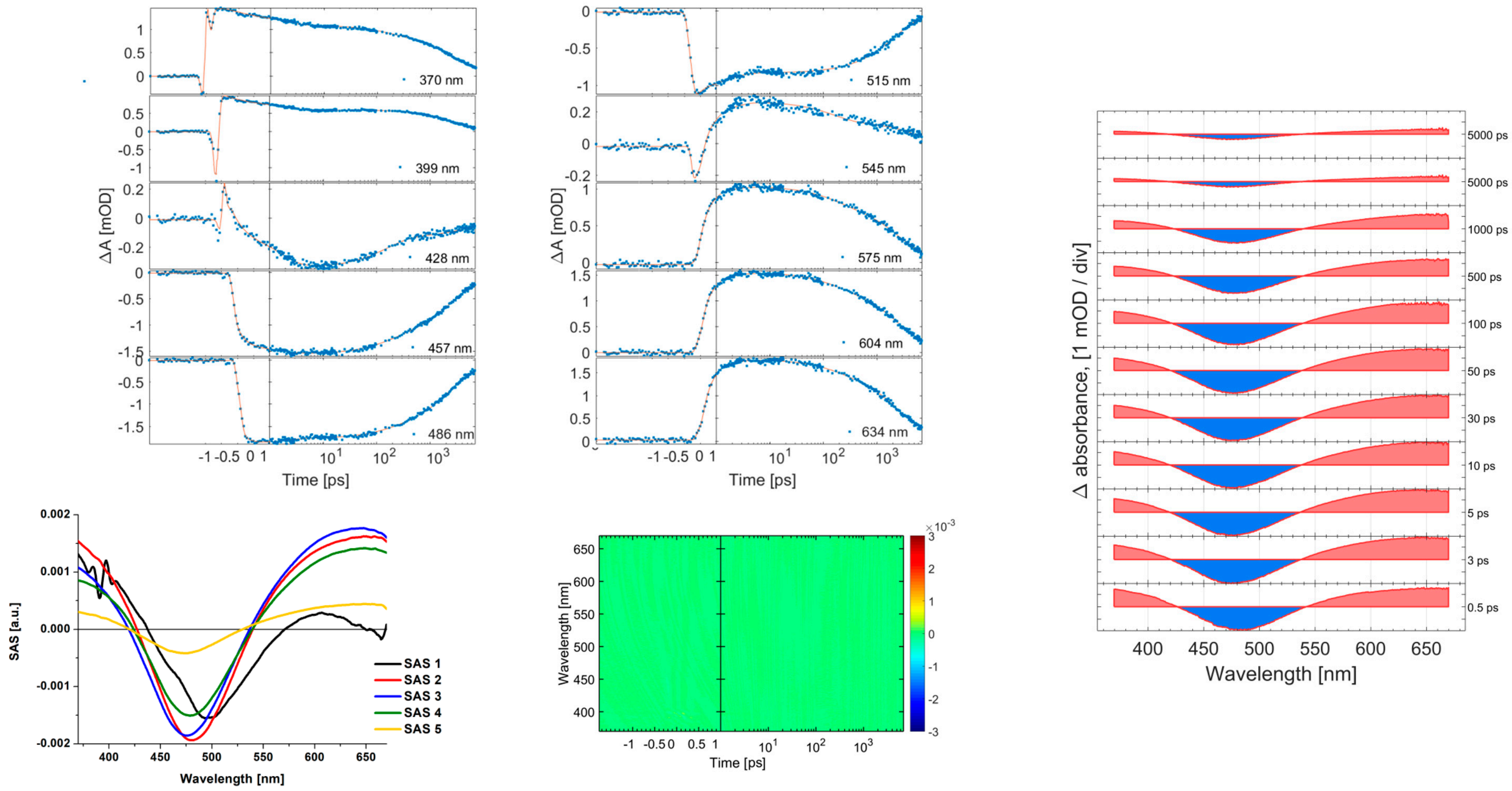

Figure S21. Summary of the global lifetime analysis of **2A** (pump wavelength 355 nm; pump power 0.24 µJ per pulse) containing time traces at several wavelength, evolution associated spectra, residual map and transient spectra.

2B

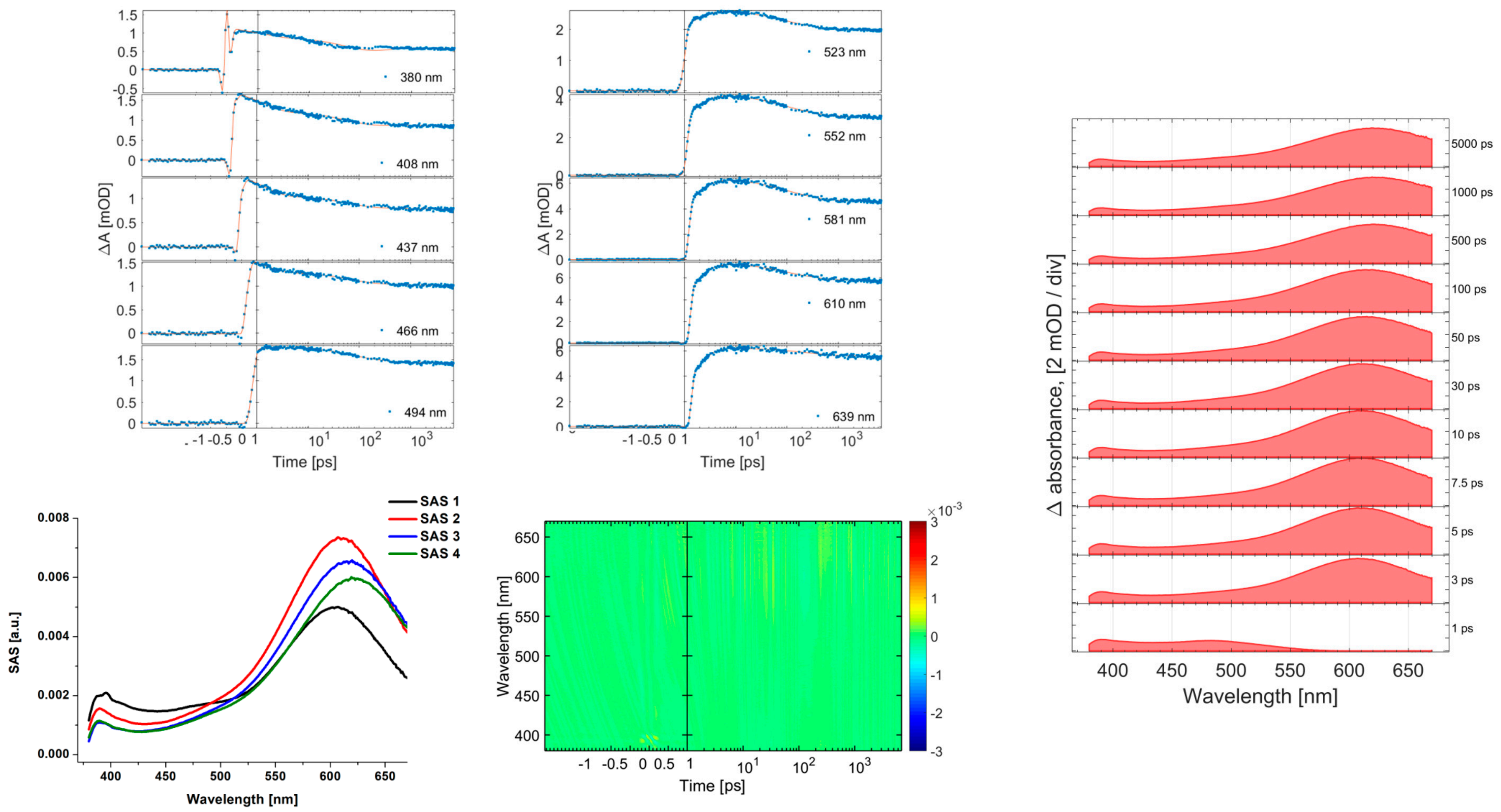

Figure S22. Summary of the global lifetime analysis of **2B** (pump wavelength 355 nm; pump power 0.24 µJ per pulse) containing time traces at several wavelength, evolution associated spectra, residual map and transient spectra.

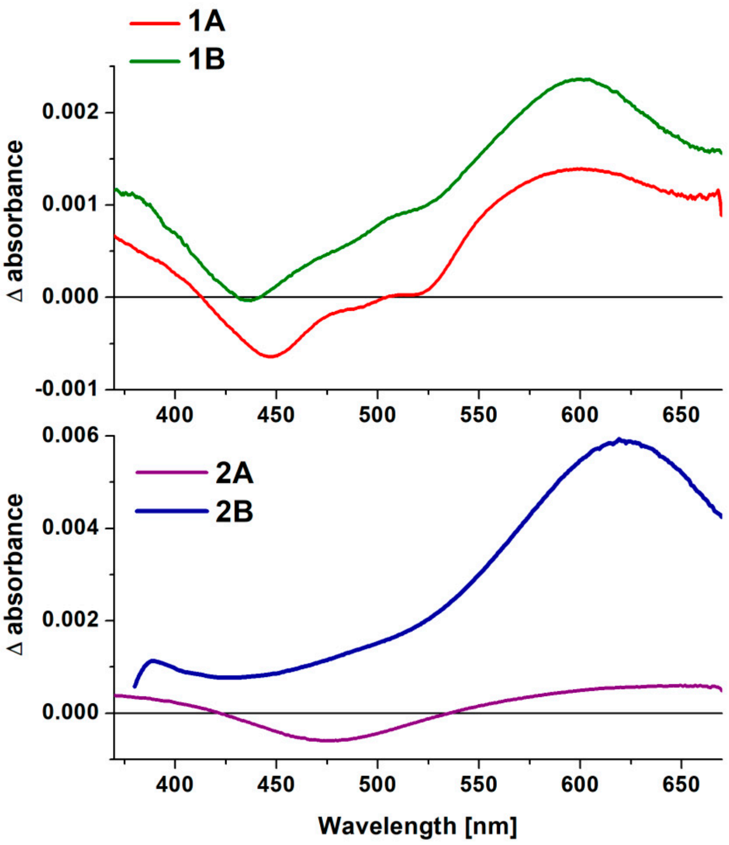

Figure S23. Comparison of the TA spectral profiles of investigated Ir(III) complexes at 3ns delay time.

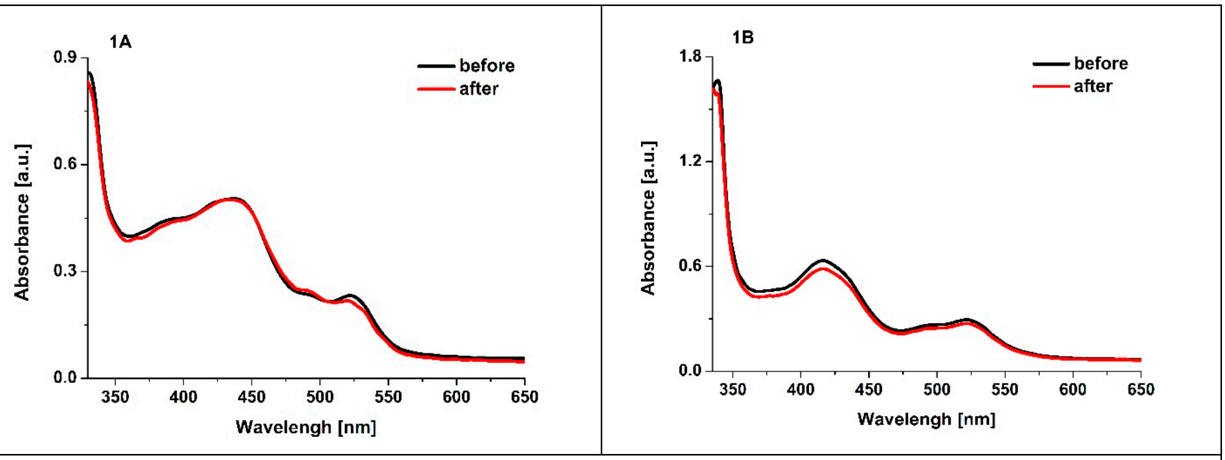

(a)

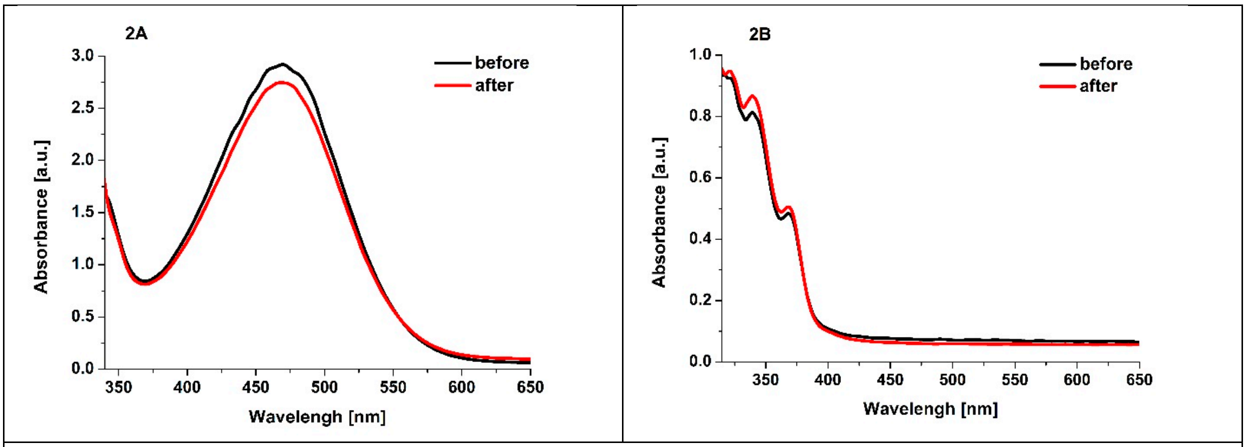

(b)

Figure S24. The photodamage test for **1A** and **1B** in DMSO (a) and **2A** and 2**B** in MeCN (b).

References

1. CrysAlis PRO 2011.

2. Sheldrick, G.M. Crystal Structure Refinement with SHELXL. *Acta Crystallogr. Sect. C Struct. Chem.* **2015**, *71*, 3–8, doi:10.1107/S2053229614024218.

3. Frisch, M.J.; Trucks, G.W.; Schlegel, H.B.; Scuseria, G.E.; Robb, M.A.; Cheeseman, J.R.; Scalmani, G.; Barone, V.; Petersson, G.A.; Nakatsuji, H.; et al. Gaussian 16 Rev. C.01 2016.

4.. Andrae, D.; Häußermann, U.; Dolg, M.; Stoll, H.; Preuß, H. Energy-Adjustedab Initio Pseudopotentials for the Second and Third Row Transition Elements. Theor. Chim. Acta 1990, 77, 123–141, doi:10.1007/BF01114537.

5. Martin, J.M.L.; Sundermann, A. Correlation Consistent Valence Basis Sets for Use with the Stuttgart–Dresden–Bonn Relativistic Effective Core Potentials: The Atoms Ga–Kr and In–Xe. J. Chem. Phys. 2001, 114, 3408–3420, doi:10.1063/1.1337864.

6. Pritchard, B.P.; Altarawy, D.; Didier, B.; Gibson, T.D.; Windus, T.L. New Basis Set Exchange: An Open, Up-to-Date Resource for the Molecular Sciences Community. J. Chem. Inf. Model. 2019, 59, 4814–4820, doi:10.1021/acs.jcim.9b00725.

7. Weigend, F.; Ahlrichs, R. Balanced Basis Sets of Split Valence, Triple Zeta Valence and Quadruple Zeta Valence Quality for H to Rn: Design and Assessment of Accuracy. Phys. Chem. Chem. Phys. 2005, 7, 3297–3305, doi:10.1039/B508541A.

8. Klemens, T.; Świtlicka, A.; Szlapa-Kula, A.; Krompiec, S.; Lodowski, P.; Chrobok, A.; Godlewska, M.; Kotowicz, S.; Siwy, M.; Bednarczyk, K.; et al. Experimental and Computational Exploration of Photophysical and Electroluminescent Properties of Modified 2,2′:6′,2″-Terpyridine, 2,6-Di(Thiazol-2-Yl)Pyridine and 2,6-Di(Pyrazin-2-Yl)Pyridine

Ligands and Their Re(I) Complexes. *Appl. Organomet. Chem.* **2018**, *32*, e4611, doi:10.1002/aoc.4611.

9. Szlapa-Kula, A.; Małecka, M.; Maroń, A. M.; Janeczek, H.; Siwy, M.; Schab-Balcerzak, E.; Szalkowski, M.; Maćkowski, S.; Pedzinski, T.; Erfurt, K.; Machura, B. In-Depth Studies of Ground- and Excited-State Properties of Re(I) Carbonyl Complexes Bearing 2,2′:6′,2″-Terpyridine and 2,6-Bis(Pyrazin-2-Yl)Pyridine Coupled with π-Conjugated Aryl Chromophores. Inorg. Chem. 2021, 60 (24), 18726–18738. https://doi.org/10.1021/acs.inorgchem.1c02151.

10. Małecka, M.; Szlapa-Kula, A.; Maroń, A. M.; Ledwon, P.; Siwy, M.; Schab-Balcerzak, E.; Sulowska, K.; Maćkowski, S.; Erfurt, K.; Machura, B. Impact of the Anthryl Linking Mode on the Photophysics and Excited-State Dynamics of Re(I) Complexes [ReCl(CO)3(4′-An-Terpy-κ2N)]. Inorg. Chem. 2022, 61 (38), 15070–15084. https://doi.org/10.1021/acs.inorgchem.2c02160.

10. Wilderen, L. J. G. W. van; Lincoln, C. N.; Thor, J. J. van. Modelling Multi-Pulse Population Dynamics from Ultrafast Spectroscopy. PLOS ONE 2011, 6 (3), e17373. https://doi.org/10.1371/journal.pone.0017373.
